# Semisynthesis, Structure Elucidation and Anti-*Mycobacterium marinum* Activity of a Series of Marine-Derived 14-Membered Resorcylic Acid Lactones with Interesting Ketal Groups

**DOI:** 10.3390/md22100431

**Published:** 2024-09-25

**Authors:** Jun-Na Yin, Cui-Fang Wang, Xiu-Li Zhang, Ya-Jie Cheng, Yan-Wei Wu, Qun Zhang, Chang-Lun Shao, Mei-Yan Wei, Yu-Cheng Gu

**Affiliations:** 1 Key Laboratory of Marine Drugs, The Ministry of Education of China, School of Medicine and Pharmacy, Ocean University of China, Qingdao 266003, China; yinjunna@163.com (J.-N.Y.); wangcuifang0115@163.com (C.-F.W.); xiulizhang@ouc.edu.cn (X.-L.Z.); yajiecheng1212@163.com (Y.-J.C.); wuyanwei1214@163.com (Y.-W.W.); zhangqunnn@163.com (Q.Z.); shaochanglun@163.com (C.-L.S.); 2 Key Laboratory of Tropical Medicinal Resource Chemistry of Ministry of Education, College of Chemistry and Chemical Engineering, Hainan Normal University, Haikou 571158, China; 3 Syngenta Jealott’s Hill International Research Centre, Bracknell, Berkshire RG42 6EY, UK

**Keywords:** 14-membered resorcylic acid lactones, *Mycobacterium marinum*, anti-*Mycobacterium marinum*, ketal groups, diastereoisomers

## Abstract

The incidence of *Mycobacterium marinum* infection is on the rise; however, the existing drug treatment cycle is lengthy and often requires multi-drug combination. Therefore, there is a need to develop new and effective anti-*M. marinum* drugs. Cochliomycin A, a 14-membered resorcylic acid lactone with an acetonide group at C-5′ and C-6′, exhibits a wide range of antimicrobial, antimalarial, and antifouling activities. To further explore the effect of this structural change at C-5′ and C-6′ on this compound’s activity, we synthesized a series of compounds with a structure similar to that of cochliomycin A, bearing ketal groups at C-5′ and C-6′. The *R*/*S* configuration of the diastereoisomer at C-13′ was further determined through an NOE correlation analysis of CH_3_ or CH_2_ at the derivative C-13′ position and the H-5′ and H-6′ by means of a 1D NOE experiment. Further comparative ^1^H NMR analysis of diastereoisomers showed the difference in the chemical shift (*δ*) value of the diastereoisomers. The synthetic compounds were screened for their anti-microbial activities in vitro. Compounds **15**–**24** and **28**–**35** demonstrated promising activity against *M. marinum*, with MIC_90_ values ranging from 70 to 90 μM, closely approaching the MIC_90_ of isoniazid. The preliminary structure–activity relationships showed that the ketal groups with aromatic rings at C-5′ and C-6′ could enhance the inhibition of *M. marinum*. Further study demonstrated that compounds **23**, **24**, **29**, and **30** had significant inhibitory effects on *M. marinum* and addictive effects with isoniazid and rifampicin. Its effective properties make it an important clue for future drug development toward combatting *M. marinum* resistance.

## 1. Introduction

*Mycobacterium marinum* (*M. marinum*), a nontuberculous mycobacterium (NTM), is one of the major contributors to extrapulmonary mycobacterial infections [1,2]. *M. marinum* infects human skin and soft tissues, leading to the development of a single papulonodular, verrucose, or ulcerated granulomatous lesion at the infected site [3,4,5,6,7]. Notably, such a local infection may spread to the tendon sheaths or joints [8]. In recent years, the incidence of *M. marinum* infection has shown an increasing trend [9]. In the United States, the incidence of this disease can reach 0.27 infections per 100,000 people, with the highest incidence observed among individuals who come into contact with fish and fish containers [2]. For *M. marinum* infection, drugs such as rifambutin, ethambutol, clarithromycin, and others are often used in combinations of two or three, and the duration of medication is generally 2 weeks to 18 months [10,11,12]. However, the existing drug treatments are less than satisfactory. The treatment period for *M. marinum* is lengthy, and multi-drug combinations are required. In addition, there is no standard for the treatment of *M. marinum* infection in terms of drug selection, dose, and concentration [13]. These problems have led to an urgent need to optimize treatments and develop novel and effective anti-*M. marinum* drugs.

Natural products are of outstanding significance in drug development, and numerous drugs or lead compounds are inspired by them [14,15]. In the decades from 1981 to 2019, more than 50% of small-molecule drugs developed globally were influenced by natural products [16,17]. Microorganisms are capable of producing secondary metabolites with novel structures and significant biological activity, and these metabolites have served as a source of inspiration for the development of many new drugs [18,19,20,21,22]. The natural products derived from marine microorganisms possess many unique properties and important value, distinct from terrestrial natural products available on land. Marine natural products exhibit rich diversity, extensive medicinal value, and biological activities [23,24].

Marine fungi have been shown to have complex and varied structures and can produce secondary metabolites with various biological activities that have considerable synthetic value and great significance. They hold significant promise in the research and development of new pharmaceuticals [25,26]. Our laboratory has also focused further attention and research on marine-derived fungi, with the expectation of finding and developing more natural products and derivatives with multifarious activities [27,28].

The 14-membered resorcylic acid lactones (RALs) are polyketides characterized by a 14-membered macrocyclic ring fused to a resorcylic acid residue, and they are classified as natural products [29,30]. In our previous research, we isolated a series of 14-membered RALs, including cochliomycins A–G, 5-Bromozeaenol, and 3, 5-Dibromozeaenol, from the marine-derived fungus *Cochliobolus lunatus* [29,31,32,33]. The 14-membered RALs have various biological activities such as antibacterial, antifouling, antimalarial, and antiviral activity [20,31,34,35,36,37].

**Figure 1:**
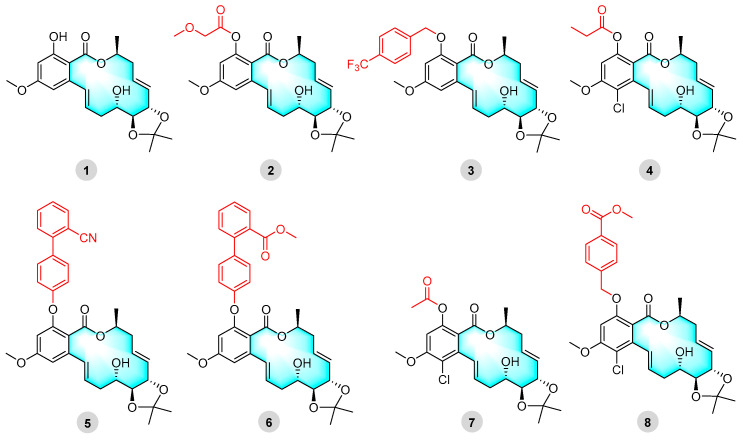
Cochliomycin A (**1**) and its derivatives (**2**–**8**) with strong antialgal and antiplasmodial activities or antibacterial activity.

Cochliomycin A, a natural product of 14-membered RALs with an acetonylic group, can significantly reduce barnacle settlement at a concentration of 1.2 μg/mL [35]. In addition, it has the advantage of exhibiting low toxicity and is an environmentally effective antifouling compound [35]. In our previous study, the cochliomycin A derivative **2** showed strong selective algal inhibitory activity [20]. In particular, the selective inhibition of the diatoms of *Navicula laevissima* and *Navicula exigua* was close to that of SeaNine 211 [20]. Derivatives **5** and **6** showed strong antimalarial activity and non-toxicity when used against *Plasmodium falciparum* [20]. Furthermore, cochliomycin A derivatives also exhibited anti-*M. marinum* activity (Figure 1) [33,36].

Cochliomycin A and its derivatives with acetonide or deuterium acetonyl groups at C-5′ and C-6′ have potent antimalarial and antifouling activity [20]. It is thought that increasing the number of ketal groups at C-5′ and C-6′ would enhance the activity of cochliomycin A derivatives. In this study, we synthesized new compounds, **10**–**37**, which are a series of marine-derived 14-membered RALs with ketal groups. The aim was to obtain 14-membered RAL derivatives with novel structures and evaluate their structure–activity relationships. The synthetic derivatives were screened for in vitro activity against *M. marinum* and five other bacteria and fungi. The activity-screening results showed that derivatives **15**–**24** and **28**–**35** exhibited selective inhibition against *M. marinum*.

## 2. Results and Discussion

### 2.1. Chemistry

**Scheme 1:**
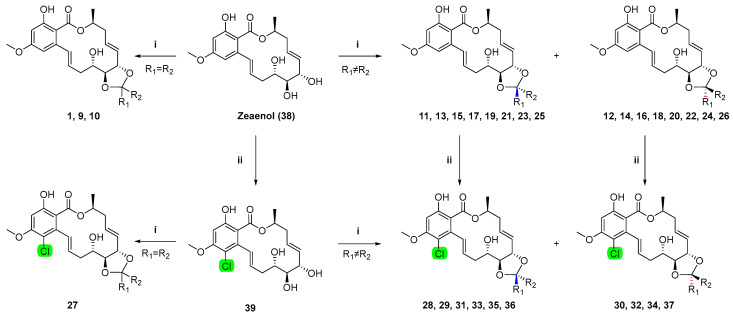
The synthetic route. i *p*-TsOH, K_2_CO_3_, R_1_COR_2_, 25 °C, 2–4 h; ii SO_2_Cl_2_, 0 °C.

The crude extracts obtained from *Cochliobolus lunatus* (CHNSCLM-0009) were separated via silica gel column chromatography. After further recrystallization, about 5.4 g of zeaenol (**38**) was obtained [20]. Using zeaenol (**38**) as the initial raw material, derivatives **10**–**37** with novel ketal groups were semi-synthesized through either a one-step reaction or a two-step chemical reaction (Scheme 1).

**Figure 2:**
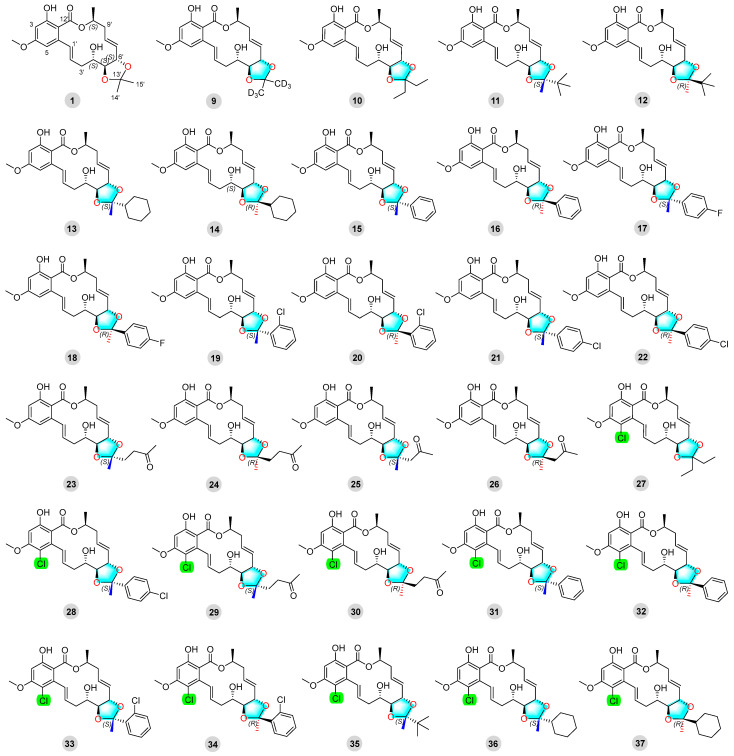
Cochliomycin A (**1**) and its derivatives (**9**–**37**).

Twenty-eight newly synthesized compounds, **10**–**37**, with ketal groups are shown in Figure 2.

**Figure 3:**
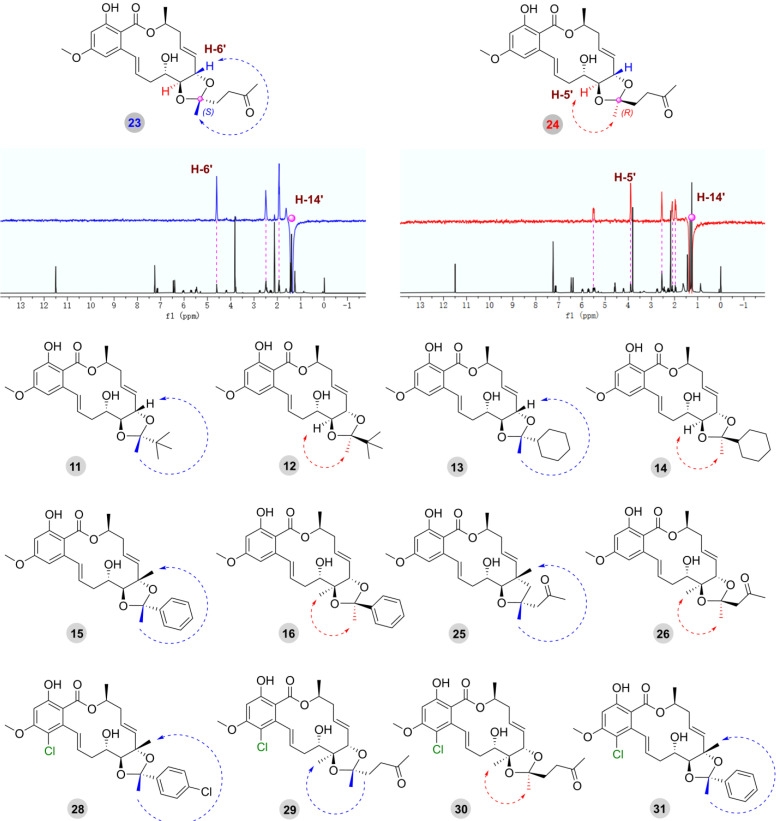
NOE analysis of compounds **23** and **24** and key NOE correlations of other compounds containing ketal groups.

It is worth noting that the *R*/*S* configuration of the C-13′ position was the key point for structural identification. The final determination of the *R*/*S* configuration was achieved by analyzing the correlation between the CH_3_ or CH_2_ at the C-13′ position and the H-5′ and H-6′ of the adjacent chiral center (Figure 3). Compounds **23** and **24** were used to illustrate the detailed structural determination by irradiating H-14′ (*δ*_H_ 1.40) in compound **23**, which resulted in an enhancement of the signal for H-6′ (*δ*_H_ 4.60), suggesting that H-14′ and H-6′ should be placed on the same side of the ring. Conversely, irradiating H-14′ (*δ*_H_ 1.32) in compound **24** enhanced the signal of H-5′ (*δ*_H_ 3.90), indicating that H-14′ and H-5′ should be placed on the same side of the ring. Therefore, the absolute configuration at the C-13′ position of compound **23** was determined to be 13′*S*, and the absolute configuration at C-13′ position of compound **24** was determined to be 13′*R*. The absolute configuration of the C-13′ position of the other derivatives (**11**−**16**, **25**, **26**, and **28**−**31**) was confirmed through analysis of the same experiments (Figure 3, Supplementary Figures S1–S98).

**Figure 4:**
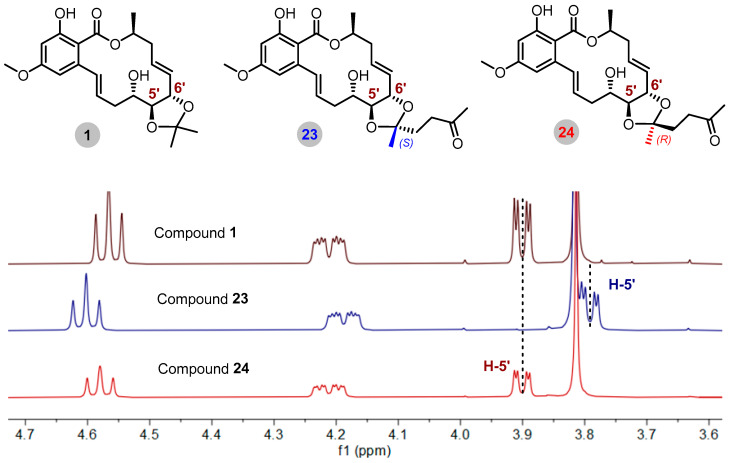
Stacking of partial ^1^H NMR spectra of diastereomeric derivatives (with **23** and **24** as examples).

Meanwhile, the absolute configuration at the C-13′ position was also distinguished by changes in the corresponding chemical shift (*δ*) value of H-5′. In the ^1^H NMR spectrum, the chemical shifts of H-5′ signals were more affected by the shielding effect compared with the data on compound **1** when the absolute configuration of C-13′ position is *S*. The chemical shift of H-5′ in the *S* configuration for derivative **23** shifted from 3.90 to 3.79 ppm. On the contrary, there was no difference regarding the chemical shift of H-5′ in the *R* configuration for derivative **24** compared with the data of compound **1** (*δ*_H_ 3.90 in **24**, *δ*_H_ 3.90 in **1**) (Figure 4). The key ^1^H NMR data (δ) for diastereoisomers are presented in Table 1, and key ^13^C NMR data (δ) for diastereoisomers are presented in Table S2.

### 2.2. Evaluation of Biological Activity

#### 2.2.1. Anti-*M. marinum* and Other-Antimicrobial Activity

The treatment period for *M. marinum* is long and requires multi-drug combination therapy. However, there is a lack of criteria for the optimal antimicrobial regimen and duration of treatment after *M. marinum* infection [13,38]. In this study, we assessed the antibacterial and antifungal activities of 30 14-membered RAL derivatives. We found that most of the compounds demonstrated significant antibacterial activity, as shown in Table 2. The remaining derivatives (MIC_90_ > 200 µM) were categorized as less effective, as shown in Table 2. Derivatives **15**–**24** and **28**–**35** exhibited promising activity against *M. marinum* compared to the positive isoniazid with an MIC_90_ of 40 μM. It is worth noting that most of the compounds displayed a degree of antibacterial selectivity.

**Figure 5:**
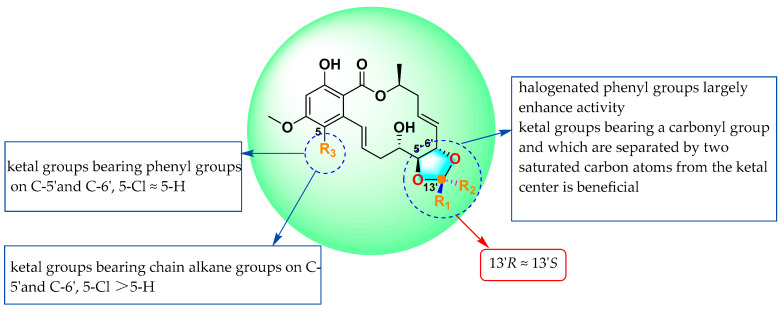
Summary of the structure–activity relationships.

By summarizing the MIC_90_ data and structures of the above 30 compounds, we obtained preliminary insights into structure–activity relationships (SARs) (Figure 5): (1) through a comparison of compounds **11** and **35**, it was found that the 14-membered RALs had no obvious activity after the introduction of ketal groups bearing chain alkane groups, but the activity levels increased significantly after the further introduction of chlorine atoms at C-5; (2) by comparing the MICs of **23**, **24**, **25**, and **26**, it was observed that the introduction of ketal groups that bear a carbonyl group and are separated by two saturated carbon atoms from the ketal center at C-5′ and C-6′ significantly enhanced anti-*M. marinum* activity; (3) by comparing the MICs of compounds **15**–**22**, it was observed that the introduction of ketal groups bearing phenyl groups or phenyl groups with halogen atoms (F/Cl) at C-5′ and C-6′ largely enhanced activity; and (4) after the introduction of ketal groups bearing phenyl groups at C-5′ and C-6′ and the further introduction of chlorine atoms at C-5, the anti-*M. marinum* activity of the products changed little compared with that before introduction (**15**–**22**, **28**, and **31**–**34**).

#### 2.2.2. Anti-*M. marinum* Effects of Compounds **23**, **24**, **29**, and **30** in Combination with Positive Drugs

**Figure 6:**
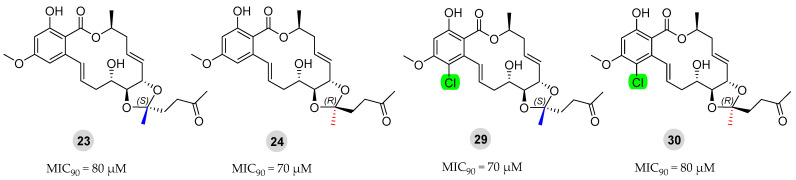
Chemical structures of derivatives **23**, **24**, **29**, and **30** and their anti-*M. marinum* activity.

Currently, the clinical management of *M. marinum* infections typically involves combination therapy, wherein two or three drugs are administered concurrently [33,39]. Compounds **23**, **24**, **29,** and **30** had significant anti-*M. marinum activity,* close to that of the positive drug isoniazid, as shown in Figure 6. Compounds **23**, **24**, **29**, and **30** were evaluated in conjunction with two standard antibiotics (isoniazid and rifampicin) using the checkerboard method for drug sensitivity testing [33]. The MIC_90_ values obtained from these combinations are presented in Table 3 and Table 4.

The findings indicate that all the compounds effectively reduced the required dosages of the standard antibiotics while exhibiting antibacterial activity. Notably, at a concentration of 8.75 μM for compounds **24** and **29**, *M. marinum’*s sensitivity to both rifampicin and isoniazid increased twofold, demonstrating a significant addictive effect. It is important to highlight that following combination therapy, the dosages of both compounds and standard antibiotics decreased; this reduction contributed to mitigating resistance in *M. marinum* to some extent.

## 3. Materials and Methods

### 3.1. General Experimental Procedures

Column chromatography (CC) was performed using silica gel (Qingdao Haiyang Chemical Group Co., Qingdao, China; 200–300 mesh) and Sephadex LH-20 (Amersham Biosciences, Amersham, UK). TLC silicone plate (Yantai Zifu Chemical Group Co., Yantai, China; G60, F-254) was used to meet the needs of thin-layer chromatography analysis. Semi-preparative HPLC was performed using a Waters 1525 system with a semi-preparative C18 column (Amsterdam, The Netherlands; Kromasil, 5 µm, 10 × 250 mm) and a Waters 2996 photodiode array detector with a flow rate of 2.0 mL/min. NMR spectra were recorded using a Bruker Advance NEO 400. Chemical shifts *δ* were measured in ppm, using TMS as the internal standard, and coupling constants (*J*) were measured in Hz. A Micromass Q-TOF mass spectrometer was used to detect HRESIMS spectra.

### 3.2. Fungal Material

The fungal strain *Cochliobolus lunatus* (CHNSCLM-0009) was isolated from a piece of tissue from the inner part of the gorgonian coral *Dichotella gemmacea* (GX-WZ-20080034) collected from the Weizhou coral reef in the South China Sea in September 2008. Through 16S rRNA gene analysis, the fungus was identified as Clostridium selenospora with the access code ZJ2008002. The fungal strain is currently in storage at the Key Laboratory of Marine Drugs, the Ministry of Education of China, School of Medicine and Pharmacy, Ocean University of China, Qingdao, China.

### 3.3. Fermentation, Extraction, and Isolation

*Cochliobolus lunatus* (CHNSCLM-0009) was cultivated under liquid fermentation conditions.

Fermentation was carried out using 500 mL flasks, each filled with 200 mL liquid medium (soluble starch (2 g), NaNO_3_ (1 g), NaOAc (0.2 g), 1% salinity). The flasks were cultivated at 28 °C for 10 days on a rotary shaker at 120 rpm [20,40]. The fermentation liquid in each flask was extracted three or four times with the same volume of EtOAc. The combined EtOAc solution was evaporated until dryness under a vacuum to obtain 19 g crude extract. Ethyl acetate and petroleum ether were selected as eluents, and the crude extracts were separated via silica gel column chromatography (CC). Zeaenol (**38**) was obtained at a 4:1 (*v*/*v*) ratio of petroleum ether/ethyl acetate eluent. After recrystallization with ethyl acetate/petroleum ether/methanol reagent, 5.4 g of zeaenol (**38**) was obtained.

### 3.4. General Synthetic Methods for Compounds ***10**–**37***

Here, we describe in detail the steps of synthesizing the new compounds **10**–**37**. Compounds **10**–**37** were identified using NMR and HRESIMS data, and more details are provided in the Supplementary Data.

#### 3.4.1. General Procedure for the Synthesis of **10**–**26**

Zeaenol (**38**, 30 mg, 82.33 µmol), ketone reagent (2 mL, Supplementary Table S1), and *p*-TsOH (trace) in dry CH_2_Cl_2_ (2 mL) were stirred at 25 °C for 2–4 h. During the reaction, TLC was used to monitor the reaction progress. The reaction solution was extracted with H_2_O and CH_2_Cl_2_, and the organic layer was evaporated until dry to obtain the crude product. Compounds **10**–**14** and **17**–**22** were obtained by separating the crude products on silica gel CC (200–300 mesh) using petroleum ether and ethyl acetate as eluents. After this separation process, we used semipreparative HPLC (65%–85% CH_3_CN-H_2_O) to obtain pure compounds **15**, **16**, and **23**–**26**.

#### 3.4.2. General Procedure for the Synthesis of **28**, **31**, **33**, and **34**

Compound **39** (30 mg, 73.36 µmol), ketone reagent (2 mL, Supplementary Table S1), and *p*-TsOH (trace) in dry CH_2_Cl_2_ (2 mL) were stirred at 25 °C for 2–4 h. During the reaction, TLC was used to monitor the reaction progress. The reaction solution was extracted with H_2_O and CH_2_Cl_2_, and the organic layer was evaporated until dry to obtain the crude product. Compounds **28**, **31**, **33**, and **34** were obtained by separating the crude products on silica gel CC (200–300 mesh) using petroleum ether and ethyl acetate as eluents.

#### 3.4.3. General Procedure for the Synthesis of **27**, **29**, **30**, **32**, **35**, and **37**

Compound **10** (30 mg, 69.41 µmol) and SO_2_Cl_2_ (10 μL) were dissolved in CH_2_Cl_2_ (2 mL). The SO_2_Cl_2_ mixture was slowly dripped into the reaction system under ice-bath conditions and stirred for 1–3 h. The reaction was detected via TLC and stopped with ice water. The reaction solution was extracted with H_2_O and CH_2_Cl_2_, and the organic layer was evaporated until dry to obtain the crude product. Compound **27** was obtained by separating the crude products on silica gel CC (200–300 mesh) using petroleum ether and ethyl acetate as eluents. The synthesis and separation of compounds **29**, **30**, **32**, **35**, and **37** were the same as those noted above. Notably, in obtaining compounds **29** and **30**, we further used semipreparative HPLC (65–85% CH_3_CN-H_2_O).

#### 3.4.4. Characterization Data of Compounds **10**–**37**

The planar structures of the new compounds **10**–**37** were determined using NMR data and HRESIMS spectrum, and the data are as follows. The 1D-NOE was used to determine its spatial structure, and other details were included in the Supplementary Data.

Compound **10**: white, solid; yield, 93.0%; ^1^H NMR (400 MHz, CDCl_3_) *δ* 11.50 (1H, s), 7.16 (1H, dd, *J* = 15.4, 2.4 Hz), 6.47 (1H, d, *J* = 2.6 Hz), 6.40 (1H, d, *J* = 2.6 Hz), 6.01 (1H, m), 5.72 (1H, ddd, *J* = 15.4, 10.5, 3.1 Hz), 5.54–5.43 (2H, overlapped), 4.56 (t, *J* = 8.4 Hz, 1H), 4.25 (m, 1H), 3.85 (dd, *J* = 8.4, 2.4 Hz, 1H), 3.82 (s, 3H), 2.76 (m, 1H), 2.58–2.38 (overlapped, 3H), 2.30 (m, 1H), 1.69 (qd, *J* = 7.5, 1.9 Hz, 2H), 1.61 (d, *J* = 7.5 Hz, 2H), 1.44 (3H, d, *J* = 6.4 Hz), 0.94 (3H, t, *J* = 7.5 Hz), 0.87 (3H, t, *J* = 7.5 Hz). ^13^C NMR (100 MHz, CDCl_3_) *δ* 170.9 (C), 164.9 (C), 164.1 (C), 142.3 (C), 134.2 (CH), 132. (CH), 129.7 (CH), 126.5 (CH), 112.3 (C), 107.3 (CH), 104.6 (C), 100.2 (CH), 81.6 (CH), 75.5 (CH), 70.7 (CH), 69.2 (CH), 55.6 (OCH_3_), 38.1 (CH_2_), 36.1 (CH_2_), 30.5 (CH_2_), 30.0 (CH_2_), 19.3 (CH_3_), 8.4 (CH_3_), 8.2 (CH_3_). HRESIMS *m*/*z* 433.2221 [M + H]^+^ (calcd for C_24_H_33_O_7_^+^, 433.2221).

Compound **11**: white, solid; yield, 79.4%; ^1^H NMR (400 MHz, CDCl_3_) *δ* 11.48 (1H, s), 7.14 (1H, dd, *J* = 15.4, 2.4 Hz), 6.45 (1H, d, *J* = 2.6 Hz), 6.39 (1H, d, *J* = 2.6 Hz), 6.05 (1H, m), 5.68 (1H, ddd, *J* = 15.4, 10.3, 3.0 Hz), 5.53–5.38 (2H, overlapped), 4.62 (1H, t, *J* = 8.5 Hz), 4.19 (1H, m), 3.81 (3H, s), 3.68 (1H, dd, *J* = 8.5, 2.5 Hz), 2.75 (1H, m), 2.58–2.39 (3H, overlapped), 2.32 (1H, m), 1.44 (3H, d, *J* = 6.4 Hz), 1.35 (3H, s), 0.93 (9H, s). ^13^C NMR (100 MHz, CDCl_3_) *δ* 170.9 (C), 164.8 (C), 164.1 (C), 142.5 (C), 134.2 (CH), 131.2 (CH), 130.0 (CH), 126.5 (CH), 113.9 (C), 107.4 (CH), 104.6 (C), 100.2 (CH), 82.9 (CH), 75.1 (CH), 70.7 (CH), 68.7 (CH), 55.6 (OCH_3_), 39.3 (CH_2_), 38.2 (CH_2_), 35.7 (C), 25.4 (CH_3_ × 3), 21.1 (CH_3_), 19.4 (CH_3_). HRESIMS *m*/*z* 447.2367 [M + H]^+^ (calcd for C_25_H_35_O_7_^+^, 447.2377).

Compound **12**: white, solid; yield, 24.1%; ^1^H NMR (400 MHz, CDCl_3_) *δ* 11.49 (1H, s), 7.16 (1H, dd, *J* = 15.4, 2.4 Hz), 6.48 (1H, d, *J* = 2.6 Hz), 6.40 (1H, d, *J* = 2.6 Hz), 5.98 (1H, m), 5.73 (1H, ddd, *J* = 15.4, 10.4, 3.0 Hz), 5.55–5.42 (2H, overlapped), 4.49 (1H, t, *J* = 8.5 Hz), 4.32 (1H, m), 3.93 (1H, dd, *J* = 8.5, 2.3 Hz), 3.82 (3H, s), 2.75 (1H, m), 2.57–2.38 (3H, overlapped), 2.30 (1H, m), 1.44 (3H, d, *J* = 6.4 Hz), 1.28 (3H, s), 1.00 (9H, s). ^13^C NMR (100 MHz, CDCl_3_) *δ* 170.9 (C), 164.9 (C), 164.1 (C), 142.2 (C), 134.2 (CH), 133.7 (CH), 129.3 (CH), 126.4 (CH), 113.5 (C), 107.3 (CH), 104.6 (C), 100.2 (CH), 80.8 (CH), 75.9 (CH), 70.6 (CH), 69.7 (CH), 55.6 (OCH_3_), 38.8 (CH_2_), 38.1 (CH_2_), 36.3 (C), 25.6 (CH_3_ × 3), 20.6 (CH_3_), 19.3 (CH_3_). HRESIMS *m*/*z* 447.2372 [M + H]^+^ (calcd for C_25_H_35_O_7_^+^, 447.2377).

Compound **13**: white, solid; yield, 69.7%; ^1^H NMR (400 MHz, CDCl_3_) *δ* 11.50 (1H, s), 7.15 (1H, dd, *J* = 15.4, 2.4 Hz), 6.46 (1H, d, *J* = 2.6 Hz), 6.40 (1H, d, *J* = 2.6 Hz), 6.02 (1H, m), 5.70 (1H, ddd, *J* = 15.4, 10.4, 3.1 Hz), 5.53–5.39 (2H, overlapped), 4.58 (1H, t, *J* = 8.4 Hz), 4.20 (1H, m), 3.81 (3H, s), 3.74 (1H, dd, *J* = 8.4, 2.4 Hz), 2.75 (1H, m), 2.64–2.38 (3H, overlapped), 2.30 (1H, m), 1.83–1.60 (6H, overlapped), 1.44 (3H, d, *J* = 6.4 Hz), 1.32 (3H, s), 1.22–0.96 (5H, overlapped). ^13^C NMR (100 MHz, CDCl_3_) *δ* 170.9 (C), 164.9 (C), 164.1 (C), 142.4 (C), 134.2 (CH), 132.0 (CH), 129.8 (CH), 126.6 (CH), 112.0 (C), 107.3 (CH), 104.6 (C), 100.2 (CH), 82.1 (CH), 75.0 (CH), 70.7 (CH), 68.8 (CH), 55.6 (OCH_3_), 47.3 (CH_2_), 38.1 (CH_2_), 35.9 (C), 27.7 (CH), 27.5 (CH), 26.4 (CH), 26.4 (CH), 26.3 (CH), 22.6 (CH_3_), 19.4 (CH_3_). HRESIMS *m*/*z* 473.2522 [M + H]^+^ (calcd for C_27_H_37_O_7_^+^, 473.2534).

Compound **14**: white, solid; yield, 33.2%; ^1^H NMR (400 MHz, CDCl_3_) *δ* 11.50 (1H, s), 7.16 (1H, dd, *J* = 15.4, 2.3 Hz), 6.47 (1H, d, *J* = 2.6 Hz), 6.40 (1H, d, *J* = 2.6 Hz), 5.98 (1H, m), 5.73 (1H, ddd, *J* = 15.4, 10.5, 3.1 Hz), 5.56–5.40 (2H, overlapped), 4.49 (1H, t, *J* = 8.4 Hz), 4.27 (1H, m), 3.91 (1H, dd, *J* = 8.4, 2.3 Hz), 3.81 (3H, s), 2.75 (1H, m), 2.56–2.37 (3H, overlapped), 2.29 (1H, m), 1.89–1.65 (6H, overlapped), 1.44 (3H, d, *J* = 6.4 Hz), 1.25 (3H, s), 1.22–1.01 (5H, overlapped). ^13^C NMR (101 MHz, CDCl_3_) *δ* 170.9 (C), 164.9 (C), 164.1 (C), 142.2 (C), 134.2 (CH), 133.4 (CH), 129.5 (CH), 126.5 (CH), 111.5 (C), 107.3 (CH), 104.6 (C), 100.2 (CH), 80.9 (CH), 75.5 (CH), 70.7 (CH), 69.4 (CH), 55.6 (OCH_3_), 47.3 (CH_2_), 38.0 (CH_2_), 36.2 (CH), 27.7 (CH_2_), 27.6 (CH_2_), 26.4 (CH_2_×2), 26.3 (CH_2_), 22.6 (CH_3_), 19.3 (CH_3_). HRESIMS *m*/*z* 473.2523 [M + H]^+^ (calcd for C_27_H_37_O_7_^+^, 473.2534).

Compound **15**: white, solid; yield, 63.7%; ^1^H NMR (400 MHz, CDCl_3_) *δ* 11.49 (1H, s), 7.45–7.42 (2H, overlapped), 7.23–7.27 (3H, overlapped), 7.02 (1H, dd, *J* = 15.4, 2.4 Hz), 6.43 (1H, d, *J* = 2.6 Hz), 6.35 (1H, d, *J* = 2.6 Hz), 5.94 (1H, m), 5.68 (1H, ddd, *J* = 15.4, 10.5, 3.0 Hz), 5.42 (1H, m), 5.11 (1H, m), 4.70 (1H, dd, *J* = 9.5, 7.3 Hz), 4.32 (1H, m), 3.79 (3H, s), 3.73 (1H, dd, *J* = 7.3, 2.2 Hz), 2.79 (1H, m), 2.68 (1H, s), 2.39–2.19 (3H, overlapped), 1.65 (3H, s), 1.41 (3H, d, *J* = 6.5 Hz). ^13^C NMR (125 MHz, CDCl_3_) *δ* 170.6 (C), 164.9 (C), 164.0 (C), 144.4 (C), 141.9 (C), 134.0 (CH), 133.5 (CH), 129.0 (CH), 128.4 (CH × 2), 128.1 (CH), 125.7 (CH), 125.4 (CH × 2), 109.2 (C), 107.1 (CH), 104.4 (C), 100.2 (CH), 81.6 (CH), 76.5 (CH), 70.3 (CH), 69.1 (CH), 55.6 (OCH_3_), 37.8 (CH_2_), 36.3 (CH_2_), 29.1 (CH_3_), 19.0 (CH_3_). HRESIMS *m*/*z* 467.2081 [M + H]^+^ (calcd for C_27_H_29_O_7_^+^, 467.2064).

Compound **16**: white, solid; yield, 43.1%; ^1^H NMR (400 MHz, CDCl_3_) *δ* 11.50 (1H, s), 7.51–7.48 (2H, overlapped), 7.40–7.36 (2H, overlapped), 7.30 (1H, m), 7.15 (1H, dd, *J* = 15.4, 2.3 Hz), 6.46 (1H, d, *J* = 2.6 Hz), 6.40 (1H, d, *J* = 2.6 Hz), 6.08 (1H, m), 5.71 (1H, ddd, *J* = 15.4, 10.6, 3.1 Hz), 5.62 (1H, m), 5.46 (1H, m), 4.59 (1H, t, *J* = 8.3 Hz), 4.22 (1H, m), 4.03 (1H, dd, *J* = 8.3, 2.2 Hz), 3.82 (3H, s), 2.65 (1H, m), 2.61–2.42 (2H, overlapped), 2.19 (1H, m), 1.90 (1H, m), 1.65 (3H, s), 1.45 (3H, d, *J* = 6.4 Hz). ^13^C NMR (125 MHz, CDCl_3_) *δ* 170.9 (C), 164.9 (C), 164.1 (C), 144.6 (C), 142.3 (C), 134.1 (CH), 131.8 (CH), 130.5 (CH), 128.9 (CH × 2), 128.3 (CH), 126.8 (CH), 124.6 (CH × 2), 109.1 (C), 107.4 (CH), 104.5 (C), 100.2 (CH), 82.9 (CH), 75.7 (CH), 70.7 (CH), 69.4 (CH), 55.6 (OCH_3_), 38.1 (CH_2_), 35.9 (CH_2_), 29.2 (CH_3_), 19.4 (CH_3_). HRESIMS *m*/*z* 467. 2057 [M + H]^+^ (calcd for C_27_H_31_O_7_^+^, 467.2064).

Compound **17**: white, solid; yield, 64.8%; ^1^H NMR (400 MHz, CDCl_3_) *δ* 11.49 (1H, s), 7.43–7.39 (2H, overlapped), 7.03 (1H, dd, *J* = 15.4, 2.4 Hz), 7.01– 6.95 (2H, overlapped), 6.44 (1H, d, *J* = 2.6 Hz), 6.35 (1H, d, *J* = 2.6 Hz), 5.95 (1H, m), 5.68 (1H, ddd, *J* = 15.4, 10.5, 3.0 Hz), 5.43 (1H, m), 5.10 (1H, m), 4.69 (1H, dd, *J* = 9.5, 7.3 Hz), 4.31 (1H, m), 3.79 (3H, s), 3.71 (1H, dd, *J* = 7.3, 2.2 Hz), 2.79 (1H, m), 2.67 (1H, s), 2.38–2.35 (2H, overlapped), 2.25 (1H, m), 1.63 (3H, s), 1.41 (3H, d, *J* = 6.5 Hz). ^13^C NMR (100 MHz, CDCl_3_) *δ* 170.6 (C), 165.0 (C), 164.0 (C), 162.6 (C, d, *J* = 244.1 Hz), 141.8 (C), 140.3 (C, d, *J* = 3.2 Hz), 134.0 (CH), 133.3 (CH), 129.2 (CH), 127.3 (CH × 2, d, *J* = 8.1 Hz), 125.6 (CH), 115.2 (CH × 2, d, *J* = 21.5 Hz), 108.8 (C), 107.2 (CH), 104.4 (C), 100.2 (CH), 81.7 (CH), 76.5 (CH), 70.2 (CH), 69.0 (CH), 55.5 (OCH_3_), 37.8 (CH_2_), 36.3 (CH_2_), 29.2 (CH_3_), 19.0 (CH_3_). HRESIMS *m*/*z* 485.1974 [M + H]^+^ (calcd for C_27_H_31_O_7_F^+^, 485.1970).

Compound **18**: white, solid; yield, 41.7%; ^1^H NMR (400 MHz, CDCl_3_) *δ* 11.49 (1H, s), 7.43–7.39 (2H, overlapped), 7.16 (1H, dd, *J* = 15.4, 2.3 Hz), 7.01–6.95 (2H, overlapped), 6.46 (1H, d, *J* = 2.6 Hz), 6.40 (1H, d, *J* = 2.6 Hz), 6.07 (1H, m), 5.71 (1H, ddd, *J* = 15.4, 10.5, 3.1 Hz), 5.60 (1H, m), 5.47 (1H, m), 4.58 (1H, t, *J* = 8.4 Hz), 4.22 (1H, m), 4.02 (1H, dd, *J* = 8.4, 2.2 Hz), 3.82 (3H, s), 2.67 (1H, m), 2.61–2.44 (2H, overlapped), 2.20 (1H, m), 1.90 (1H, s), 1.62 (3H, s), 1.45 (3H, d, *J* = 6.4 Hz). ^13^C NMR (100 MHz, CDCl_3_) *δ* 170.9 (C), 164.9 (C), 164.1 (C), 162.6 (C, d, *J* = 244.9 Hz), 142.3 (C), 140.4 (C, d, *J* = 3.5 Hz), 134.2 (CH), 131.6 (CH), 130.6 (CH), 126.7 (CH), 126.5 (CH × 2, d, *J* = 8.4 Hz), 115.7 (CH × 2, d, *J* = 21.5 Hz), 108.7 (C), 107.4 (CH), 104.5 (C), 100.2 (CH), 82.9 (CH), 75.8 (CH), 70.7 (CH), 69.2 (CH), 55.6 (OCH_3_), 38.0 (CH_2_), 35.9 (CH_2_), 29.2 (CH_3_), 19.4 (CH_3_). HRESIMS *m*/*z* 485.1973 [M + H]^+^ (calcd for C_17_H_30_O_7_F^+^, 485.1970).

Compound **19**: white, solid; yield, 65.4%; ^1^H NMR (400 MHz, CDCl_3_) *δ* 11.48 (1H, s), 7.60 (1H, m), 7.34 (1H, m), 7.22–7.18 (2H, overlapped), 7.04 (1H, dd, *J* = 15.4, 2.4 Hz), 6.45 (1H, d, *J* = 2.6 Hz), 6.35 (1H, d, *J* = 2.6 Hz), 5.95 (1H, m), 5.71 (1H, ddd, *J* = 15.4, 10.5, 3.0 Hz), 5.42 (1H, m), 5.10 (1H, m), 4.72 (1H, dd, *J* = 9.4, 7.3 Hz), 4.34 (1H, m), 3.79 (3H, s), 3.73 (1H, dd, *J* = 7.3, 2.2 Hz), 2.80 (1H, m), 2.68 (1H, s), 2.40–2.33 (2H, overlapped), 2.26 (1H, m), 1.78 (3H, s), 1.41 (3H, d, *J* = 6.5 Hz). ^13^C NMR (100 MHz, CDCl_3_) *δ* 170.7 (C), 164.9 (C), 164.0 (C), 141.9 (C), 140.6 (C), 134.0 (C), 132.9 (CH), 132.0 (CH), 131.6 (CH), 129.6 (CH), 129.3 (CH), 127.9 (CH), 126.8 (CH), 125.9 (CH), 108.7 (CH), 107.2 (CH), 104.4 (C), 100.2 (CH), 81.6 (CH), 76.3 (CH), 70.3 (CH), 69.0 (CH), 55.6 (OCH_3_), 37.8 (CH_2_), 36.4 (CH_2_), 26.5 (CH_3_), 19.0 (CH_3_). HRESIMS *m*/*z* 501.1692 [M + H]^+^ (calcd for C_27_H_30_O_7_Cl^+^, 501.1675).

Compound **20**: white, solid; yield, 33.8%; ^1^H NMR (400 MHz, CDCl_3_) *δ* 11.49 (1H, s), 7.65 (1H, dd, *J* = 7.5, 2.0 Hz), 7.40 (1H, dd, *J* = 7.5, 1.7 Hz), 7.29 (1H, dd, *J* = 7.4, 1.7 Hz), 7.24 (1H, dd, *J* = 7.4, 2.1 Hz), 7.14 (1H, dd, *J* = 15.3, 2.3 Hz), 6.45 (1H, d, *J* = 2.6 Hz), 6.40 (1H, d, *J* = 2.6 Hz), 6.10 (1H, m), 5.75–5.56 (2H, overlapped), 5.48 (1H, m), 4.55 (1H, t, *J* = 8.4 Hz), 4.26 (1H, m), 4.02 (1H, dd, *J* = 8.4, 2.3 Hz), 3.82 (3H, s), 2.65 (1H, m), 2.60–2.45 (2H, overlapped), 2.19 (1H, m), 1.93 (1H, s), 1.77 (3H, s), 1.45 (3H, d, *J* = 6.4 Hz). ^13^C NMR (100 MHz, CDCl_3_) *δ* 170.9 (C), 164.9 (C), 164.1 (C), 142.3 (C), 140.9 (C), 134.2 (C), 131.8 (CH), 131.4 (CH), 131.3 (CH), 130.9 (CH), 129.7 (CH), 127.4 (CH), 127.0 (CH), 126.5 (CH), 108.8 (C), 107.4 (CH), 104.5 (C), 100.2 (CH), 82.9 (CH), 75.5 (CH), 70.6 (CH), 69.2 (CH), 55.6 (OCH_3_), 38.1 (CH_2_), 35.8 (CH_2_), 27.0 (CH_3_), 19.4 (CH_3_). HRESIMS *m*/*z* 501.1672 [M + H]^+^ (calcd for C_27_H_30_OCl^+^, 501.1675).

Compound **21**: white, solid; yield, 56.6%; ^1^H NMR (400 MHz, CDCl_3_) *δ* 11.49 (1H, s), 7.39–7.36 (2H, overlapped), 7.29–7.26 (2H, overlapped), 7.03 (1H, dd, *J* = 15.4, 2.4 Hz), 6.43 (1H, d, *J* = 2.6 Hz), 6.35 (1H, d, *J* = 2.6 Hz),5.95 (1H, m), 5.68 (1H, ddd, *J* = 15.4, 10.5, 3.0 Hz), 5.44 (1H, m), 5.09 (1H, dd, *J* = 15.2, 9.4, 1.3 Hz), 4.69 (1H, dd, *J* = 9.4, 7.3 Hz), 4.30 (1H, m), 3.79 (3H, s), 3.69 (1H, dd, *J* = 7.3, 2.2 Hz), 2.79 (1H, m), 2.63 (1H, s), 2.38–2.19 (3H, overlapped), 1.62 (3H, s), 1.41 (3H, d, *J* = 6.5 Hz). ^13^C NMR (100 MHz, CDCl_3_) *δ* 170.6 (C), 165.0 (C), 164.0 (C), 143.0 (C), 141.8 (C), 134.1 (C), 134.0 (CH), 133.3 (CH), 129.3 (CH), 128.6 (CH × 2), 127.0 (CH × 2), 125.6 (CH), 108.7 (C), 107.2 (CH), 104.4 (C), 100.2 (CH), 81.7 (CH), 76.5 (CH), 70.2 (CH), 69.0 (CH), 55.6 (CH), 37.8 (OCH_3_), 36.3 (CH_2_), 29.0 (CH_3_), 19.0 (CH_3_). HRESIMS *m*/*z* 501.1685 [M + H]^+^ (calcd for C_27_H_30_O_7_Cl^+^, 501.1675).

Compound **22**: white, solid; yield, 32.3%; ^1^H NMR (400 MHz, CDCl_3_) *δ* 11.49 (1H, s), 7.45–7.42 (2H, overlapped), 7.36–7.33 (2H, overlapped), 7.16 (1H, dd, *J* = 15.4, 2.3 Hz), 6.46 (1H, d, *J* = 2.6 Hz), 6.40 (1H, d, *J* = 2.5 Hz), 6.07 (1H, m), 5.71 (1H, ddd, *J* = 15.4, 10.5, 3.1 Hz), 5.60 (1H, m), 5.46 (1H, m), 4.56 (1H, t, *J* = 8.3 Hz), 4.22 (1H, m), 4.02 (1H, dd, *J* = 8.3, 2.2 Hz), 3.82 (3H, s), 2.66 (1H, m), 2.59–2.44 (2H, overlapped), 2.20 (1H, m), 1.88 (1H, d, *J* = 1.5 Hz), 1.61 (3H, s), 1.44 (3H, d, *J* = 6.3 Hz). ^13^C NMR (101 MHz, CDCl_3_) *δ* 170.9 (C), 164.9 (C), 164.1 (C), 143.0 (C), 142.3 (C), 134.2 (C), 134.1 (CH), 131.5 (CH), 130.7 (CH), 129.0 (CH × 2), 126.7 (CH), 126.2 (CH × 2), 108.6 (C), 107.4 (CH), 104.5 (C), 100.2 (CH), 82.9 (CH), 75.9 (CH), 70.7 (CH), 69.2 (CH), 55.6 (OCH_3_), 38.1 (CH_2_), 35.9 (CH_2_), 29.1 (CH_3_ ), 19.5 (CH_3_ ). HRESIMS *m*/*z* 501.1684 [M + H]^+^ (calcd for C_27_H_30_O_7_Cl^+^, 501.1675).

Compound **23**: white, solid; yield, 52.8%; ^1^H NMR (400 MHz, CDCl_3_) *δ* 11.50 (1H, s), 7.15 (1H, dd, *J* = 15.4, 2.3 Hz), 6.46 (1H, d, *J* = 2.6 Hz), 6.40 (1H, d, *J* = 2.6 Hz), 6.03 (1H, m), 5.70 (1H, ddd, *J* = 15.4, 10.4, 3.0 Hz), 5.52–5.42 (2H, overlapped), 4.60 (1H, t, *J* = 8.5 Hz), 4.19 (1H, m), 3.82 (3H, s), 3.79 (1H, dd, *J* = 8.5, 2.4 Hz), 2.76 (1H, m), 2.58–2.38 (5H, overlapped), 2.29 (1H, m), 2.13 (3H, s), 1.93 (2H, t, *J* = 6.4 Hz), 1.44 (3H, d, *J* = 6.4 Hz), 1.40 (3H, s). ^13^C NMR (100 MHz, CDCl_3_) *δ* 208.2 (C), 170.9 (C), 164.9 (C), 164.1 (C), 142.2 (C), 134.3 (CH), 131.8 (CH), 130.3 (CH), 126.3 (CH), 109.4 (C), 107.4 (CH), 104.5 (C), 100.2 (CH), 82.3 (CH), 75.4 (CH), 70.6 (CH), 68.8 (CH), 55.6 (OCH_3_), 38.4 (CH_2_), 38.0 (CH_2_), 36.0 (CH_2_), 33.8 (CH_3_), 30.1 (CH_2_), 25.5 (CH_3_), 19.3 (CH_3_). HRESIMS *m*/*z* 461.2157 [M + H]^+^ (calcd for C_25_H_33_O_8_^+^, 461.2170).

Compound **24**: white, solid; yield, 35.2%; ^1^H NMR (400 MHz, CDCl_3_) *δ* 11.50 (1H, s), 7.15 (1H, dd, *J* = 15.4, 2.3 Hz), 6.47 (1H, d, *J* = 2.6 Hz), 6.39 (1H, d, *J* = 2.6 Hz), 5.99 (1H, m), 5.73 (1H, ddd, *J* = 15.4, 10.6, 3.1 Hz), 5.57–5.41 (2H, overlapped), 4.58 (1H, t, *J* = 8.3 Hz), 4.21 (1H, m), 3.90 (1H, dd, *J* = 8.3, 2.1 Hz), 3.81 (3H, s), 2.75 (1H, m), 2.65–2.35 (5H, overlapped), 2.27 (1H, m), 2.17 (3H, s), 2.11 (1H, m), 1.96 (1H, m), 1.44 (3H, d, *J* = 6.4 Hz), 1.32 (3H, s). ^13^C NMR (100 MHz, CDCl_3_) *δ* 210.2 (C), 170.9 (C), 164.9 (C), 164.1 (C), 142.2 (C), 134.0 (CH), 133.1 (CH), 129.6 (CH), 126.8 (CH), 109.3 (C), 107.3 (CH), 104.5 (C), 100.2 (CH), 81.9 (CH), 77.4 (CH), 70.7 (CH), 69.0 (CH), 55.6 (OCH_3_), 38.5 (CH_2_), 38.0 (CH_2_), 36.6 (CH_2_), 33.7 (CH_3_), 29.8 (CH_2_), 25.8 (CH_3_), 19.3 (CH_3_). HRESIMS *m*/*z* 461.2155 [M + H]^+^ (calcd for C_2_H_33_O_8_^+^, 461.2170).

Compound **25**: white, solid; yield, 54.4%; ^1^H NMR (400 MHz, CDCl_3_) *δ* 11.48 (1H, s), 7.15 (1H, dd, *J* = 15.4, 2.3 Hz), 6.46 (1H, d, *J* = 2.6 Hz), 6.40 (1H, d, *J* = 2.6 Hz), 6.03 (1H, m), 5.70 (1H, ddd, *J* = 15.4, 10.5, 3.0 Hz), 5.55–5.39 (2H, overlapped), 4.62 (1H, t, *J* = 8.4 Hz), 4.23 (1H, m), 3.89 (1H, dd, *J* = 8.4, 2.3 Hz), 3.81 (3H, s), 2.75 (3H, m), 2.57–2.38 (3H, overlapped), 2.29 (1H, m), 2.17 (3H, s), 1.48 (3H, s), 1.44 (3H, d, *J* = 6.4 Hz). ^13^C NMR (100 MHz, CDCl_3_) *δ* 205.6 (C), 170.9 (C), 164.9 (C), 164.1 (C), 142.2 (C), 134.3 (CH), 131.7 (CH), 130.4 (CH), 126.4 (CH), 107.8 (C), 107.4 (CH), 104.5 (C), 100.2 (CH), 82.1 (CH), 75.5 (CH), 70.6 (CH), 68.6 (CH), 55.6 (OCH_3_), 53.4 (CH_2_), 38.0 (CH_2_), 36.0 (CH_2_), 32.0 (CH_3_), 25.8 (CH_3_), 19.3 (CH_3_). HRESIMS *m*/*z* 447.2026 [M + H]^+^ (calcd for C_24_H_31_O_8_^+^, 447.2013).

Compound **26**: white, solid; yield, 36.3%; ^1^H NMR (400 MHz, CDCl_3_) *δ* 11.48 (1H, s), 7.14 (1H, dd, *J* = 15.4, 2.4 Hz), 6.48 (1H, d, *J* = 2.6 Hz), 6.39 (1H, d, *J* = 2.6 Hz), 6.01 (1H, m), 5.76 (1H, ddd, *J* = 15.4, 10.6, 3.1 Hz), 5.54–5.41 (2H, overlapped), 4.62 (1H, t, *J* = 7.6 Hz), 4.26 (1H, m), 3.96 (1H, dd, *J* = 7.6, 2.1 Hz), 3.81 (3H, s), 2.97 (1H, d, *J* = 14.1 Hz), 2.82–2.71 (2H, overlapped), 2.57–2.37 (2H, overlapped), 2.28–2.18 (5H, overlapped), 1.44 (3H, d, *J* = 6.4 Hz), 1.40 (3H, s). ^13^C NMR (100 MHz, CDCl_3_) *δ* 207.2 (C), 170.9 (C), 164.9 (C), 164.1 (C), 142.2 (C), 133.9 (CH), 133.0 (CH), 130.0 (CH), 126.8 (CH), 107.7 (C), 107.2 (CH), 104.5 (C), 100.2 (CH), 82.1 (CH), 76.0 (CH), 70.5 (CH), 69.1 (CH), 55.6 (OCH_3_), 51.2 (CH_2_), 37.9 (CH_2_), 36.6 (CH_2_), 32.9 (CH_3_), 27.3 (CH_3_), 19.2 (CH_3_). HRESIMS *m*/*z* 447.1998 [M + H]^+^ (calcd for C_24_H_31_O_8_^+^, 447.2013).

Compound **27**: white, solid; yield, 58.8%; ^1^H NMR (400 MHz, CDCl_3_) *δ* 11.39 (1H, s), 6.63 (1H, dd, *J* = 16.2, 1.3 Hz), 6.47 (1H, s), 6.00 (1H, m), 5.54–5.33 (3H, overlapped), 4.54 (1H, t, *J* = 8.6 Hz), 4.23 (1H, m), 3.91 (3H, s), 3.77 (1H, dd, *J* = 8.6, 2.5 Hz), 2.86 (1H, m), 2.62–2.31 (4H, overlapped), 1.75–1.60 (4H, overlapped), 1.39 (3H, d, *J* = 6.4 Hz), 0.93 (3H, t, *J* = 7.5 Hz), 0.88 (3H, t, *J* = 7.5 Hz). ^13^C NMR (100 MHz, CDCl_3_) *δ* 170.7 (C), 162.5 (C), 160.0 (C), 140.0 (C), 131.1 (CH), 130.7 (CH), 130.5 (CH), 129.0 (CH), 114.1 (C), 112.5 (CH), 106.4 (C), 99.5 (CH), 81.2 (CH), 75.7 (CH), 71.5 (CH), 68.6 (CH), 56.6 (OCH_3_), 37.2 (CH_2_), 34.8 (CH_2_), 30.5 (CH_2_), 30.3 (CH_2_), 19.4 (CH_3_), 8.3 (CH_3_), 8.3 (CH_3_). HRESIMS *m*/*z* 467.1817 [M + H]^+^ (calcd for C_24_H_32_O_7_Cl^+^, 467.1831).

Compound **28**: white, solid; yield, 54.5%; ^1^H NMR (400 MHz, CDCl_3_) *δ* 11.45 (1H, s), 7.40–7.37 (2H, overlapped), 7.29–7.27 (2H, overlapped), 6.56 (1H, d, *J* = 14.8 Hz), 6.42 (1H, s), 5.96 (1H, m), 5.43 (1H, ddd, *J* = 14.8, 8.5, 3.8 Hz), 5.35 (1H, m), 5.17 (1H, m), 4.68 (1H, t, *J* = 8.1 Hz), 4.29 (1H, m), 3.88 (3H, s), 3.62 (1H, dd, *J* = 8.1, 2.3 Hz), 2.90 (1H, m), 2.63 (1H, m), 2.51 (1H, m), 2.42–2.25 (2H, overlapped), 1.62 (3H, s), 1.37 (3H, d, *J* = 6.5 Hz). ^13^C NMR (100 MHz, CDCl_3_) *δ* 170.5 (C), 162.6 (C), 160.0 (C), 143.0 (C), 139.7 (C), 134.0 (C), 131.7 (CH), 130.2 (CH), 130.1 (CH), 129.1 (CH), 128.6 (CH × 2), 126.8 (CH × 2), 114.0 (C), 108.7 (CH), 106.2 (C), 99.6 (CH), 81.1 (CH), 76.7 (CH), 71.3 (CH), 68.4 (CH), 56.6 (OCH_3_), 37.1 (CH_2_), 34.8 (CH_2_), 28.9 (CH_3_), 19.1 (CH_3_). HRESIMS *m*/*z* 535.1283 [M + H]^+^ (calcd for C_27_H_29_O_7_Cl2^+^, 535.1285).

Compound **29**: white, solid; yield, 53.9%; ^1^H NMR (400 MHz, CDCl_3_) *δ* 11.41 (1H, s), 6.62 (1H, d, *J* = 16.1 Hz), 6.47 (1H, s), 6.02 (1H, m), 5.54–5.34 (3H, overlapped), 4.59 (1H, t, *J* = 8.7 Hz), 4.17 (1H, m), 3.91 (3H, s), 3.71 (1H, dd, *J* = 8.7, 2.5 Hz), 2.86 (1H, m), 2.62–2.47 (4H, overlapped), 2.47–2.31 (2H, overlapped), 2.13 (3H, s), 1.95 (2H, t, *J* = 7.4 Hz), 1.40–1.38 (6H, overlapped). ^13^C NMR (125 MHz, CDCl_3_) *δ* 208.3 (C), 170.6 (C), 162.5 (C), 160.0 (C), 139.9 (C), 131.1 (CH), 130.4 (CH), 130.2 (CH), 129.1 (CH), 114.1 (C), 109.5 (CH), 106.4 (C), 99.6 (CH), 81.8 (CH), 75.6 (CH), 71.4 (CH), 68.2 (CH), 56.6 (OCH_3_), 38.4 (CH_2_), 37.2 (CH_2_), 34.7 (CH_2_), 33.9 (CH_3_), 30.1 (CH_2_), 25.6 (CH_3_), 19.4 (CH_3_). HRESIMS *m*/*z* 495.1765 [M + H]^+^ (calcd for C_25_H_32_O_8_Cl^+^, 495.1780).

Compound **30**: white, solid; yield, 32.3%; ^1^H NMR (400 MHz, CDCl_3_) *δ* 11.37 (1H, s), 6.63 (1H, dd, *J* = 16.1, 1.6 Hz), 6.46 (1H, s), 5.98 (1H, m), 5.52 (1H, ddd, *J* = 16.1, 9.1, 3.5 Hz), 5.47–5.34 (2H, overlapped), 4.56 (1H, t, *J* = 8.6 Hz), 4.20 (1H, m), 3.91 (3H, s), 3.81 (1H, dd, *J* = 8.6, 2.2 Hz), 2.87 (1H, m), 2.61–2.51 (4H, overlapped), 2.46–2.28 (2H, overlapped), 2.17 (3H, s), 2.10 (1H, m), 1.96 (1H, m), 1.96 (3H, d, *J* = 6.3 Hz), 1.33 (3H, s). ^13^C NMR (100 MHz, CDCl_3_) *δ* 209.8 (C), 170.7 (C), 162.4 (C), 160.0 (C), 140.0 (C), 131.4 (CH), 131.1 (CH), 130.4 (CH), 128.9 (CH), 114.1 (C), 109.4 (CH), 106.5 (C), 99.5 (CH), 81.4 (CH), 75.9 (CH), 71.4 (CH), 68.5 (CH), 56.6 (OCH_3_), 38.5 (CH_2_), 37.2 (CH_2_), 35.4 (CH_2_), 33.7 (CH_3_), 29.9 (CH_2_), 25.7 (CH_3_), 19.5 (CH_3_). HRESIMS *m*/*z* 495.1761 [M + H]^+^ (calcd for C_25_H_32_O_8_Cl^+^, 495.1780).

Compound **31**: white, solid; yield, 58.5%; ^1^H NMR (400 MHz, CDCl_3_) *δ* 11.44 (1H, s), 7.49–7.41 (2H, overlapped), 7.35–7.27 (3H, overlapped), 6.55 (1H, d, *J* = 17.4 Hz), 6.42 (1H, s), 5.95 (1H, m), 5.43 (1H, ddd, *J* = 16.1, 8.5, 3.8 Hz), 5.33 (1H, m), 5.19 (1H, m), 4.69 (1H, t, *J* = 9.0 Hz), 4.30 (1H, m), 3.88 (3H, s), 3.65 (1H, dd, *J* = 9.0, 2.4 Hz), 2.90 (1H, m), 2.67 (1H, s), 2.50 (1H, m), 2.40–2.27 (2H, overlapped), 1.65 (3H, s), 1.37 (3H, d, *J* = 6.5 Hz). ^13^C NMR (100 MHz, CDCl_3_) *δ* 170.5 (C), 162.6 (C), 160.0 (C), 144.3 (C), 139.8 (C), 131.9 (CH), 130.3 (CH), 130.0 (CH), 129.0 (CH), 128.4 (CH × 2), 128.1 (CH), 125.3 (CH × 2), 114.0 (C), 109.1 (CH), 106.2 (C), 99.5 (CH), 81.0 (CH), 76.6 (CH), 71.3 (CH), 68.4 (CH), 56.5 (OCH_3_), 37.1 (CH_2_), 34.8 (CH_2_), 29.0 (CH_3_), 19.1 (CH_3_). HRESIMS *m*/*z* 501.1664 [M + H]^+^ (calcd for C_27_H_30_O_7_Cl^+^, 501.1675).

Compound **32**: white, solid; yield, 37.6%; ^1^H NMR (400 MHz, CDCl_3_) δ 11.35 (1H, s), 7.49 (2H, d, *J* = 7.5 Hz), 7.38 (2H, t, *J* = 7.5 Hz), 7.31 (1H, d, *J* = 7.2 Hz), 6.62 (1H, dd, *J* = 16.1, 2.4 Hz), 6.47 (1H, s), 6.04 (1H, m), 5.58–5.41 (3H, overlapped), 4.53 (1H, t, *J* = 8.6 Hz), 4.20 (1H, m), 3.94 (1H, dd, *J* = 8.6, 2.2 Hz), 3.91 (3H, s), 2.76 (1H, m), 2.65–2.41 (3H, overlapped), 2.23 (1H, m), 1.65 (3H, s), 1.39 (3H, d, *J* = 6.3 Hz). ^13^C NMR (100 MHz, CDCl_3_) *δ* 170.7 (C), 162.4 (C), 160.0 (C), 144.6 (C), 140.0 (C), 131.3 (CH), 130.9 (CH), 130.0 (CH), 128.9 (CH × 3), 128.2 (CH), 124.5 (CH × 2), 114.1 (C), 109.1 (CH), 106.5 (C), 99.5 (CH), 82.4 (CH), 75.9 (CH), 71.3 (CH), 68.8 (CH), 56.6 (OCH_3_), 37.2 (CH_2_), 34.7 (CH_2_), 29.3 (CH_3_), 19.6 (CH_3_). HRESIMS *m*/*z* 501.1662 [M + H]^+^ (calcd for C_27_H_30_O_7_Cl^+^, 501.1675).

Compound **33**: white, solid; yield, 55.7%; ^1^H NMR (400 MHz, CDCl_3_) *δ* 11.41 (1H, s), 7.62 (1H, dd, *J* = 6.0, 3.6 Hz), 7.35 (1H, dd, *J* = 6.0, 3.6 Hz), 7.24–7.20 (2H, overlapped), 6.56 (1H, dd, *J* = 16.1, 1.4 Hz), 6.42 (1H, s), 5.95 (1H, m), 5.45 (1H, ddd, *J* = 16.2, 8.7, 3.8 Hz), 5.32 (1H, m), 5.18 (1H, m), 4.70 (1H, t, *J* = 8.1 Hz), 4.32 (1H, m), 3.88 (3H, s), 3.64 (1H, dd, *J* = 8.1, 2.3 Hz), 2.90 (1H, m), 2.68 (1H, s), 2.51 (1H, m), 2.42–2.25 (2H, overlapped), 1.79 (3H, s), 1.37 (3H, d, *J* = 6.4 Hz). ^13^C NMR (100 MHz, CDCl_3_) *δ* 170.5 (C), 162.5 (C), 160.0 (C), 140.6 (C), 139.8 (C), 132.0 (C), 131.6 (CH), 131.4 (CH), 130.4 (CH), 130.2 (CH), 129.6 (CH), 129.0 (CH), 127.7 (CH), 126.7 (CH), 114.0 (C), 108.7 (CH), 106.2 (C), 99.5 (CH), 81.0 (CH), 76.4 (CH), 71.4 (CH), 68.3 (CH), 56.6 (OCH_3_), 37.1 (CH_2_), 34.9 (CH_2_), 26.5 (CH_3_), 19.1 (CH_3_). HRESIMS *m*/*z* 535.1282 [M + H]^+^ (calcd for C_27_H_29_O_7_Cl_2_^+^, 535.1285).

Compound **34**: white, solid; yield, 31.9%; ^1^H NMR (400 MHz, CDCl_3_) *δ* 11.36 (1H, s), 7.64 (1H, dd, *J* = 7.5, 2.0 Hz), 7.40 (1H, dd, *J* = 7.5, 1.7 Hz), 7.28 (1H, dd, *J* = 7.5, 1.7 Hz), 7.23 (1H, dd, J = 7.5, 2.0 Hz), 6.61 (1H, dd, *J* = 16.1, 1.5 Hz), 6.47 (1H, s), 6.06 (1H, m), 5.60–5.46 (2H, overlapped), 5.43 (1H, m), 4.51 (1H, t, *J* = 8.7 Hz), 4.24 (1H, m), 3.95 (1H, dd, *J* = 8.7, 2.4 Hz), 3.91 (3H, s), 2.76 (1H, m), 2.60 (1H, m), 2.47 (1H, m), 2.24 (1H, m), 2.01 (1H, s), 1.77 (3H, s), 1.39 (3H, d, *J* = 6.3 Hz). ^13^C NMR (100 MHz, CDCl_3_) *δ* 170.7 (C), 162.4 (C), 160.0 (C), 140.8 (C), 139.9 (C), 131.8 (CH), 131.7 (CH), 131.5 (CH), 130.7 (CH), 129.7 (CH × 2), 129.0 (CH), 127.4 (CH), 126.9 (CH), 114.1 (C), 108.9 (CH), 106.4 (C), 99.5 (CH), 82.4 (CH), 75.7 (CH), 71.3 (CH), 68.6 (CH), 56.6 (OCH_3_), 37.2 (CH_2_), 34.6 (CH_2_), 27.0 (CH_3_), 19.5 (CH_3_). HRESIMS *m*/*z* 535.1285 [M + H]^+^ (calcd for C_27_H_29_O_7_Cl_2_^+^, 535.1285).

Compound **35**: white, solid; yield, 49.3%; ^1^H NMR (400 MHz, CDCl_3_) *δ* 11.42 (1H, s), 6.60 (1H, dd, *J* = 16.1, 1.2 Hz), 6.47 (1H, s), 6.05 (1H, m), 5.52–5.34 (3H, overlapped), 4.61 (1H, t, *J* = 8.7 Hz), 4.17 (1H, m), 3.91 (3H, s), 3.63 (1H, dd, *J* = 8.7, 2.6 Hz), 2.86 (1H, m), 2.64–2.52 (2H, overlapped), 2.49–2.30 (2H, overlapped), 1.39 (3H, d, *J* = 6.4 Hz), 1.35 (3H, s), 0.94 (9H, s). ^13^C NMR (100 MHz, CDCl_3_) *δ* 170.7 (C), 162.5 (C), 159.9 (C), 140.1 (C), 130.9 (CH), 130.5 (CH), 129.9 (CH), 129.0 (CH), 114.1 (C), 114.1 (CH)106.4 (C), 99.5 (CH), 82.2 (CH), 75.4 (CH), 71.4 (CH), 68.1 (CH), 56.6 (OCH_3_), 39.3 (CH_2_), 37.3 (CH_2_), 34.4 (C), 25.4 (CH_3_ × 3), 20.9 (CH_3_), 19.4 (CH_3_). HRESIMS *m*/*z* 461.2519 [M + H]^+^ (calcd for C_26_H_37_O_7_^+^, 461.2534).

Compound **36**: white, solid; yield, 62.5%; ^1^H NMR (400 MHz, CDCl_3_) *δ* 11.42 (1H, s), 6.61 (1H, dd, *J* = 16.1, 1.4 Hz), 6.46 (1H, s), 6.02 (1H, m), 5.53–5.35 (3H, overlapped), 4.57 (1H, t, *J* = 8.7 Hz), 4.17 (1H, m), 3.91 (3H, s), 3.67 (1H, dd, *J* = 8.7, 2.5 Hz), 2.86 (1H, m), 2.64–2.51 (2H, overlapped), 2.48–2.30 (2H, overlapped), 1.84–1.61 (6H, overlapped), 1.39 (3H, d, *J* = 6.4 Hz), 1.32 (3H, s), 1.22–0.97 (5H, overlapped). ^13^C NMR (100 MHz, CDCl_3_) *δ* 170.7 (C), 162.5 (C), 160.0 (C), 140.0 (C), 130.7 (CH), 130.6 (CH), 130.4 (CH), 129.0 (CH), 114.1 (C), 112.0 (CH), 106.4 (C), 99.5 (CH), 81.6 (CH), 75.2 (CH), 71.5 (CH), 68.2 (CH), 56.6 (OCH_3_), 47.4 (CH_2_), 37.2 (CH_2_), 34.6 (C), 27.6 (CH), 27.4 (CH), 26.4 (CH), 26.4 (CH), 26.3 (CH), 22.7 (CH_3_), 19.4 (CH_3_). HRESIMS *m*/*z* 507.21 [M + H]^+^ (calcd for C_27_H_36_O_7_Cl^+^, 507.2144).

Compound **37**: white, solid; yield, 41.7%; ^1^H NMR (400 MHz, CDCl_3_) *δ* 11.38 (1H, s), 6.63 (1H, dd, *J* = 16.1, 1.4 Hz), 6.46 (1H, s), 5.97 (1H, m), 5.51 (1H, ddd, *J* = 16.1, 8.9, 3.7 Hz), 5.47–5.36 (2H, overlapped), 4.45 (1H, t, *J* = 8.7 Hz), 4.25 (1H, m), 3.91 (3H, s), 3.82 (1H, dd, *J* = 8.7, 2.4 Hz), 2.86 (1H, m), 2.60–2.52 (2H, overlapped), 2.48–2.28 (2H, overlapped), 1.89–1.62 (6H, overlapped), 1.38 (3H, d, *J* = 6.4 Hz), 1.26 (3H, s), 1.10 (5H, overlapped). ^13^C NMR (100 MHz, CDCl_3_) *δ* 170.7 (C), 162.4 (C), 160.0 (C), 140.0 (C), 131.5 (CH), 130.7 (CH), 130.4 (CH), 129.1 (CH), 114.1 (C), 111.7 (CH), 106.5 (C), 99.5 (CH), 80.4 (CH), 75.8 (CH), 71.4 (CH), 68.8 (CH), 56.6 (OCH_3_), 47.4 (CH_2_), 37.2 (CH_2_), 34.9 (CH), 27.7 (CH_2_×2), 26.4 (CH_2_×2), 26.3 (CH_2_), 22.7 (CH_3_ ), 19.5 (CH_3_ ). HRESIMS *m*/*z* 507.2162 [M + H]^+^ (calcd for C_27_H_36_O_7_Cl^+^, 507.2144).

### 3.5. Antimicrobial Activity

The broth dilution method was used to screen antibacterial activity in vitro. Broth micro- or macro-dilution is the most basic method for antibiotic susceptibility testing [38,40]. In the screening of anti-*M. marinum* activity, the commonly used isoniazid and rifampicin were selected as positive controls. Ciprofloxacin and chloramphenicol were positive in antibacterial tests. Amphotericin B was used as a positive drug in the antifungal test. The strains were inoculated into the corresponding medium, cultured at 32 °C for 8 h, and diluted to 10^5^ CFU/mL with the corresponding blank medium, and then a 198 μL bacterial solution and a 2 μL sample were added into the 96-well plates, using DMSO as a negative control. The treated 96-well plates were also cultured at 32 °C for 24 h or 48 h.

### 3.6. Statistical Analysis

GraphPad Prism 8 software was used for data analysis. Data are represented using the means ± SD. Data can only be considered statistically significant when *p* < 0.05. Data are presented as the means of three experiments.

## 4. Conclusions

In conclusion, 28 new derivatives with ketal groups were synthesized through a one-to-two-step chemical semi-synthetic reaction, enriching the structural diversity of the 14-membered RALs. In vitro activity evaluation of the synthesized derivatives showed that compounds **15**–**24** and **28**–**35** showed effective anti-*M. marinum* activity. Preliminary structure–activity relationship analysis of the compounds showed that the introduction of ketal groups with aromatic rings in C-5′ and C-6′ significantly enhanced the activity of the compounds. The 14-membered RALs showed no significant activity after the introduction of chain ketal groups at the C-5′ and C-6′ positions, but the activity increased significantly after the further introduction of chlorine atoms at C-5. The introduction of ketal groups to the hydroxyl groups at C-5′ and C-6′ and the ketone reaction, in which two carbonyl groups in the molecule are separated by two saturated carbon atoms, significantly increased anti-*M. marinum* activity. Further combined-administration experiments showed that compounds **23**, **24**, **29**, and **30** enhanced the anti-*M. marinum* activity of the positive drug. Therefore, compounds **23**, **24**, **29**, and **30** have effective anti-*M. marinum* activity in vitro, providing a new idea for the development of new anti-*M. marinum* drugs.

## Figures and Tables

**Table 1:** Key ^1^H NMR data (δ) for diastereoisomers *^a^*.

Compound	4′	5′	6′
**11**	4.19, m	3.68, dd (8.5, 2.5)	4.62, t (8.5)
**12**	4.32, m	3.93, dd (8.5, 2.3)	4.49, t (8.5)
**13**	4.20, m	3.74, dd (8.4, 2.4)	4.58, t (8.4)
**14**	4.27, m	3.91, dd 8.4, 2.3)	4.49, t (8.4)
**15**	4.32, m	3.73, dd (7.3, 2.2)	4.70, dd (9.5, 7.3)
**16**	4.22, m	4.03, dd (8.3, 2.2)	4.59, t (8.3)
**17**	4.31, m	3.71, dd (7.3, 2.2)	4.69, dd (9.5, 7.3)
**18**	4.22, m	4.02, dd (8.4, 2.2)	4.58, t (8.4)
**19**	4.34, m	3.73, dd (7.3, 2.2)	4.72, dd (9.4, 7.3)
**20**	4.26, m	4.02, dd (8.4, 2.3)	4.55, t (8.4)
**21**	4.30, m	3.69, dd (7.3, 2.2)	4.69, dd (9.4, 7.3)
**22**	4.22, m	4.02, dd (8.3, 2.2)	4.56, t (8.3)
**23**	4.19, m	3.79, dd (8.5, 2.4)	4.60, t (8.5)
**24**	4.21, m	3.90, dd (8.3, 2.1)	4.58, t (8.3)
**25**	4.23, m	3.89, dd (8.4, 2.3)	4.62, t (8.4)
**26**	4.26, m	3.96, dd (7.6, 2.1)	4.62, t (7.6)
**29**	4.17, m	3.71, dd (8.7, 2.5)	4.59, t (8.7)
**30**	4.20, m	3.81, dd (8.6, 2.2)	4.56, t (8.6)
**31**	4.30, m	3.65, dd (9.0, 2.4)	4.69, t (9.0)
**32**	4.20, m	3.94, dd (8.6, 2.2)	4.53, t (8.6)
**33**	4.32, m	3.64, dd (8.1, 2.3)	4.70, t (8.1)
**34**	4.24, m	3.95, dd (8.7, 2.4)	4.51, t (8.7)
**36**	4.17, m	3.67, dd (8.7, 2.5)	4.57, t (8.7)
**37**	4.25, m	3.82, dd (8.7, 2.4)	4.45, t (8.7)

*^a^* Solvent: CDCl_3_.

**Table 2:** Antimicrobial activity of representative compounds of the RALs with ketal groups ^1^.

Compound	MIC_90_ (µM)					
	*M. marinum*	*S. aureus*	*E. coli*	*P. aeruginosa*	*C. albicans*	*V. vulnificus*
**11**	>200	>100	>100	>100	>100	>100
**15**	80	25	>100	>100	>100	>100
**16**	80	>100	>100	>100	>100	>100
**17**	80	>100	>100	>100	>100	>100
**18**	70	>100	>100	>100	>100	>100
**19**	70	>100	>100	>100	>100	>100
**20**	70	>100	>100	>100	>100	>100
**21**	80	25	>100	>100	>100	>100
**22**	80	>100	nt	>100	>100	nt
**23**	80	>100	>100	>100	>100	>100
**24**	70	>100	>100	>100	>100	25
**28**	70	>100	>100	>100	>100	>100
**29**	70	50	>100	>100	>100	>100
**30**	80	>100	25	>100	>100	>100
**31**	80	>100	>100	>100	>100	>100
**32**	80	>100	nt	>100	>100	nt
**33**	80	>100	nt	>100	>100	nt
**34**	90	>100	nt	>100	>100	nt
**35**	80	>100	nt	>100	>100	nt
Isoniazid	40	nt	nt	nt	nt	nt
Rifampicin	10	nt	nt	nt	nt	nt
Ciprofloxacin	nt	3.13	0.10	1.56	nt	nt
Amphotericin B	nt	nt	nt	nt	0.84	nt
Chloramphenicol	nt	nt	nt	nt	nt	9.75

^1^ Results are the average of three independent experiments, each performed in duplicate. Standard deviations were less than ±10%. nt = not tested.

**Table 3:** Anti-*M. marinum* effects of compounds **23**, **24**, **29**, and **30** in combination with the positive drug isoniazid.

	Isoniazid MIC_90_ (µM)		Compounds MIC_90_ (µM)		FICI ^1^	Mode of Action
	Alone	Combined	Alone	Combined		
**23**	40	20	80	40	1	additive
**24**	40	20	70	8.75	0.625	additive
**29**	40	20	70	8.75	0.625	additive
**30**	40	20	80	40	1	additive

^1^ The mode of action was determined via fractional inhibitory concentration index (FICI): (1) FICI ≤ 0.5, synergistic effect; (2) 0.5 < FICI ≤ 1, additive effect; (3) 1 < FICI ≤ 2, irrelevant; (4) FICI > 2, antagonistic effect.

**Table 4:** Anti-*M. marinum* effects of compounds **23**, **24**, **29**, and **30** in combination with the positive drug rifampicin.

	Rifampicin MIC_90_ (µM)		Compounds MIC_90_ (µM)		FICI ^1^	Mode of Action
	Alone	Combined	Alone	Combined		
**23**	10	5	80	20	0.75	additive
**24**	10	5	70	8.75	0.625	additive
**29**	10	5	70	8.75	0.625	additive
**30**	10	5	80	20	0.75	additive

^1^ The mode of action was determined via fractional inhibitory concentration index (FICI): (1) FICI ≤ 0.5, synergistic effect; (2) 0.5 < FICI ≤ 1, additive effect; (3) 1 < FICI ≤ 2, irrelevant; (4) FICI > 2, antagonistic effect.

## Data Availability

The data are contained within the article or Supplementary Material.

## Supplementary Materials

The following supporting information can be downloaded at https://www.mdpi.com/article/10.3390/md22100431/s1. Table S1: Different ketone reagents used to generate compounds **10**–**37**. Table S2: Key ^13^C NMR data (δ) for diastereoisomers. Figure S1–S98: ^1^H NMR, ^13^C NMR, 1D NOE, and HRESIMS results for compounds **10**–**37**.
