# Supplementary material for: Semisynthesis, Structure Elucidation and Anti-Mycobacterium marinum Activity of a Series of Marine-Derived 14-Membered Resorcylic Acid Lactones with Interesting Ketal Groups
Source: Mar Drugs. 2024 Sep 25;22(10):431. doi: 10.3390/md22100431 (PMC11509596; doi:10.3390/md22100431)
Supplement: Supplementary file 1 [file marinedrugs-22-00431-s001.zip › marinedrugs-3223680-supplementary.pdf]

DerivativesSemisynthesis, Structure Elucidation and Anti-*Mycobacterium marinum* Activity of a Series of Marine-Derived 14-Membered Resorcylic Acid Lactones with Interesting Ketal groups

Jun-Na Yin [1,†], Cui-Fang Wang [1,†], Xiu-Li Zhang [1], Ya-Jie Cheng [1], Yan-Wei Wu [1], Qun Zhang [1], Chang-Lun Shao [1,2], Mei-Yan Wei [1,2,*] and Yu-Cheng Gu [3,*]

[1] Key Laboratory of Marine Drugs, The Ministry of Education of China, School of Medicine and Pharmacy, Ocean University of China, Qingdao 266003, China; yinjunna@163.com (J.-N.Y.); wangcuifang0115@163.com (C.-F.W.); xiulizhang@ouc.edu.cn (X.-L.Z.); yajiecheng1212@163.com (Y.-J.C.); wuyanwei1214@163.com (Y.-W.W.); zhangqunnn@163.com (Q.Z.); shaochanglun@163.com (C.-L.S.).

[2] Key Laboratory of Tropical Medicinal Resource Chemistry of Ministry of Education, College of Chemistry and Chemical Engineering, Hainan Normal University, Haikou 571158, China

[3] Syngenta Jealott's Hill International Research Centre, Bracknell, Berkshire RG42 6EY, UK

* Correspondence: mywei95@126.com (M.-Y.W.); yucheng.gu@syngenta.com (Y.-C.G.)

† These authors contributed equally to this work.

Content of Supporting Information

Table S1. Different ketone reagents used to generate compounds **10**–**37**.

Table S2. Key ^{13}C NMR data (δ) for diastereoisomers.[a]

Figure S1. ^1H NMR (400 MHz, Chloroform-*d*) spectrum of compound **10**.

Figure S2. ^{13}C NMR (100 MHz, Chloroform-*d*) spectrum of compound **10**.

Figure S3. HR-ESI-MS spectrum of compound **10.**

Figure S4. ^1H NMR (400 MHz, Chloroform-*d*) spectrum of compound **11**.

Figure S5. ^{13}C NMR (100 MHz, Chloroform-*d*) spectrum of compound **11**.

Figure S6. 1D NOE spectrum of compound **11**

Figure S7. HR-ESI-MS spectrum of compound **11**.

Figure S8. ^1H NMR (400 MHz, Chloroform-*d*) spectrum of compound **12**.

Figure S9. ^{13}C NMR (100 MHz, Chloroform-*d*) spectrum of compound **12**.

Figure S10. 1D NOE spectrum of compound **12.**

Figure S11. HR-ESI-MS spectrum of compound **12**.

Figure S12. ^1H NMR (400 MHz, Chloroform-*d*) spectrum of compound **13**.

Figure S13. ^{13}C NMR (100 MHz, Chloroform-*d*) spectrum of compound **13**.

Figure S14. 1D NOE spectrum of compound **13**

Figure S15. HR-ESI-MS spectrum of compound **13**.

Figure S16. ^1H NMR (400 MHz, Chloroform-*d*) spectrum of compound **14**.

Figure S17. ^{13}C NMR (100 MHz, Chloroform-*d*) spectrum of compound **14**.

Figure S18. 1D NOE spectrum of compound **14**

Figure S19. HR-ESI-MS spectrum of compound **14**.

Figure S20. ^1H NMR (400 MHz, Chloroform-*d*) spectrum of compound **15**.

Figure S21. ^{13}C NMR (125 MHz, Chloroform-*d*) spectrum of compound **15**.

Figure S22. 1D NOE spectrum of compound **15**

Figure S23. HR-ESI-MS spectrum of compound **15**.

Figure S24. ^1H NMR (400 MHz, Chloroform-*d*) spectrum of compound **16**.

Figure S25. ^{13}C NMR (125 MHz, Chloroform-*d*) spectrum of compound **16**.

Figure S26. 1D NOE spectrum of compound **16**

Figure S27. HR-ESI-MS spectrum of compound **16**.

Figure S28. ^1H NMR (400 MHz, Chloroform-*d*) spectrum of compound **17**.

Figure S29. ^{13}C NMR (100 MHz, Chloroform-*d*) spectrum of compound **17**.

Figure S30. HR-ESI-MS spectrum of compound **17**.

Figure S31. ^1H NMR (400 MHz, Chloroform-*d*) spectrum of compound **18**.

Figure S32. ^{13}C NMR (100 MHz, Chloroform-*d*) spectrum of compound **18.**

Figure S33. HR-ESI-MS spectrum of compound **18**.

Figure S34. ^1H NMR (400 MHz, Chloroform-*d*) spectrum of compound **19.**

Figure S35. ^{13}C NMR (100 MHz, Chloroform-*d*) spectrum of compound **19**.

Figure S36. HR-ESI-MS spectrum of compound **19**.

Figure S37. ^1H NMR (400 MHz, Chloroform-*d*) spectrum of compound **20**.

Figure S38. ^{13}C NMR (100 MHz, Chloroform-*d*) spectrum of compound **20**.

Figure S39. HR-ESI-MS spectrum of compound **20**.

Figure S40. ^1H NMR (400 MHz, Chloroform-*d*) spectrum of compound **21.**

Figure S41. ^{13}C NMR (100 MHz, Chloroform-*d*) spectrum of compound **21**.

Figure S42. HR-ESI-MS spectrum of compound **21**.

Figure S43. ^1H NMR (400 MHz, Chloroform-*d*) spectrum of compound **22.**

Figure S44. ^{13}C NMR (100 MHz, Chloroform-*d*) spectrum of compound **22**.

Figure S45. HR-ESI-MS spectrum of compound **22**.

Fgure S46. ^1H NMR (400 MHz, Chloroform-*d*) spectrum of compound **23**.

Figure S47. ^{13}C NMR (100 MHz, Chloroform-*d*) spectrum of compound **23**.

Figure S48. 1D NOE spectrum of compound **23**

Figure S49. HR-ESI-MS spectrum of compound **23**.

Figure S50. ^1H NMR (400 MHz, Chloroform-*d*) spectrum of compound **24**.

Figure S51. ^{13}C NMR (100 MHz, Chloroform-*d*) spectrum of compound **24.**
Figure S52. 1D NOE spectrum of compound **24.**
Figure S53. HR-ESI-MS spectrum of compound **24**.
Figure S54. ^{1}H NMR (400 MHz, Chloroform-*d*) spectrum of compound **25.**
Figure S55. ^{13}C NMR (100 MHz, Chloroform-*d*) spectrum of compound **25**.
Figure S56. 1D NOE spectrum of compound **25**
Figure S57. HR-ESI-MS spectrum of compound **25**.
Figure S58. ^{1}H NMR (400 MHz, Chloroform-*d*) spectrum of compound **26.**
Figure S59. ^{13}C NMR (100 MHz, Chloroform-*d*) spectrum of compound **26.**
Figure S60. 1D NOE spectrum of compound **26**
Figure S61. HR-ESI-MS spectrum of compound **26**.
Figure S62. ^{1}H NMR (400 MHz, Chloroform-*d*) spectrum of compound **27.**
Figure S63. ^{13}C NMR (100 MHz, Chloroform-*d*) spectrum of compound **27.**
Figure S64. HR-ESI-MS spectrum of compound **27**.
Figure S65. ^{1}H NMR (400 MHz, Chloroform-*d*) spectrum of compound **28.**
Figure S66. ^{13}C NMR (100 MHz, Chloroform-*d*) spectrum of compound 28
Figure S67. 1D NOE spectrum of compound **28**
Figure S68. HR-ESI-MS spectrum of compound **28**.
Figure S69. ^{1}H NMR (400 MHz, Chloroform-*d*) spectrum of compound **29**.
Figure S70. ^{13}C NMR (125 MHz, Chloroform-*d*) spectrum of compound **29**.
Figure S71. 1D NOE spectrum of compound **29.**
Figure S72. HR-ESI-MS spectrum of compound **29**.
Figure S73. ^{1}H NMR (400 MHz, Chloroform-*d*) spectrum of compound **30.**
Figure S74. ^{13}C NMR (100 MHz, Chloroform-*d*) spectrum of compound **30.**
Figure S75. 1D NOE spectrum of compound **30.**
Figure S76. HR-ESI-MS spectrum of compound **30**.
Figure S77. ^{1}H NMR (400 MHz, Chloroform-*d*) spectrum of compound **31.**
Figure S78. ^{13}C NMR (100 MHz, Chloroform-*d*) spectrum of compound **31**.
Figure S79. 1D NOE spectrum of compound **31.**
Figure S80. HR-ESI-MS spectrum of compound **31**.
Figure S81. ^{1}H NMR (400 MHz, Chloroform-*d*) spectrum of compound **32.**
Figure S82. ^{13}C NMR (100 MHz, Chloroform-*d*) spectrum of compound **32.**
Figure S83. HR-ESI-MS spectrum of compound **32**.
Figure S84. ^{1}H NMR (400 MHz, Chloroform-*d*) spectrum of compound **33.**
Figure S85. ^{13}C NMR (100 MHz, Chloroform-*d*) spectrum of compound **33**.
Figure S86. HR-ESI-MS spectrum of compound **33**.
Figure S87. ^{1}H NMR (400 MHz, Chloroform-*d*) spectrum of compound **34.**
Figure S88. ^{13}C NMR (100 MHz, Chloroform-*d*) spectrum of compound **34**.
Figure S89. HR-ESI-MS spectrum of compound **34**.
Figure S90. ^{1}H NMR (400 MHz, Chloroform-*d*) spectrum of compound **35.**
Figure S91. ^{13}C NMR (100 MHz, Chloroform-*d*) spectrum of compound **35.**
Figure S92. HR-ESI-MS spectrum of compound **35**.
Figure S93. ^{1}H NMR (400 MHz, Chloroform-*d*) spectrum of compound **36**.
Figure S94. ^{13}C NMR (100 MHz, Chloroform-*d*) spectrum of compound **36**.
Figure S95. HR-ESI-MS spectrum of compound **36**.
Figure S96. ^{1}H NMR (400 MHz, Chloroform-*d*) spectrum of compound **37.**
Figure S97. ^{13}C NMR (100 MHz, Chloroform-*d*) spectrum of compound **37**.
Figure S98. HR-ESI-MS spectrum of compound **37**.

Table S1. Different ketone reagents used to generate compounds **10–37**.

No.	reagents	yield	No.	reagents	yield
**10**	3-Pentanone	93%	**24**	2,5-Hexanedione	35.2%
**11**	Pinacolone	79.4%	**25**	Acetylacetone	54.4%
**12**	Pinacolone	24.1%	**26**	Acetylacetone	36.3%
**13**	Cyclohexyl methyl ketone	69.7%	**27**	3-Pentanone	58.8%
**14**	Cyclohexyl methyl ketone	33.2%	**28**	4'-Chloroacetophenone	54.5%
**15**	Acetophenone	63.7%	**29**	2,5-Hexanedione	53.9%
**16**	Acetophenone	43.1%	**30**	2,5-Hexanedione	32.3%
**17**	4-Fluoroacetophenone	64.8%	**31**	Acetophenone	58.5%
**18**	4-Fluoroacetophenone	41.7%	**32**	Acetophenone	37.6%
**19**	2'-Chloroacetophenone	65.4%	**33**	2'-Chloroacetophenone	55.7%
**20**	2'-Chloroacetophenone	33.8%	**34**	2'-Chloroacetophenone	31.9%
**21**	4'-Chloroacetophenone	56.6%	**35**	Pinacolone	49.3%
**22**	4'-Chloroacetophenone	32.3%	**36**	Cyclohexyl methyl ketone	62.5%
**23**	2,5-Hexanedione	52.8%	**37**	Cyclohexyl methyl ketone	41.7%

[a]All reagents used in this study are commercial reagents.

Table S2. Key ^{13}C NMR data (δ) for diastereoisomers.[a]

compound	4'	5'	6'
**11**	68.7, CH	82.9, CH	75.1, CH
**12**	69.7, CH	80.8, CH	75.9, CH
**13**	68.8, CH	82.1, CH	75.0, CH)
**14**	69.4, CH	80.9, CH	75.5, CH
**15**	69.1, CH	81.6, CH	76.5, CH
**16**	69.4, CH	82.9, CH	75.7, CH
**17**	69.0, CH	81.7, CH	76.5, CH
**18**	69.2, CH	82.9, CH	75.8, CH
**19**	69.0, CH	81.6, CH	76.3, CH
**20**	69.2, CH	82.9, CH	75.5, CH
**21**	69.0, CH	81.7, CH	76.5, CH
**22**	69.2, CH	82.9, CH	75.9, CH
**23**	68.8, CH	82.3, CH	75.4, CH
**24**	69.0, CH	81.9, CH	77.4, CH
**25**	68.6, CH	82.1, CH	75.5, CH
**26**	69.1, CH	82.1, CH	76.0, CH
**29**	68.2, CH	81.8, CH	75.6, CH
**30**	68.5, CH	81.4, CH	75.9, CH
**31**	68.4, CH	81.0, CH	76.6, CH
**32**	68.8, CH	82.4, CH	75.9, CH
**33**	68.3, CH	81.0, CH	76.4, CH
**34**	68.6, CH	82.4, CH	75.7, CH
**36**	68.2, CH	81.6, CH	75.2, CH
**37**	68.8, CH	80.4, CH	75.8, CH

[a] Solvent: $CDCl_3$.

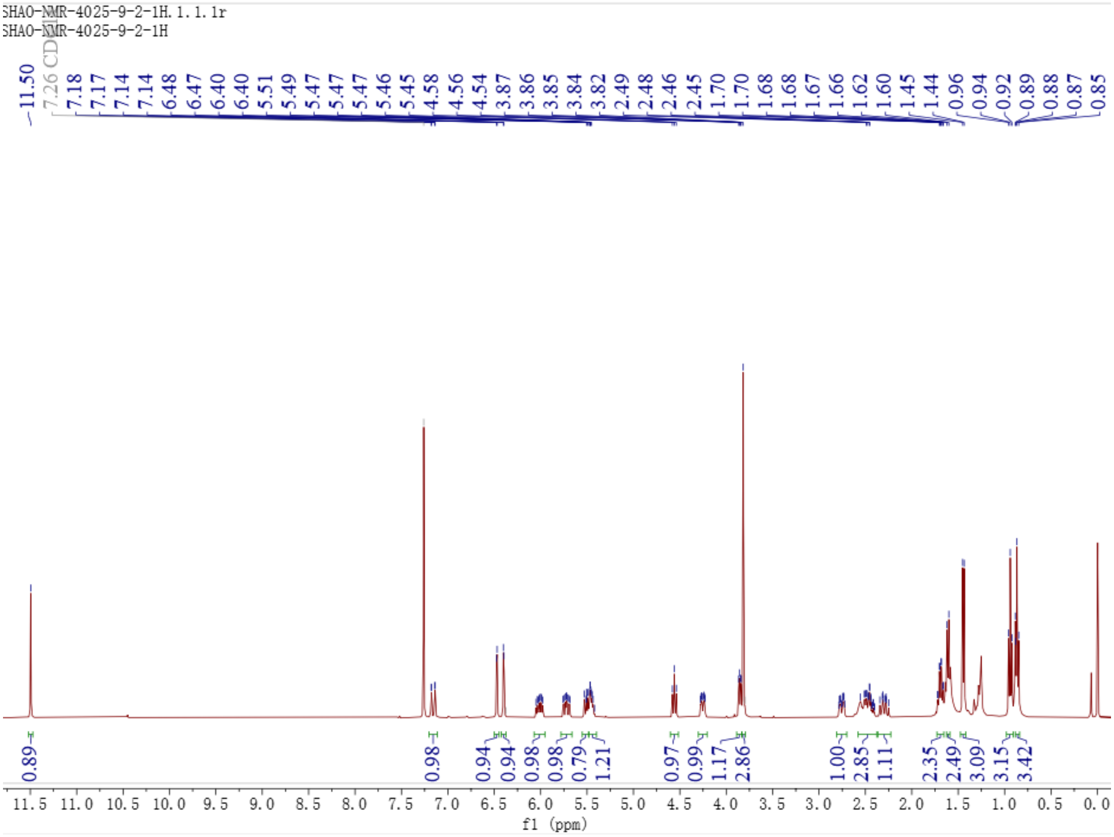

Figure S1. ^{1}H NMR (400 MHz, Chloroform-*d*) spectrum of compound **10**.

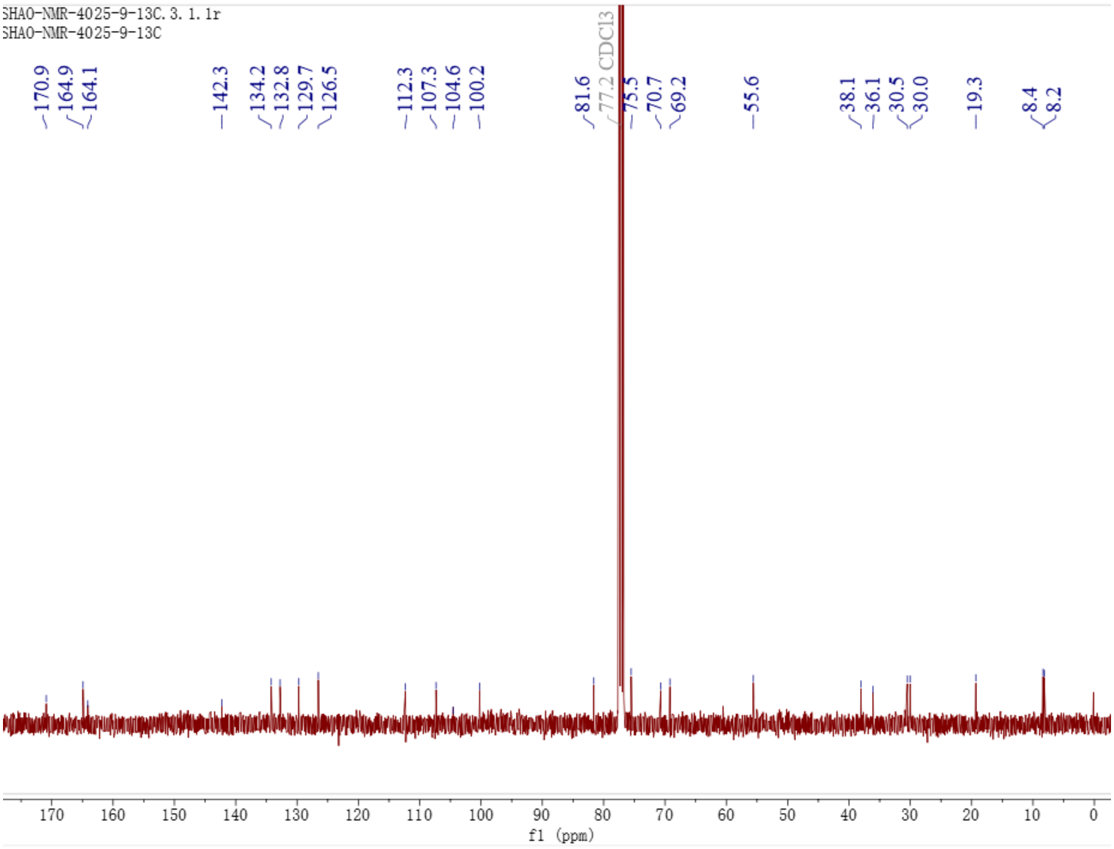

Figure S2. ^{13}C NMR (100 MHz, Chloroform-*d*) spectrum of compound **10**.

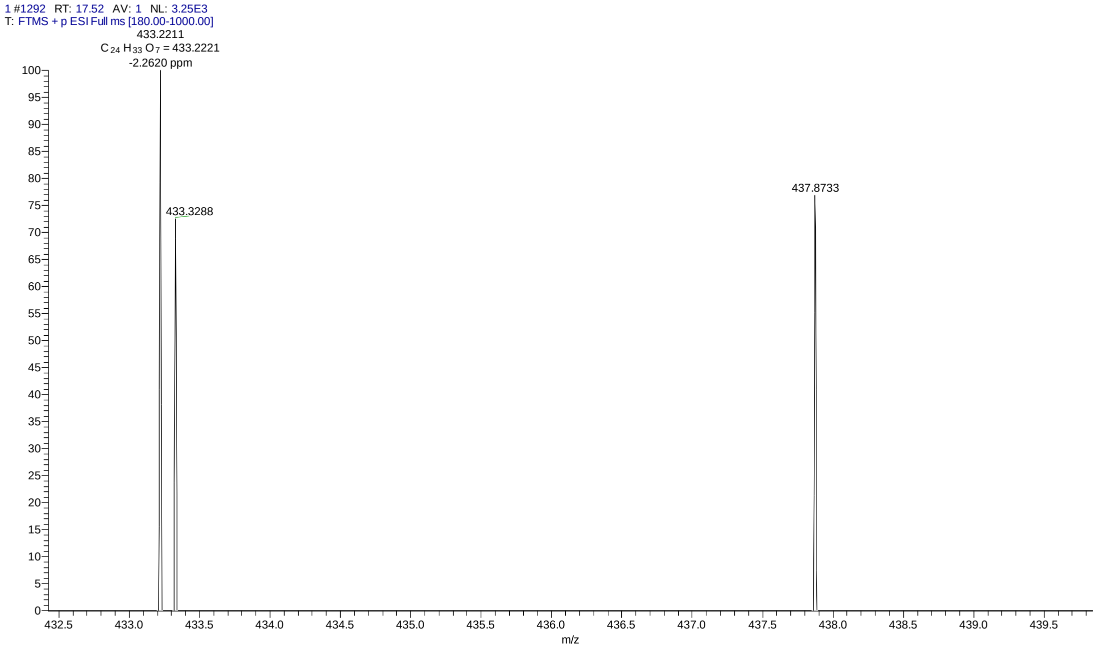

Figure S3. HR-ESI-MS spectrum of compound **10**.

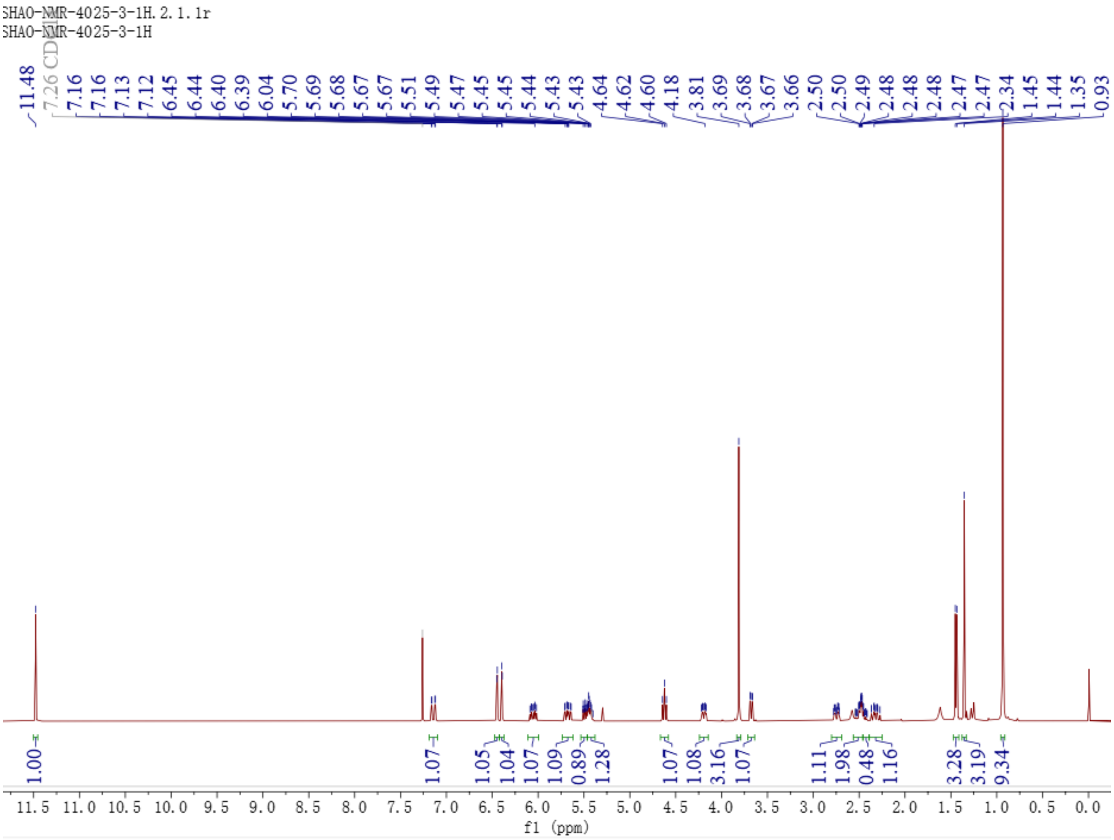

Figure S4. ^{1}H NMR (400 MHz, Chloroform-*d*) spectrum of compound **11**.

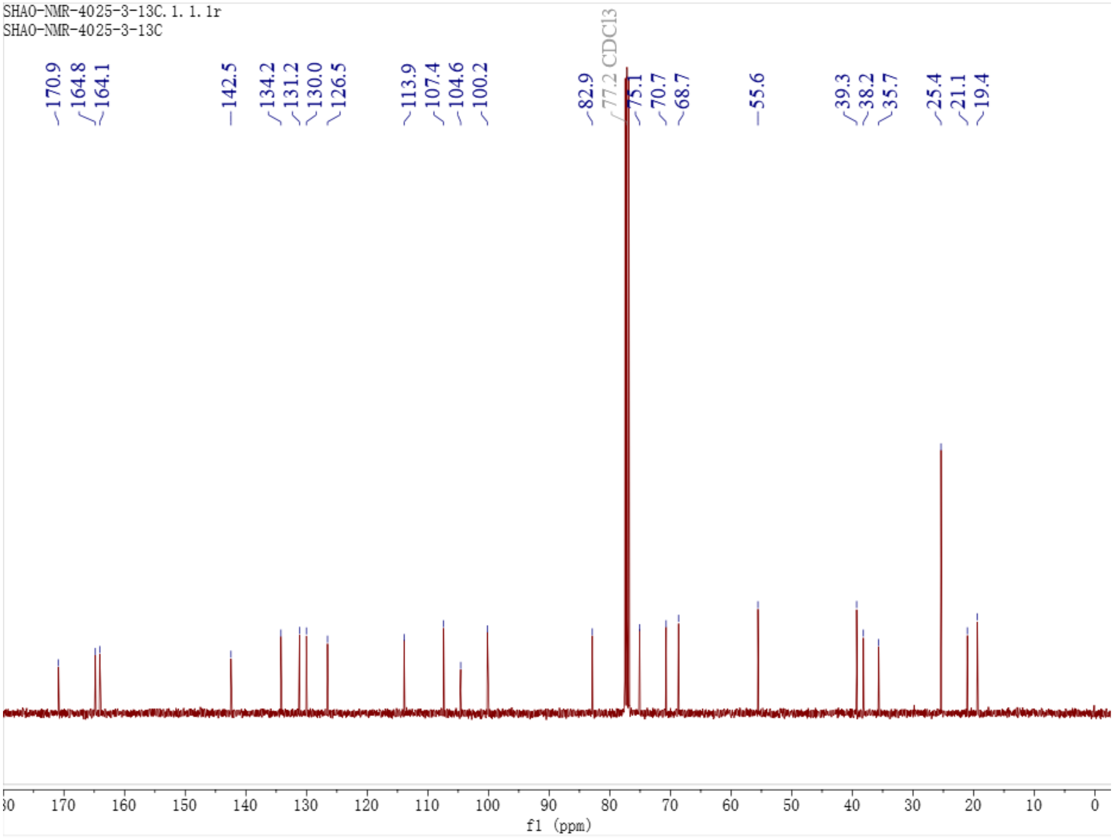

Figure S5. ^{13}C NMR (100 MHz, Chloroform-*d*) spectrum of compound **11**.

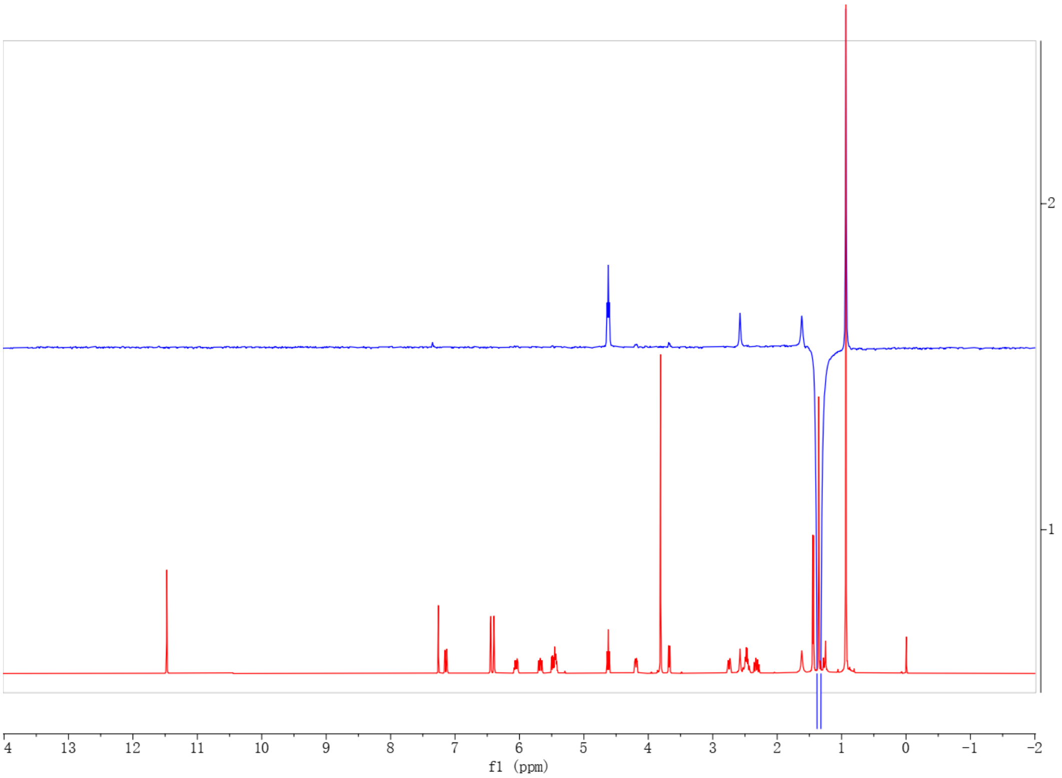

Figure S6. 1D NOE spectrum of compound **11**

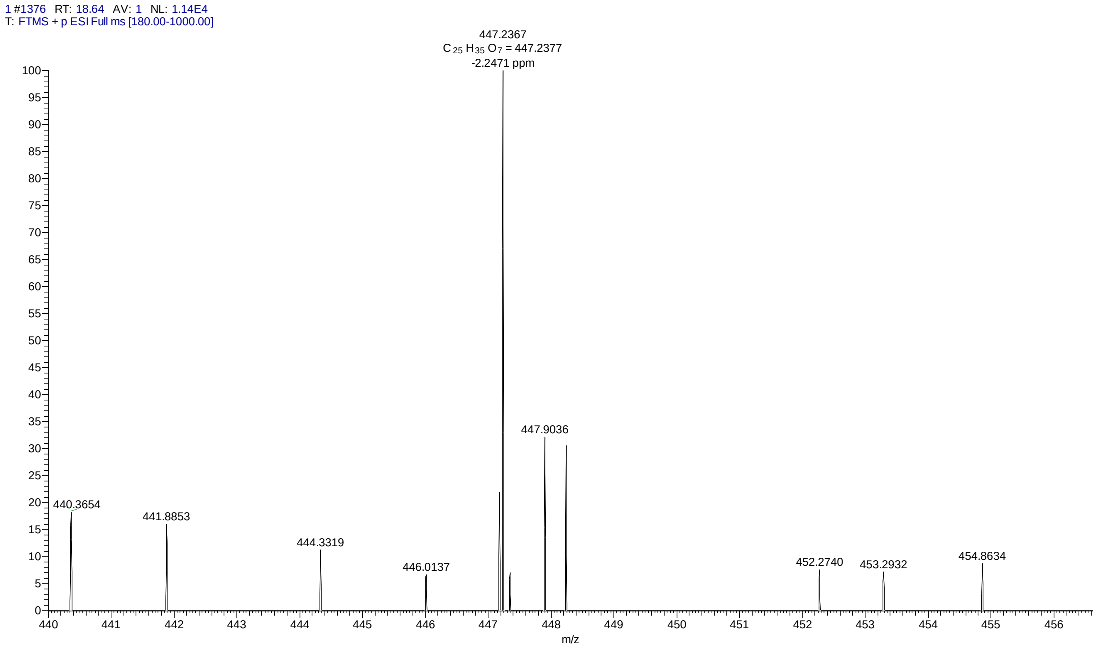

Figure S7. HR-ESI-MS spectrum of compound **11**.

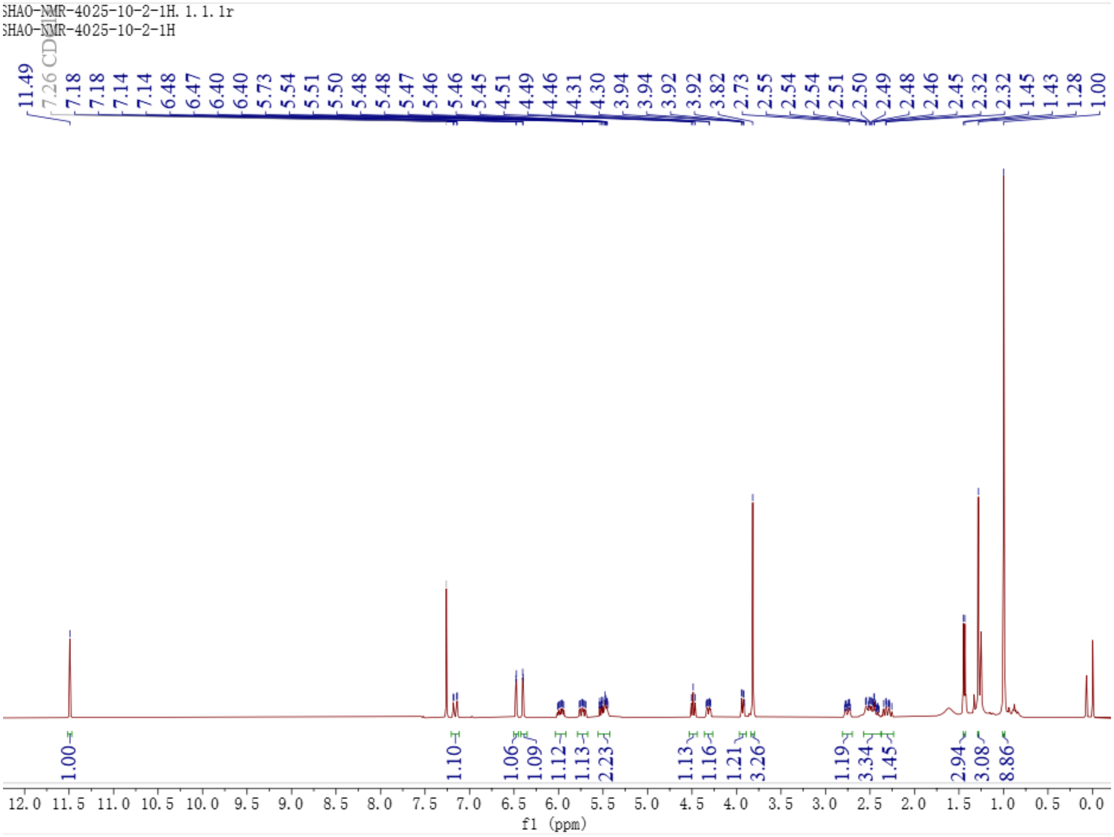

Figure S8. ^{1}H NMR (400 MHz, Chloroform-*d*) spectrum of compound **12**.

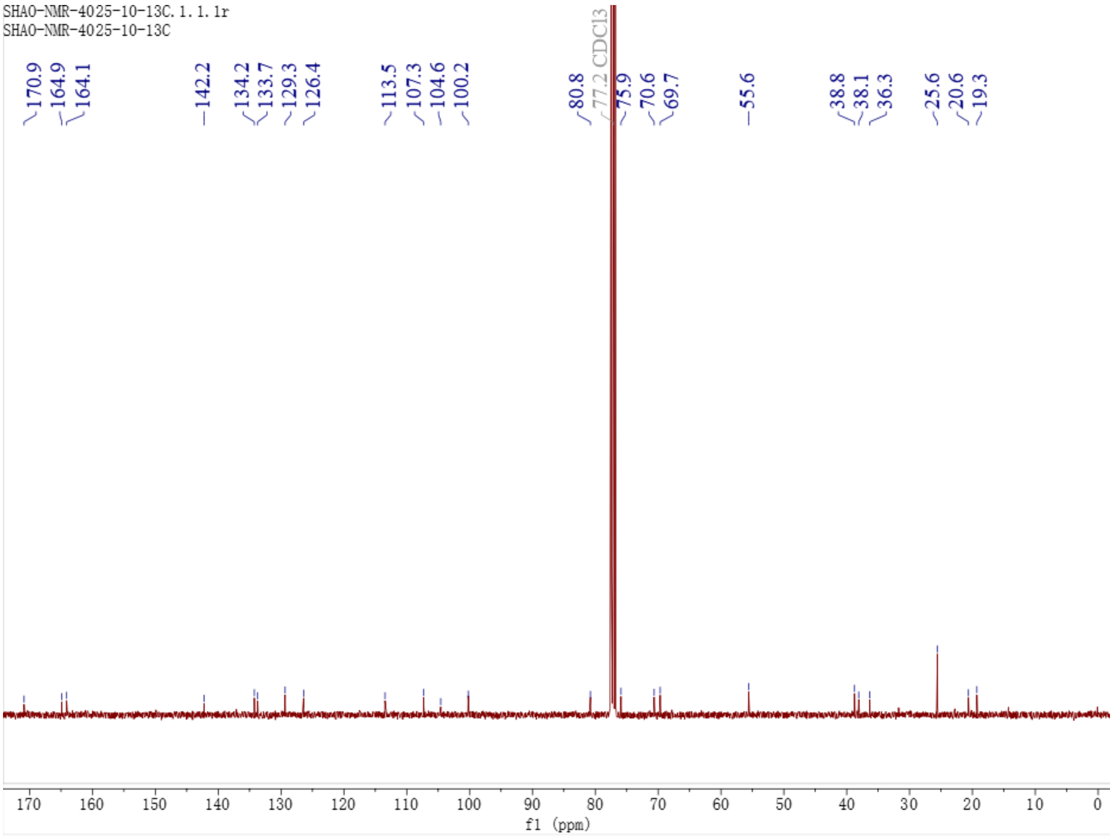

Figure S9. ${}^{13}C$ NMR (100 MHz, Chloroform-*d*) spectrum of compound **12**.

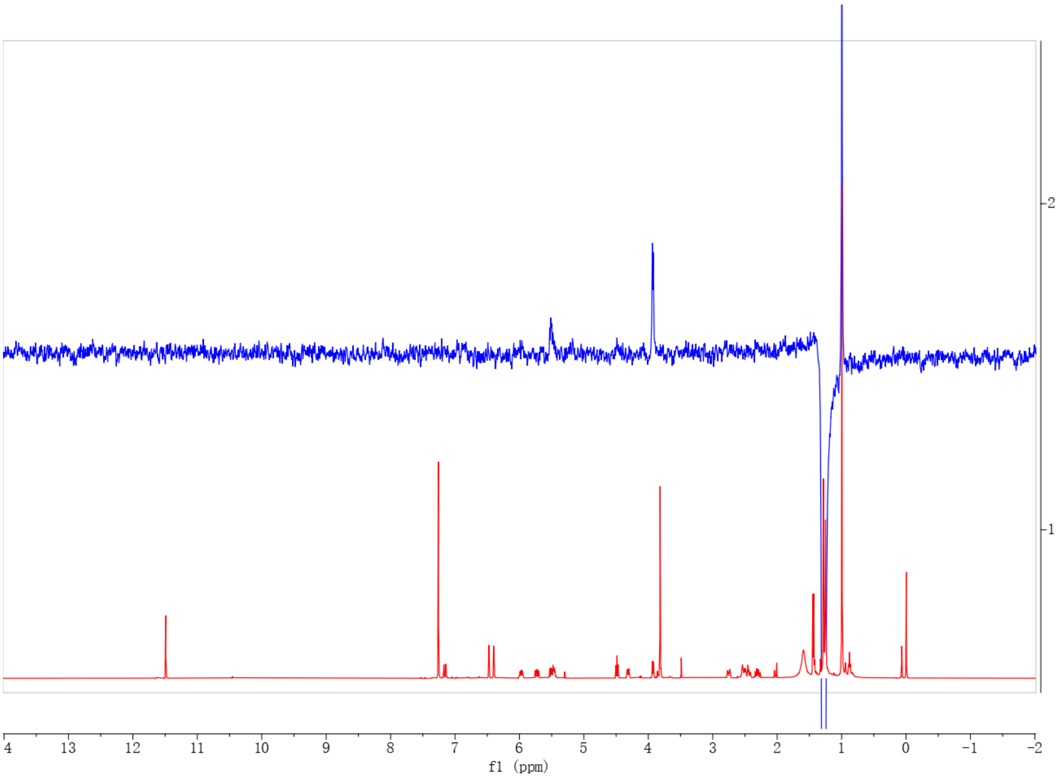

Figure S10. 1D NOE spectrum of compound **12.**

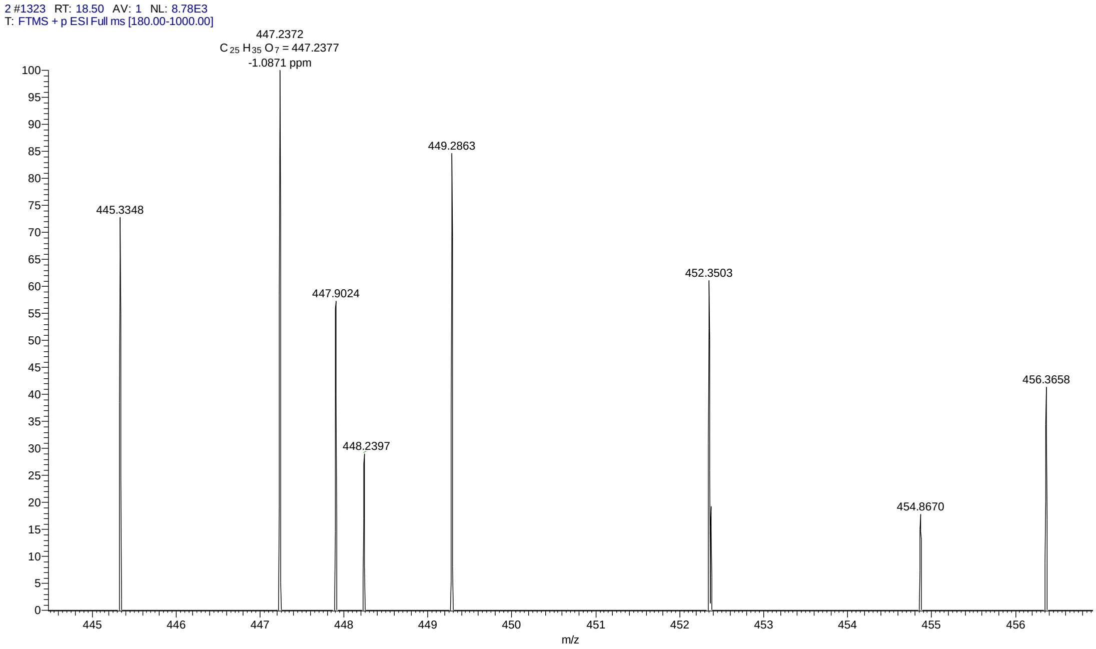

Figure S11. HR-ESI-MS spectrum of compound **12**.

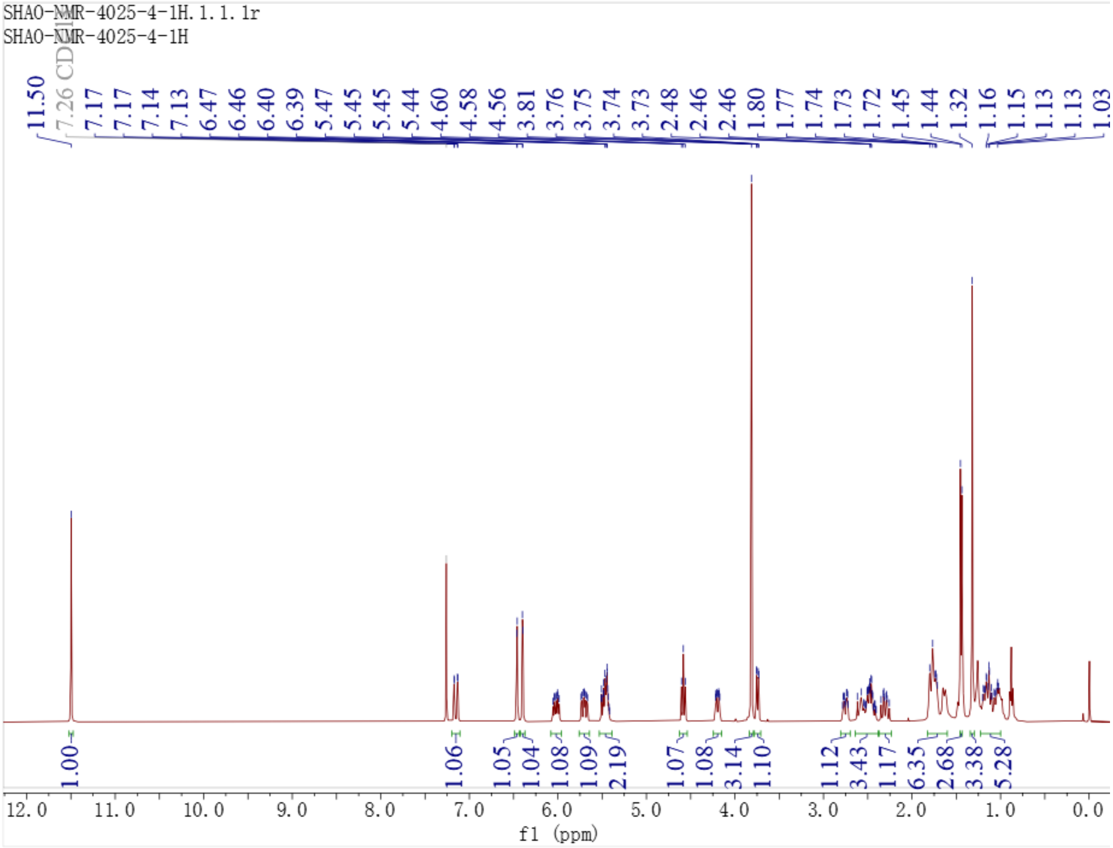

Figure S12. ^{1}H NMR (400 MHz, Chloroform-*d*) spectrum of compound **13**.

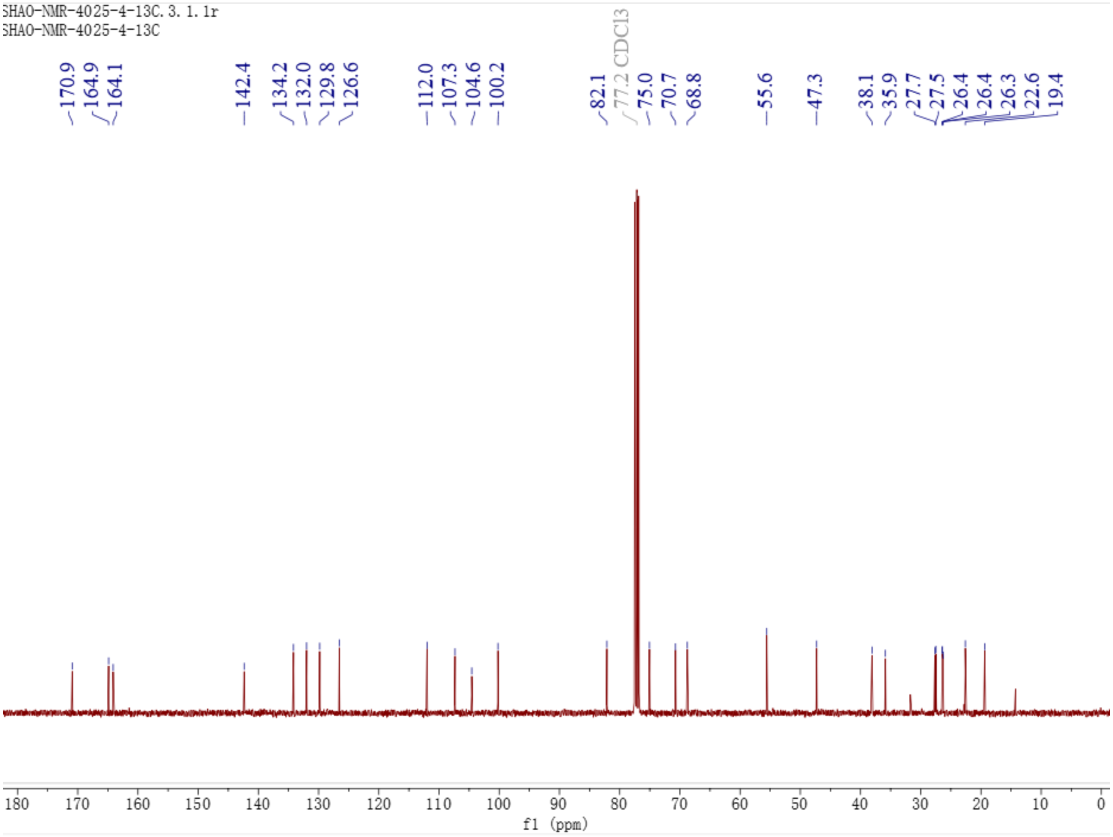

Figure S13. ^{13}C NMR (100 MHz, Chloroform-*d*) spectrum of compound **13**.

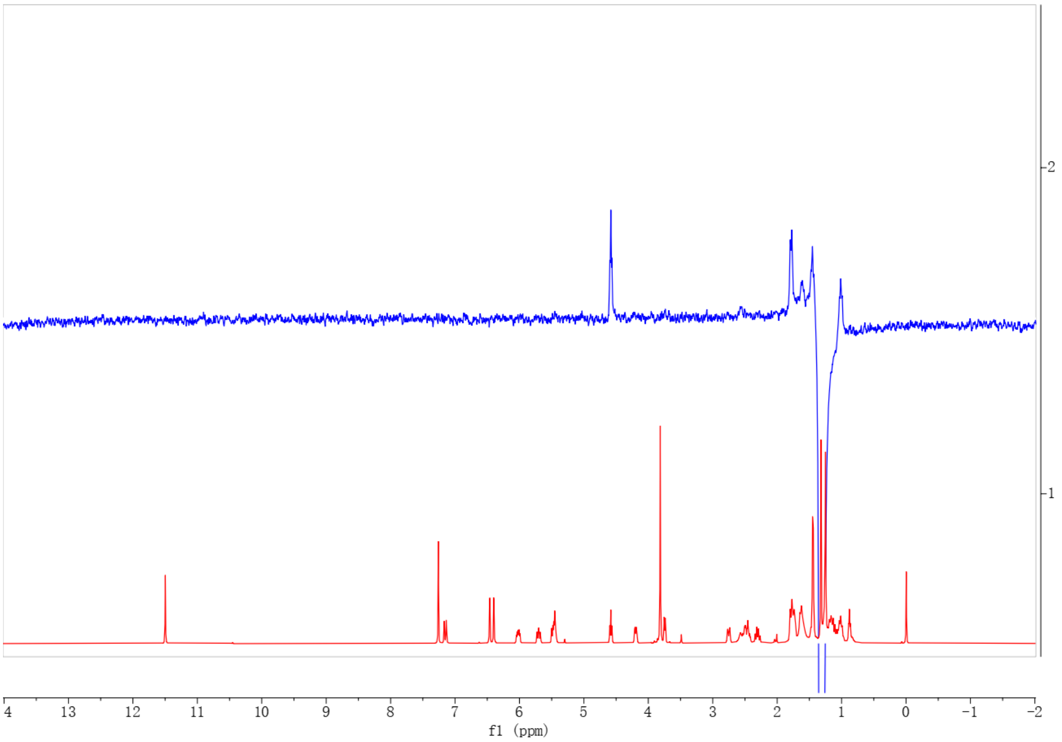

Figure S14. 1D NOE spectrum of compound **13**

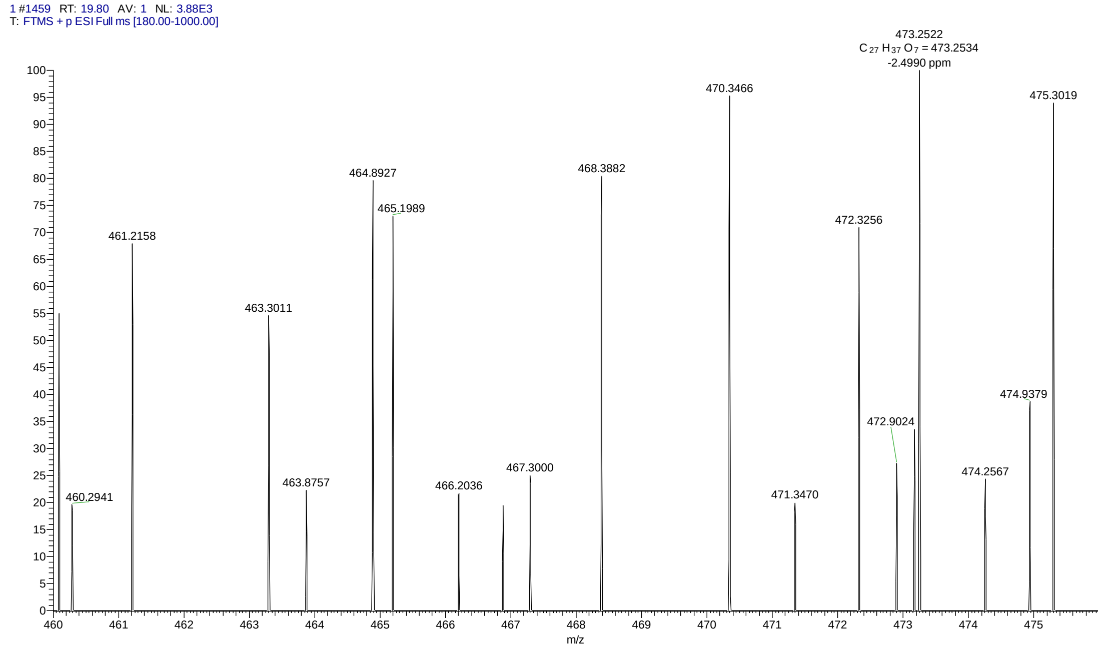

Figure S15. HR-ESI-MS spectrum of compound **13**.

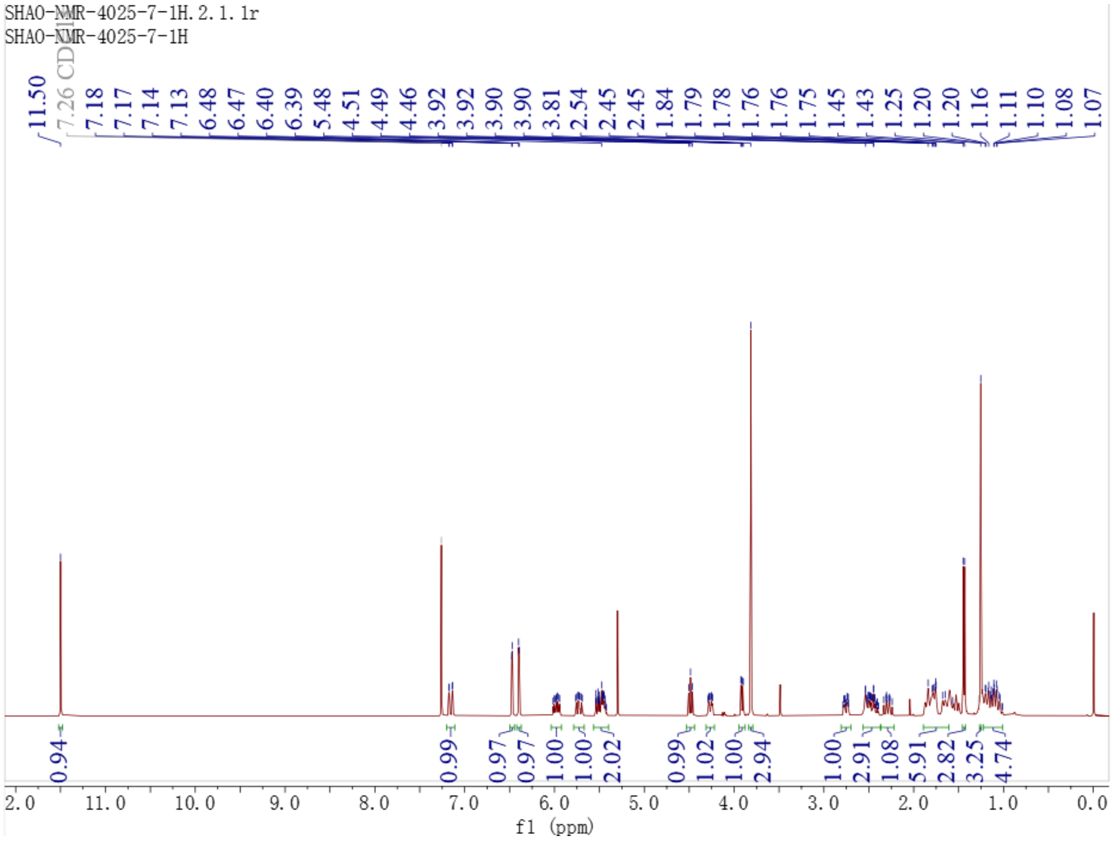

Figure S16. ^{1}H NMR (400 MHz, Chloroform-*d*) spectrum of compound **14**.

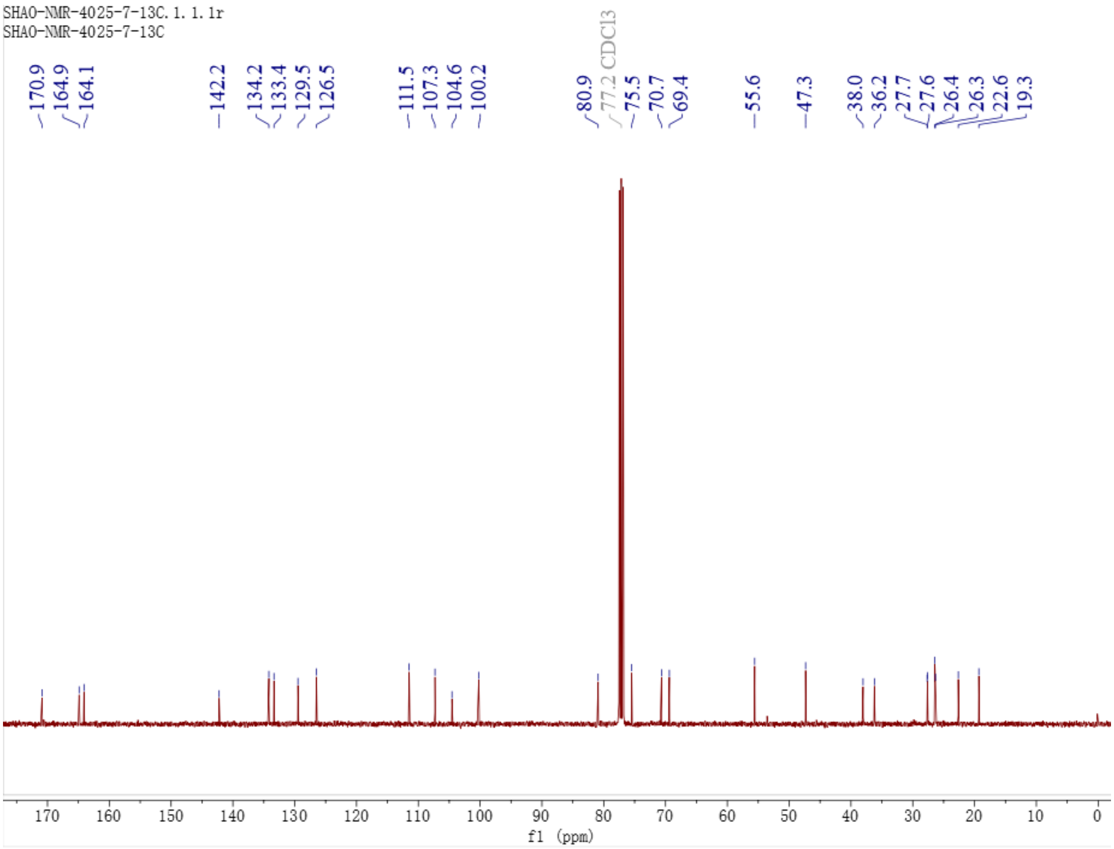

Figure S17. ^{13}C NMR (100 MHz, Chloroform-*d*) spectrum of compound **14**.

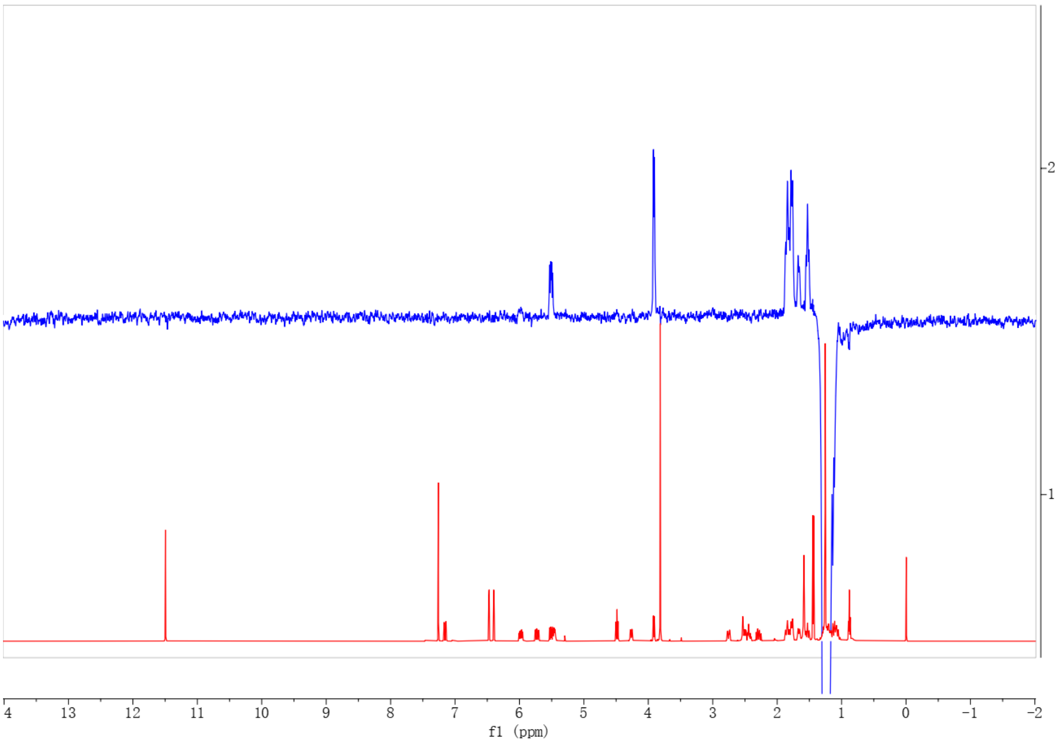

Figure S18. 1D NOE spectrum of compound **14**

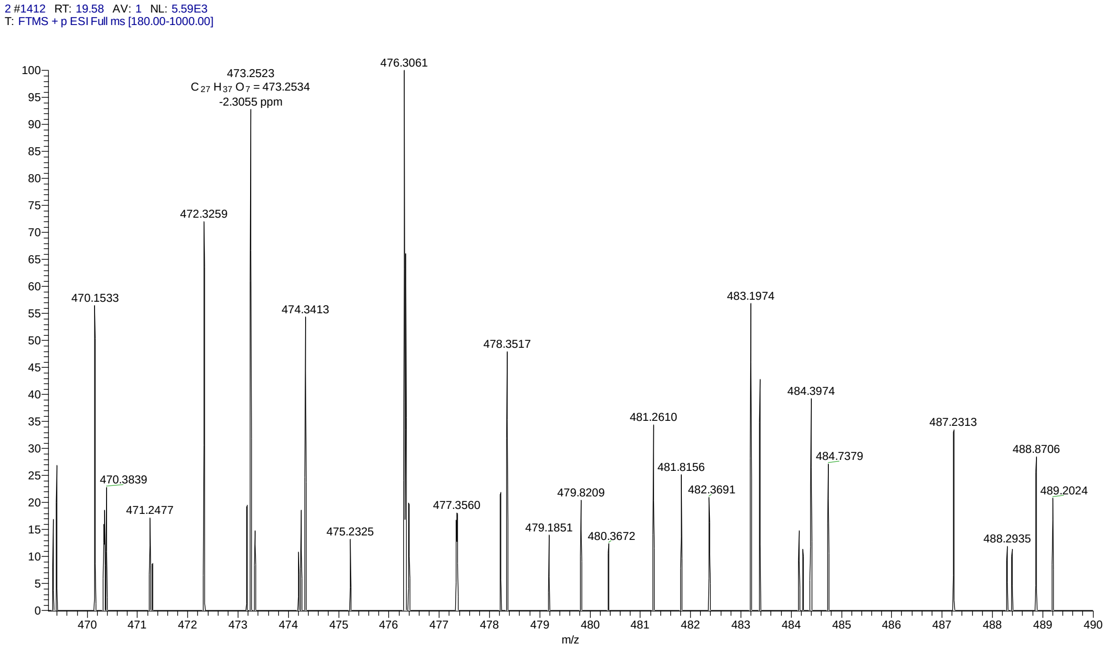

Figure S19. HR-ESI-MS spectrum of compound **14**.

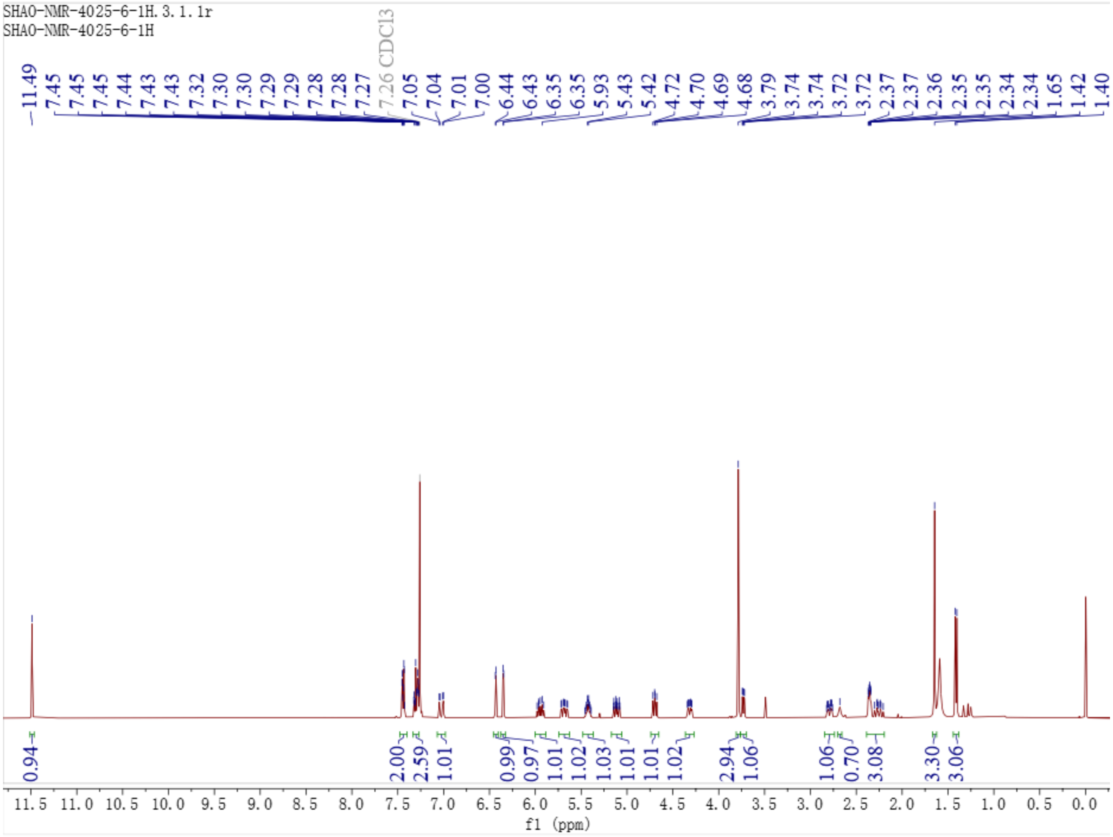

Figure S20. ^{1}H NMR (400 MHz, Chloroform-*d*) spectrum of compound **15**.

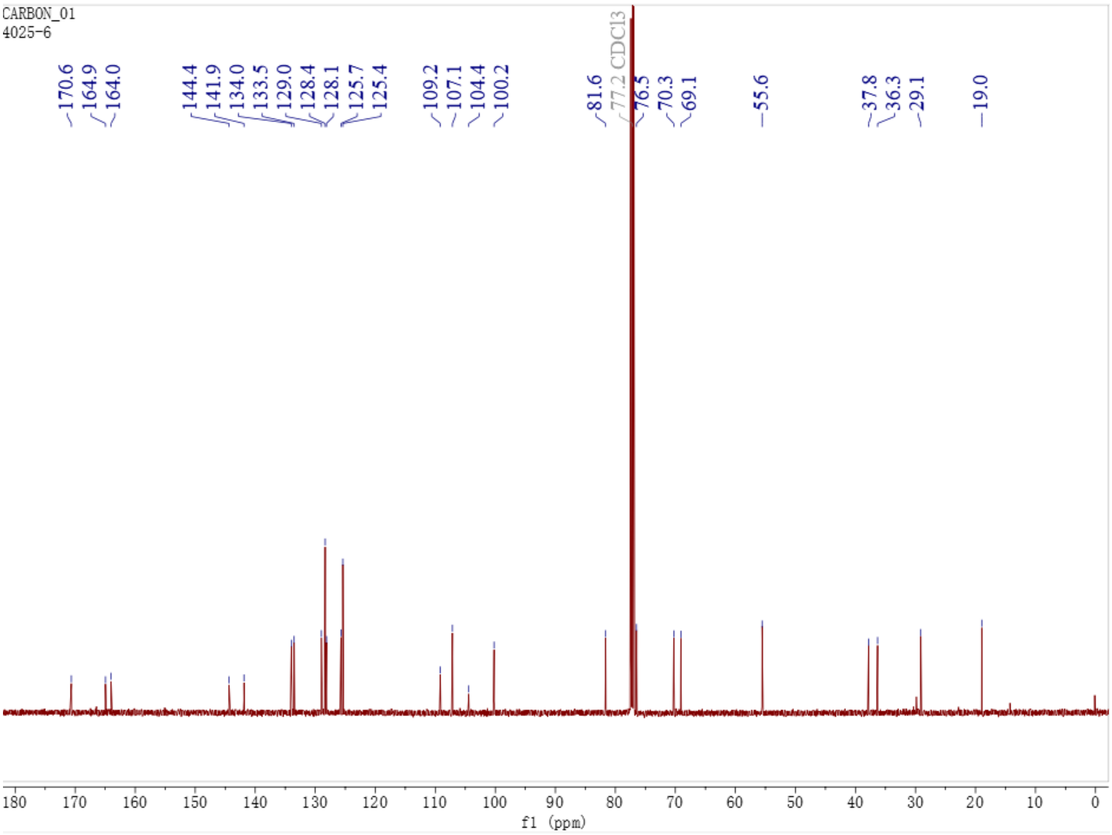

Figure S21. ^{13}C NMR (125MHz, Chloroform-*d*) spectrum of compound **15**.

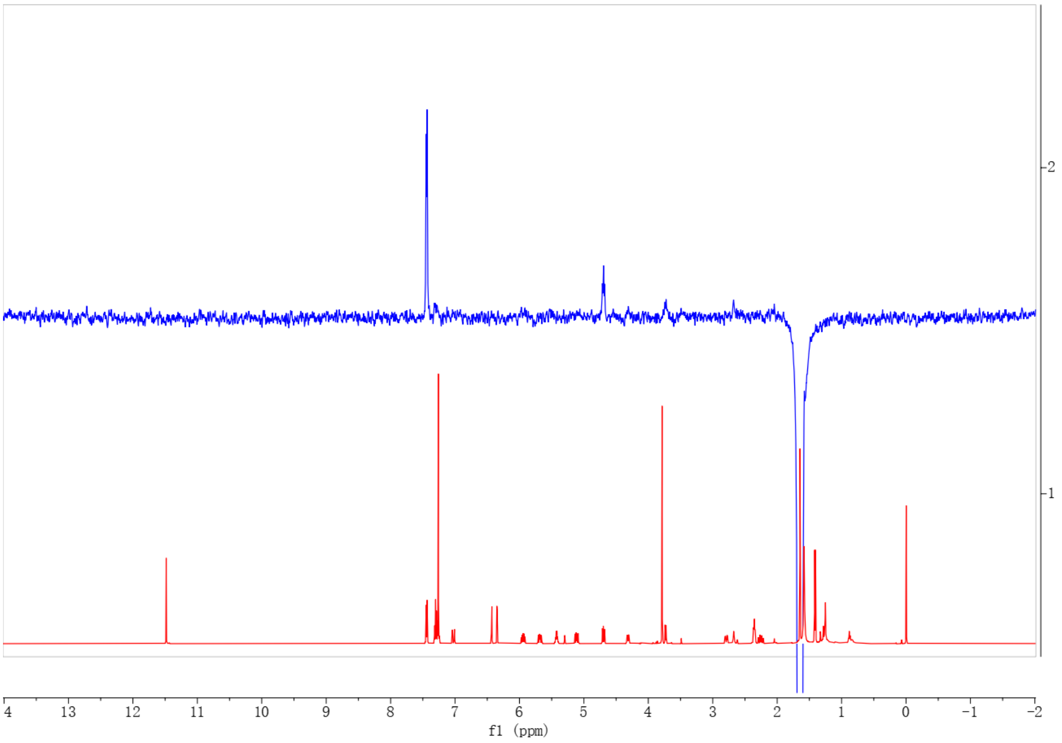

Figure S22. 1D NOE spectrum of compound **15**

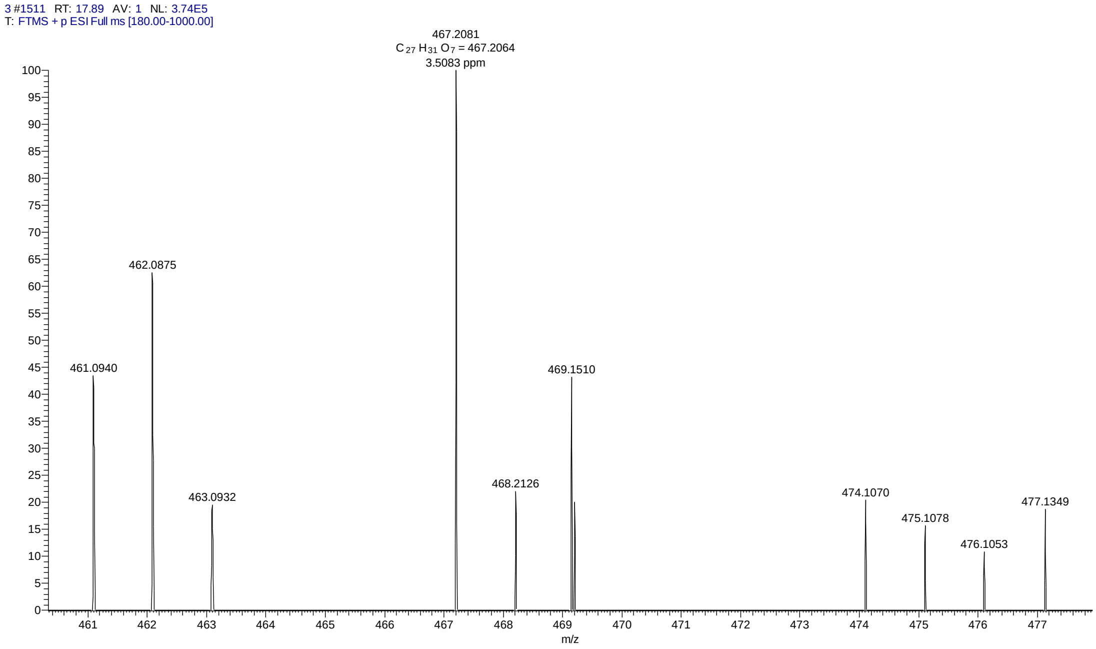

Figure S23. HR-ESI-MS spectrum of compound **15**.

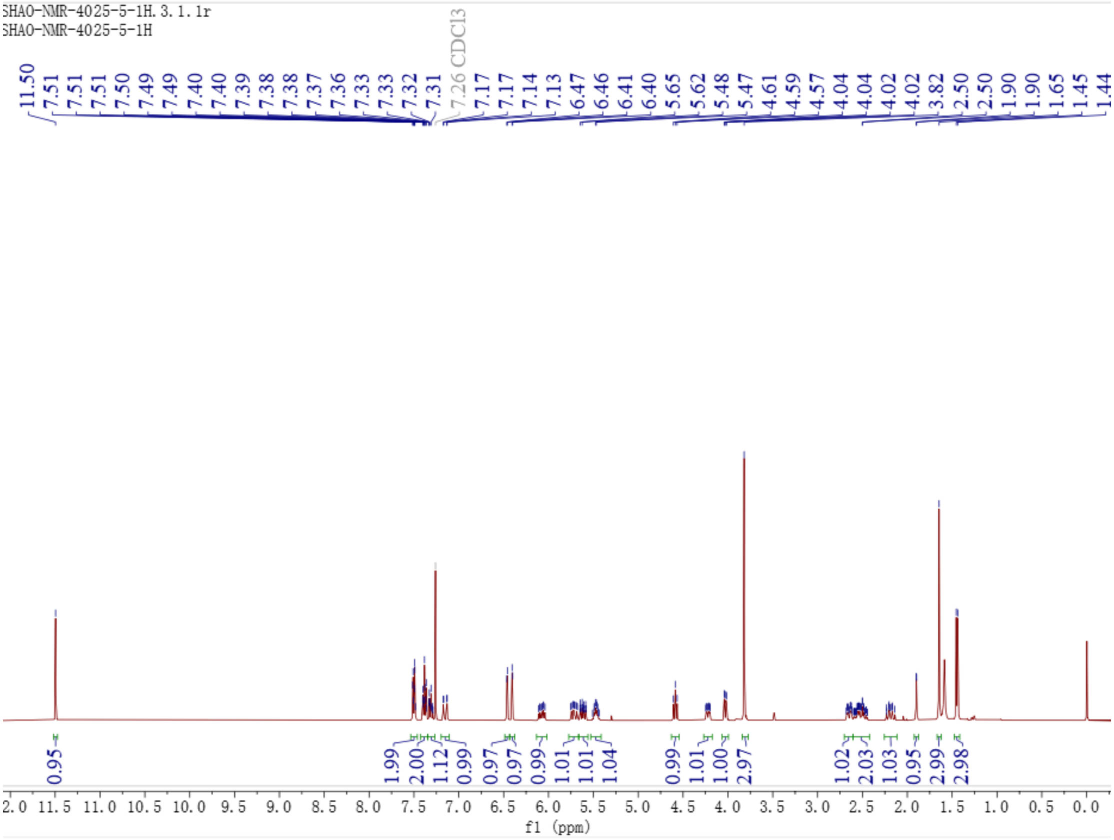

Figure S24. ^{1}H NMR (400 MHz, Chloroform-*d*) spectrum of compound **16**.

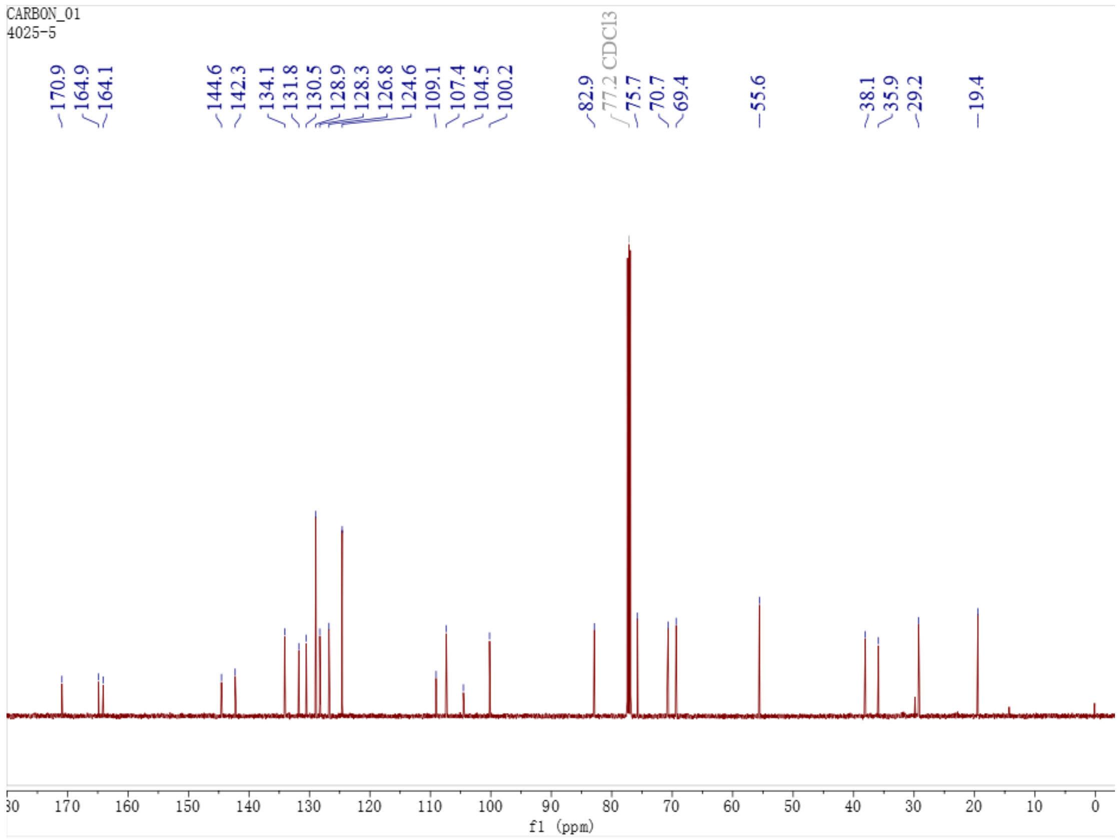

Figure S25. ^{13}C NMR (125 MHz, Chloroform-*d*) spectrum of compound **16**.

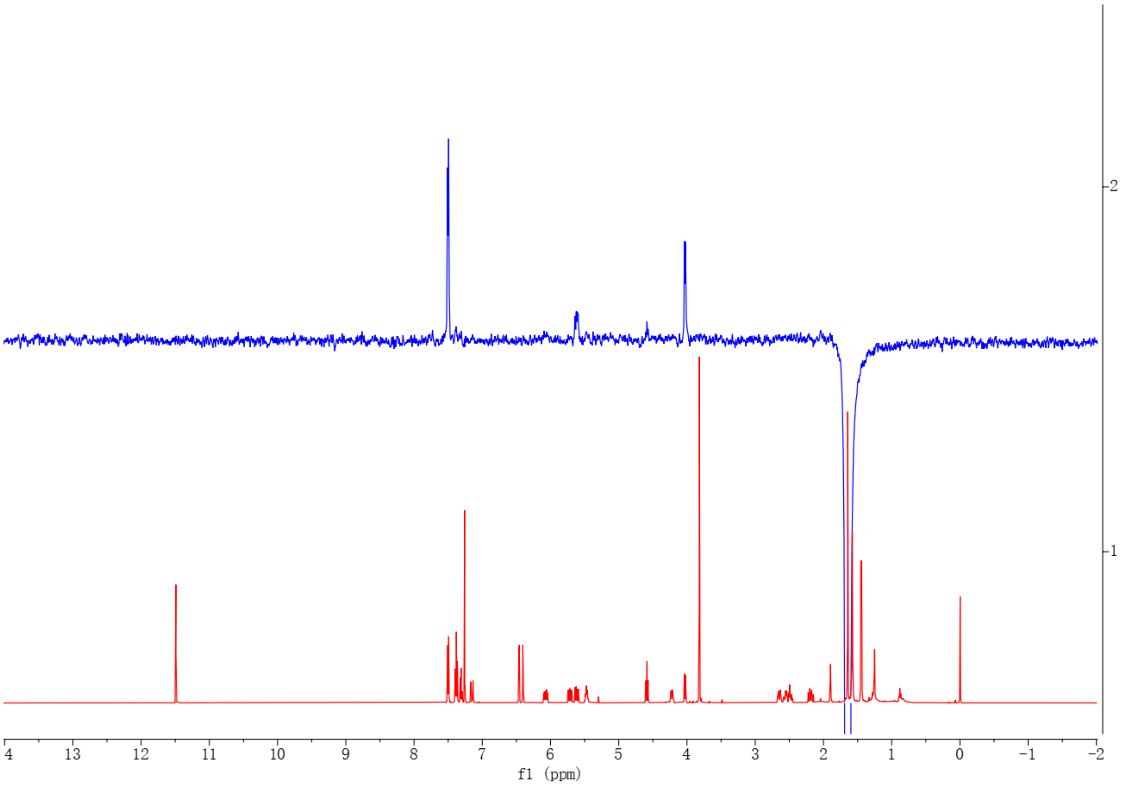

Figure S26. 1D NOE spectrum of compound **16**

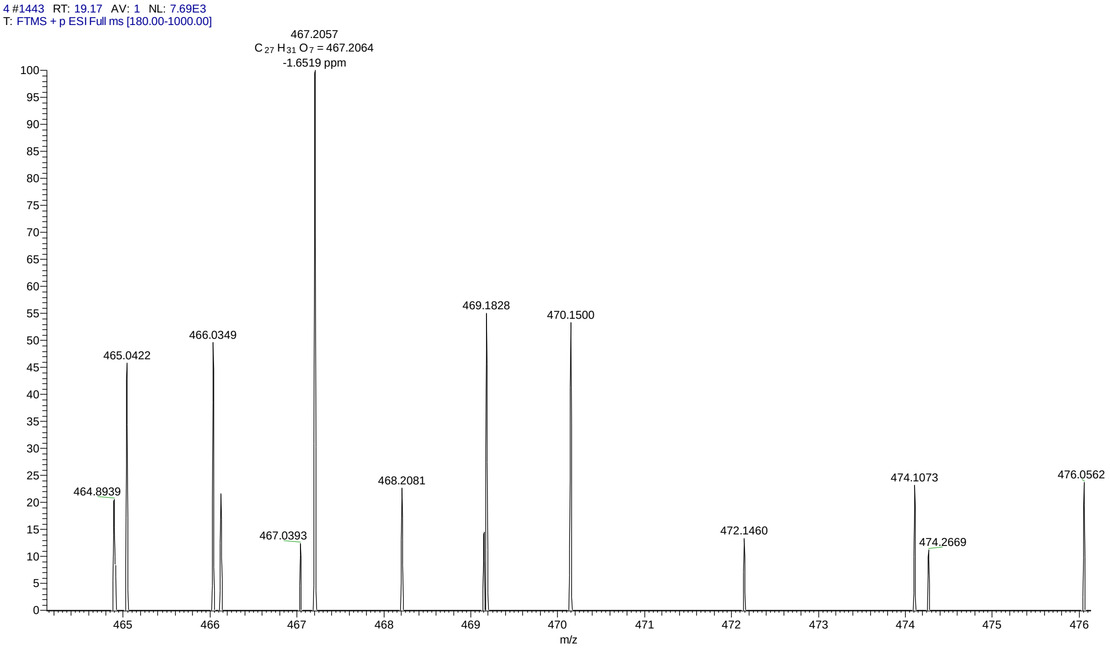

Figure S27. HR-ESI-MS spectrum of compound **16**.

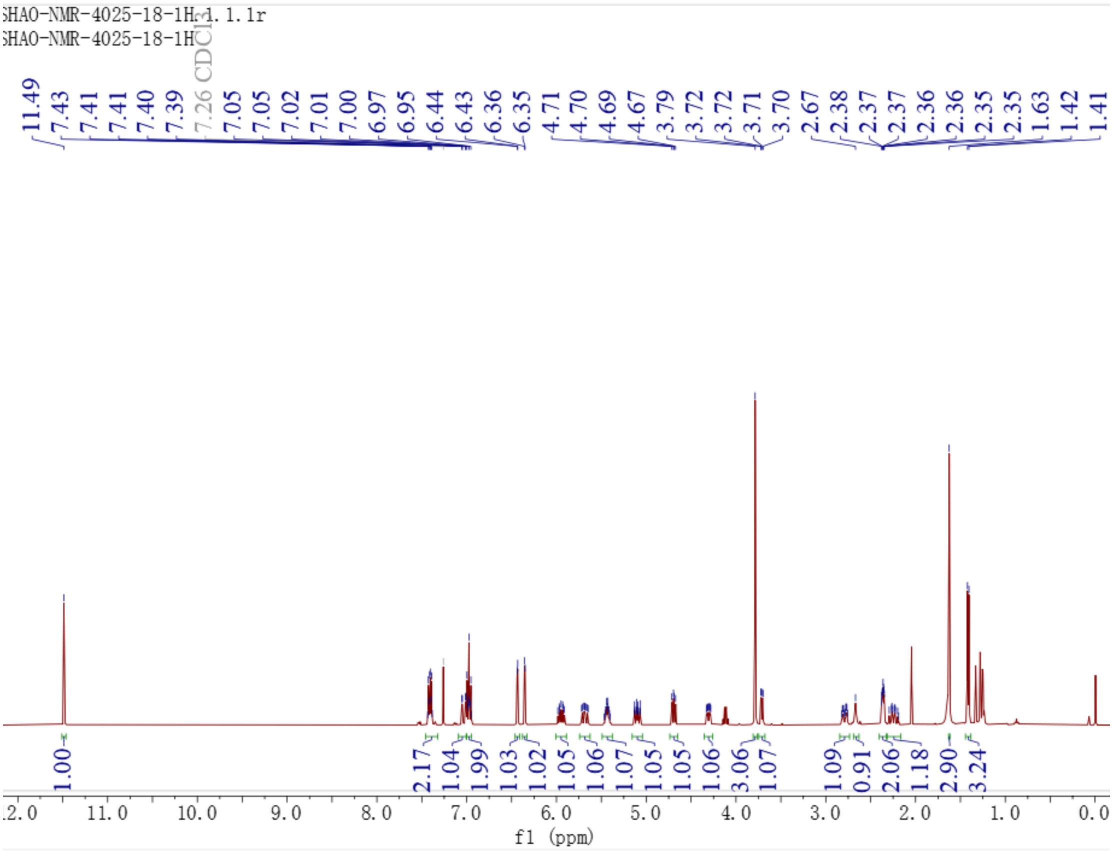

Figure S28. ^{1}H NMR (400 MHz, Chloroform-*d*) spectrum of compound **17**.

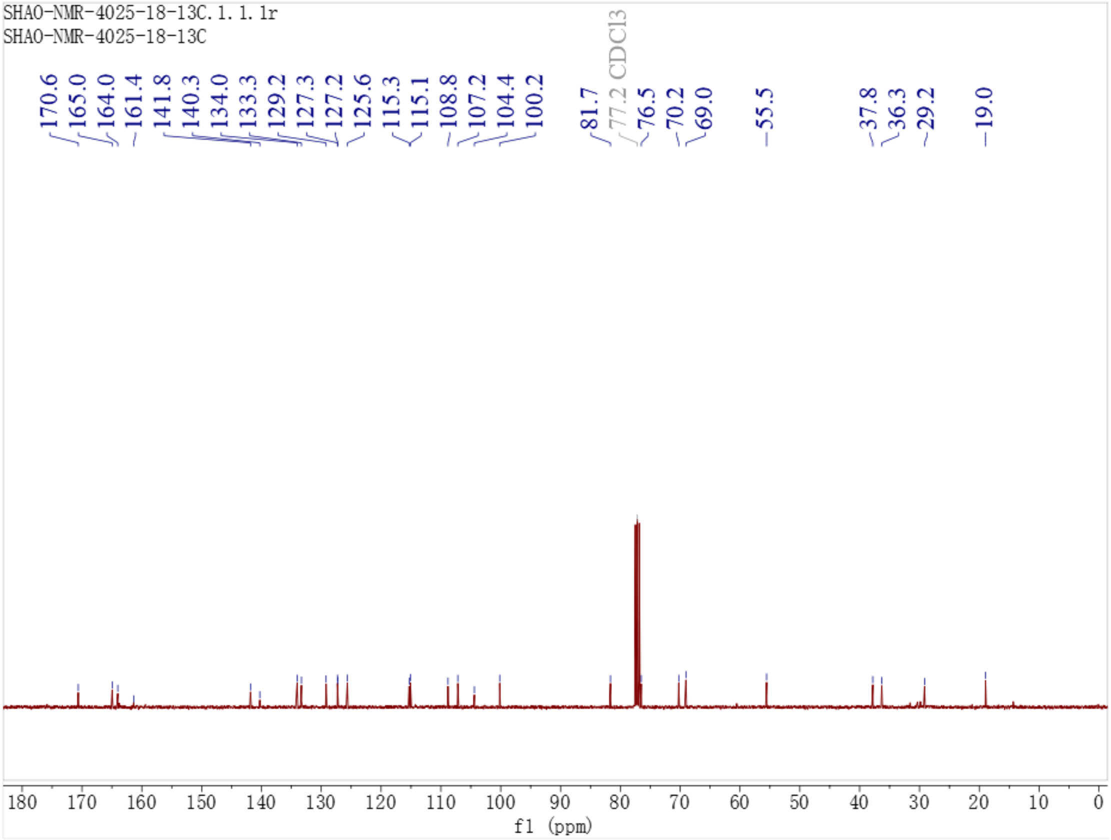

Figure S29. ^{13}C NMR (100 MHz, Chloroform-*d*) spectrum of compound **17**.

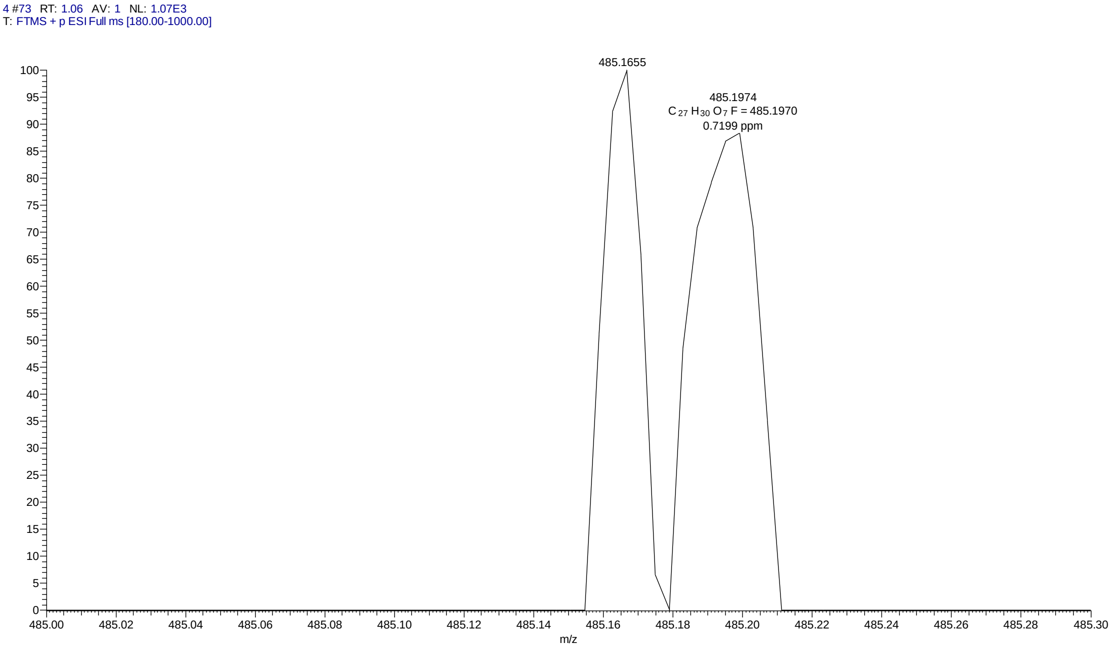

Figure S30. HR-ESI-MS spectrum of compound **17**.

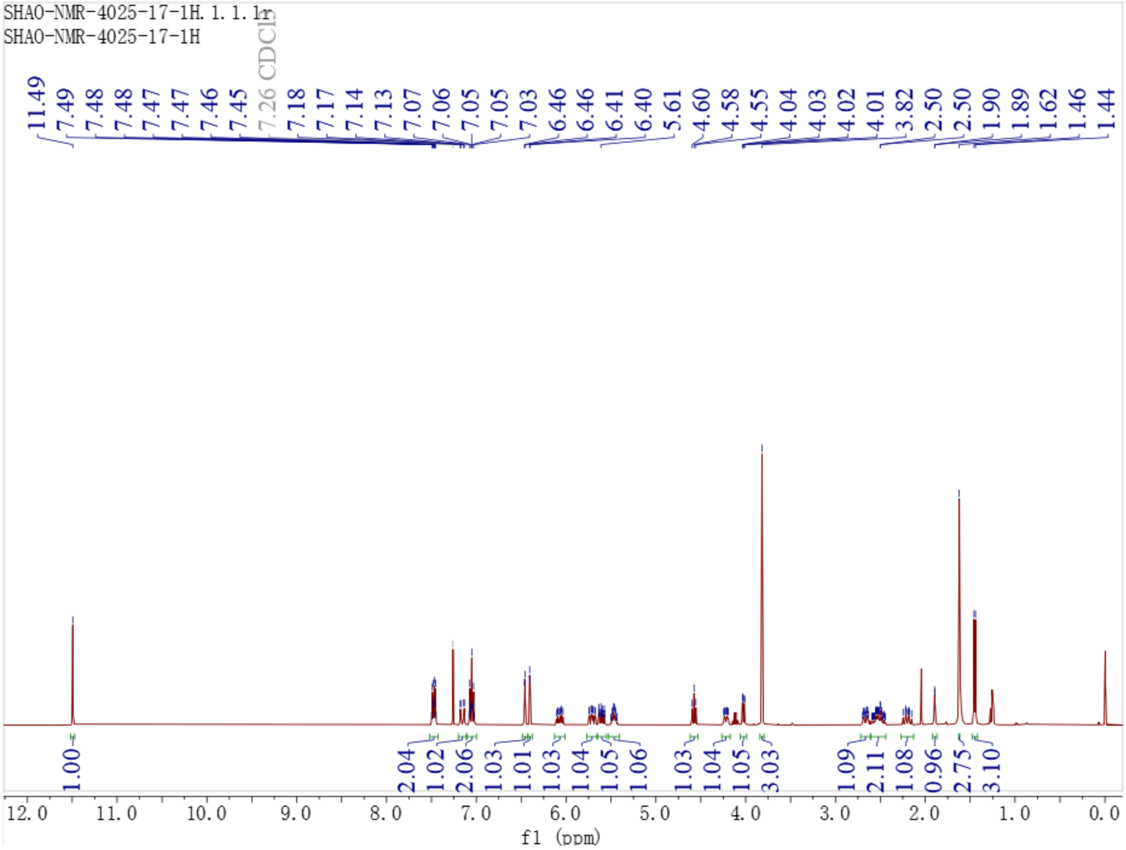

Figure S31. ^{1}H NMR (400 MHz, Chloroform-*d*) spectrum of compound **18**.

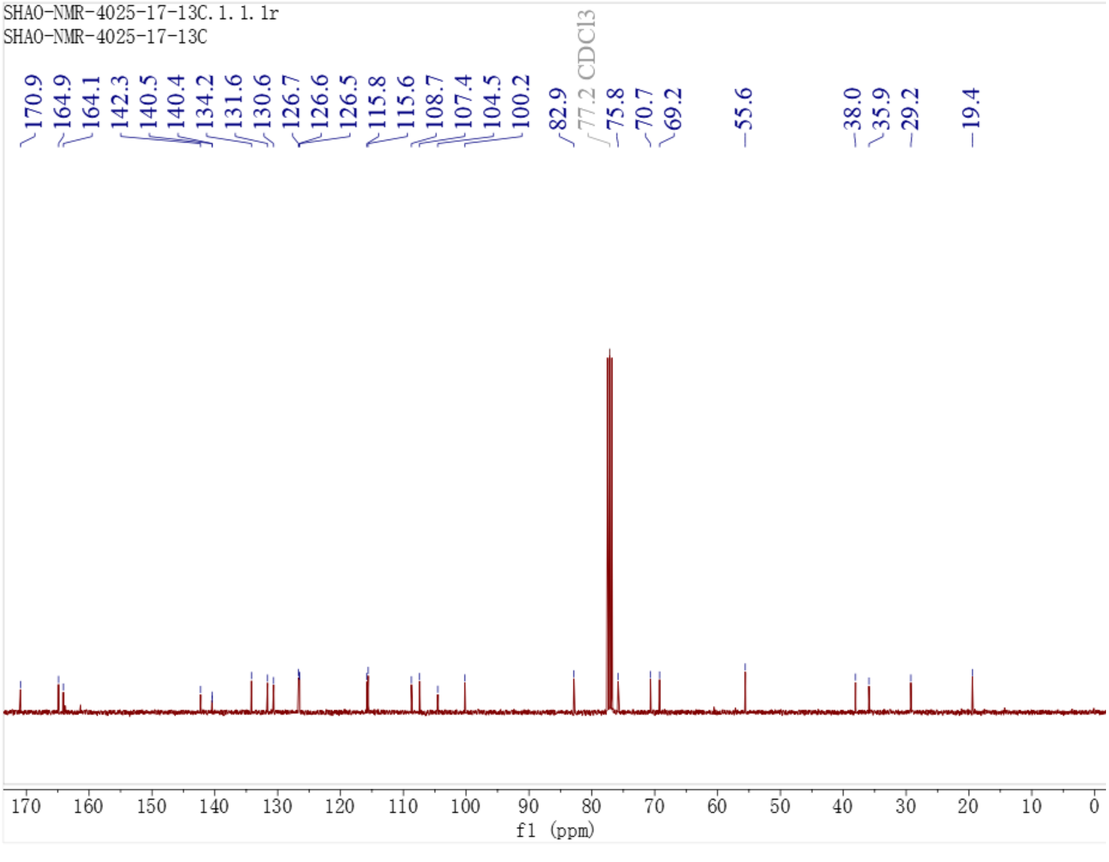

Figure S32. ^{13}C NMR (100 MHz, Chloroform-*d*) spectrum of compound **18**.

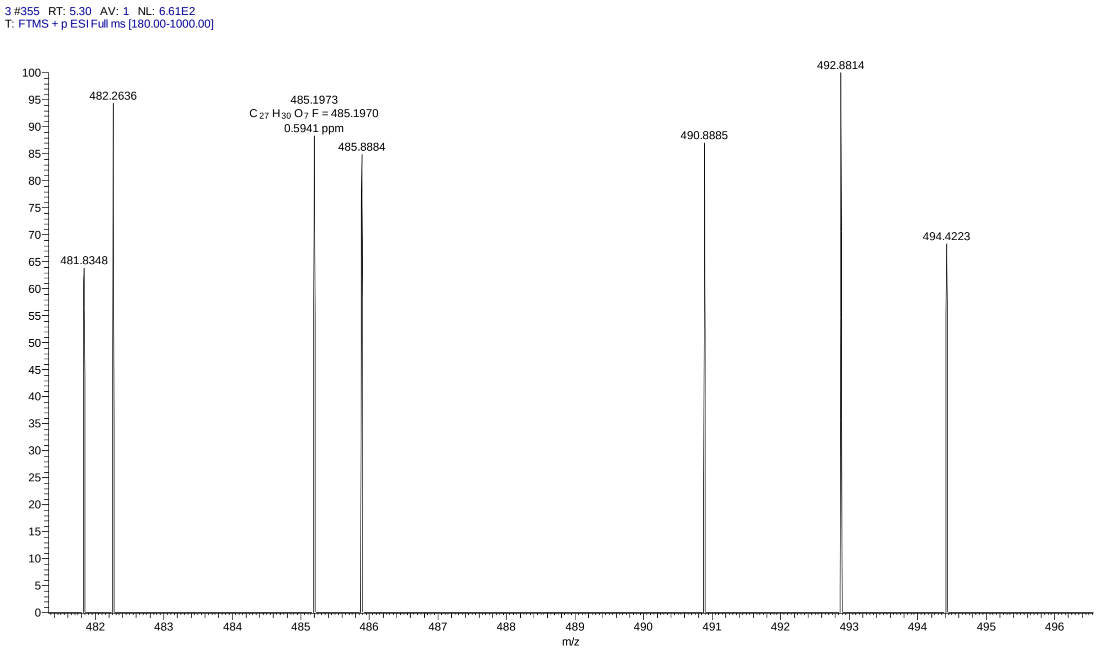

Figure S33. HR-ESI-MS spectrum of compound **18**.

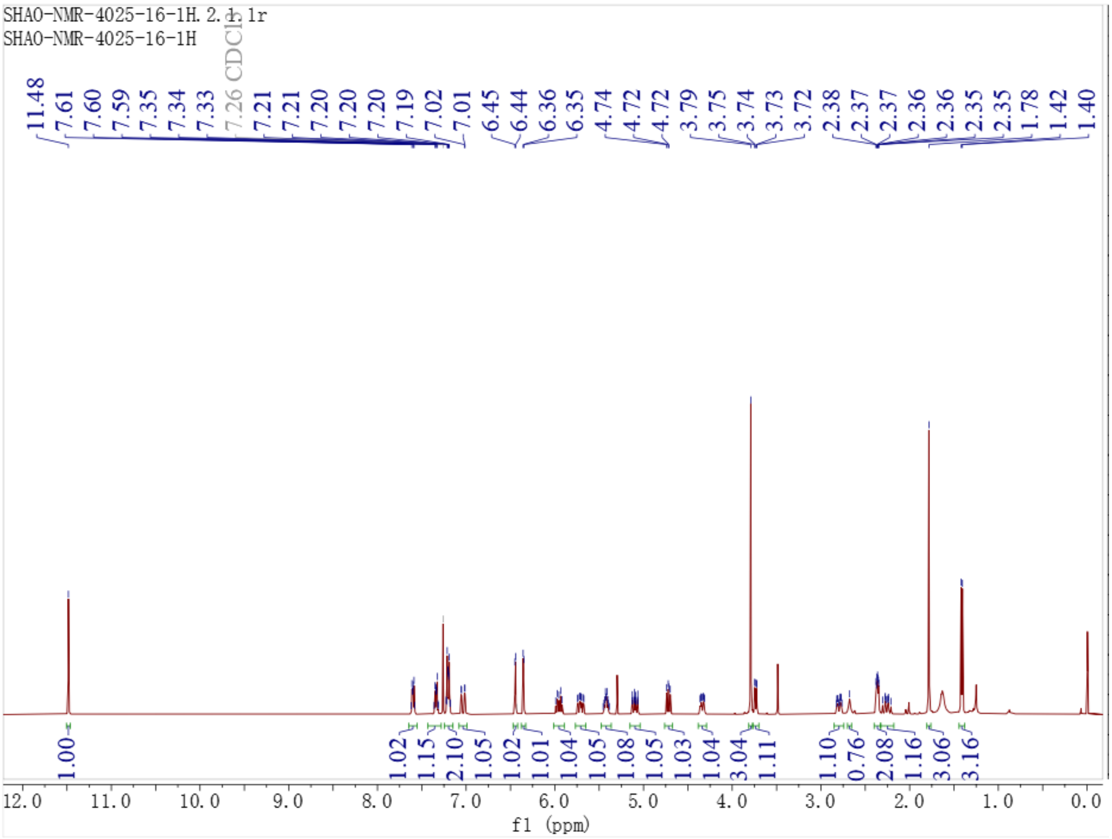

Figure S34. ^{1}H NMR (400 MHz, Chloroform-*d*) spectrum of compound **19**.

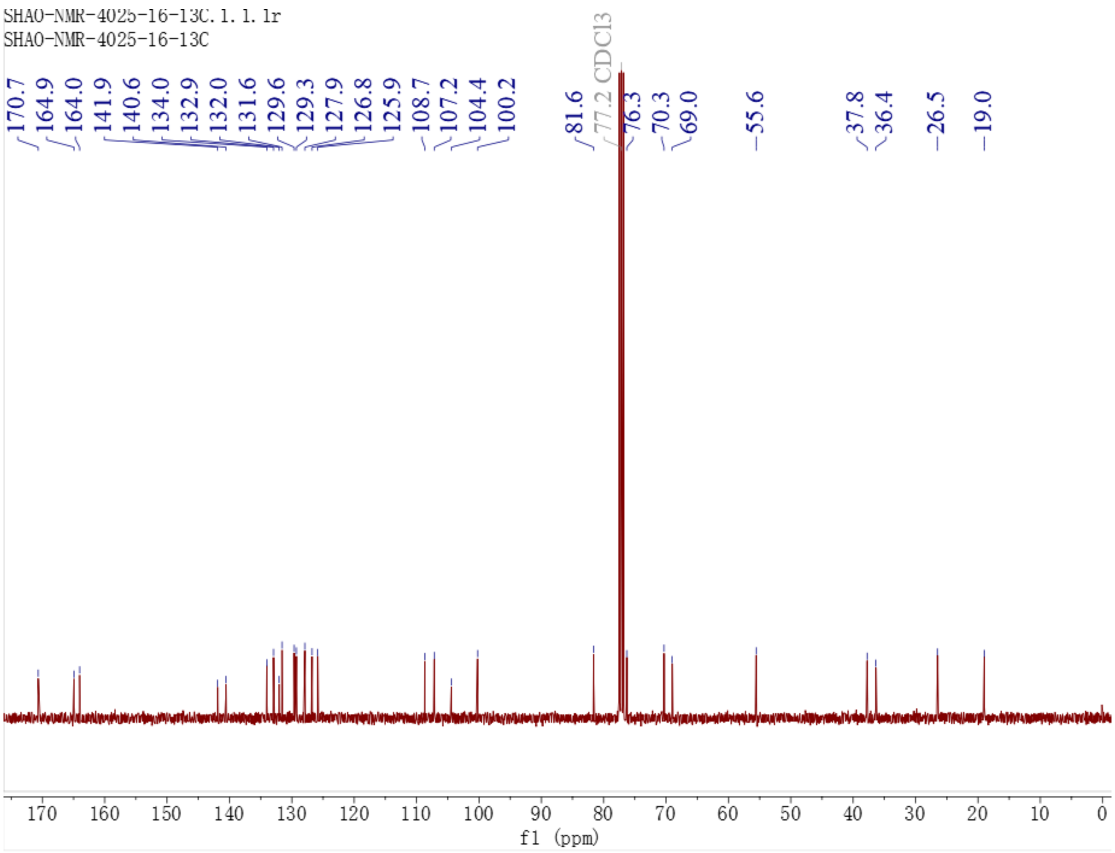

Figure S35. ^{13}C NMR (100 MHz, Chloroform-*d*) spectrum of compound **19**.

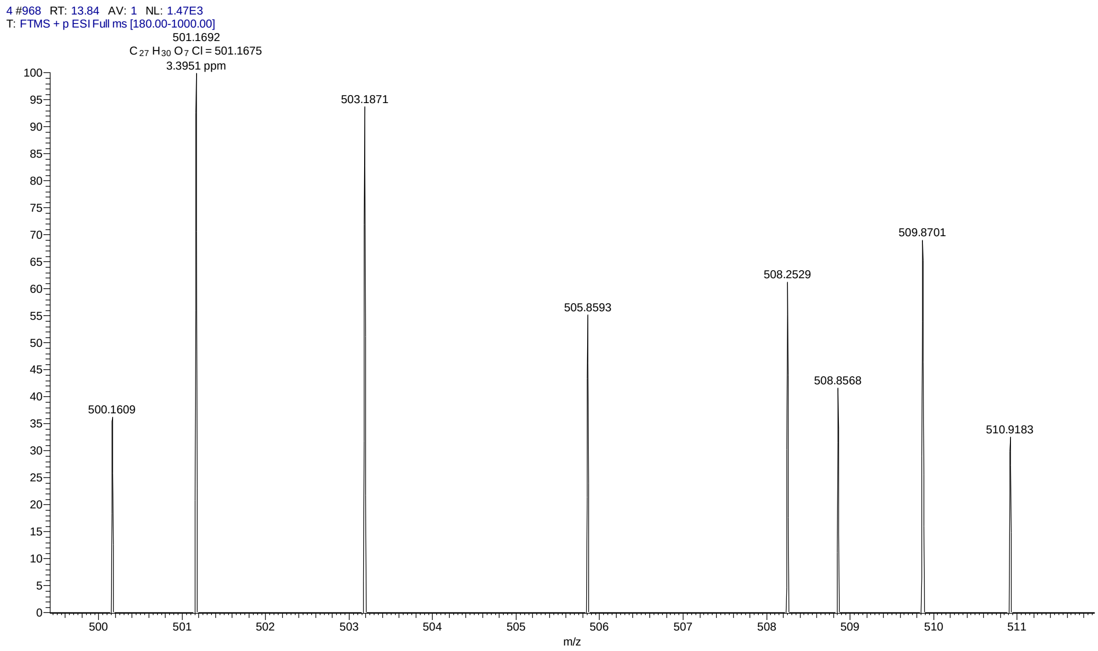

Figure S36. HR-ESI-MS spectrum of compound **19**.

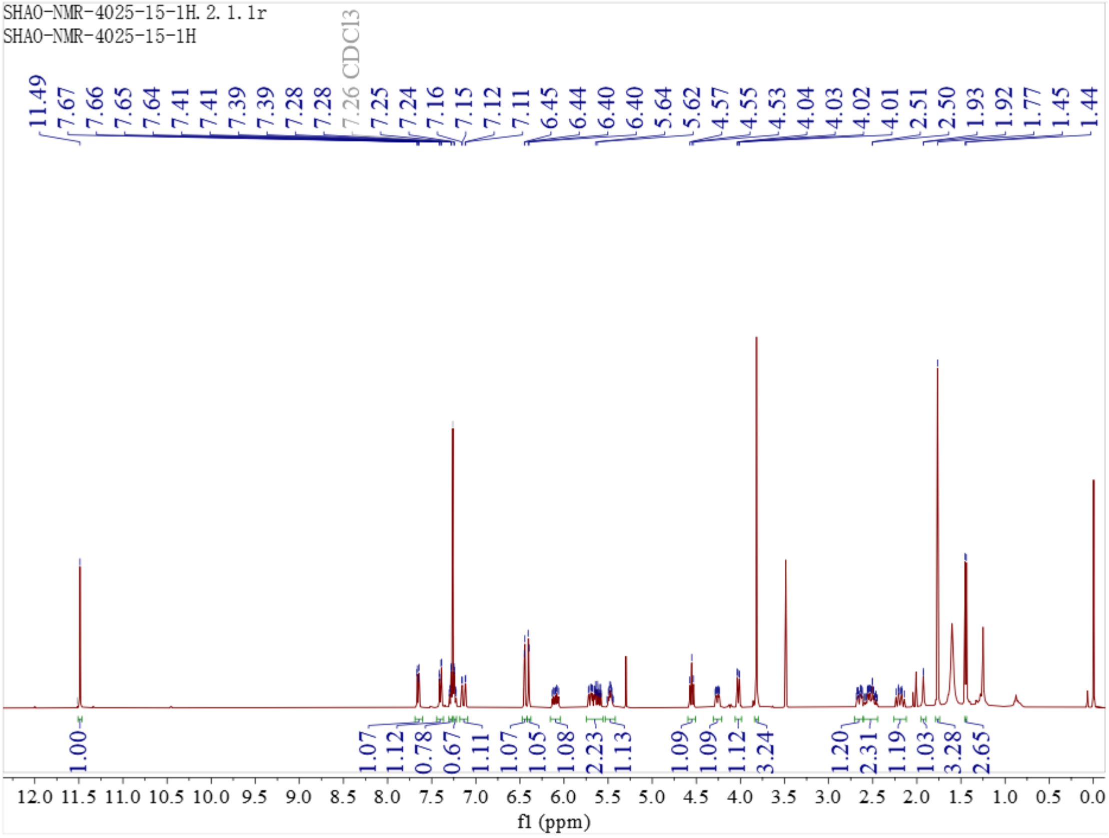

Figure S37. ^{1}H NMR (400 MHz, Chloroform-*d*) spectrum of compound **20**.

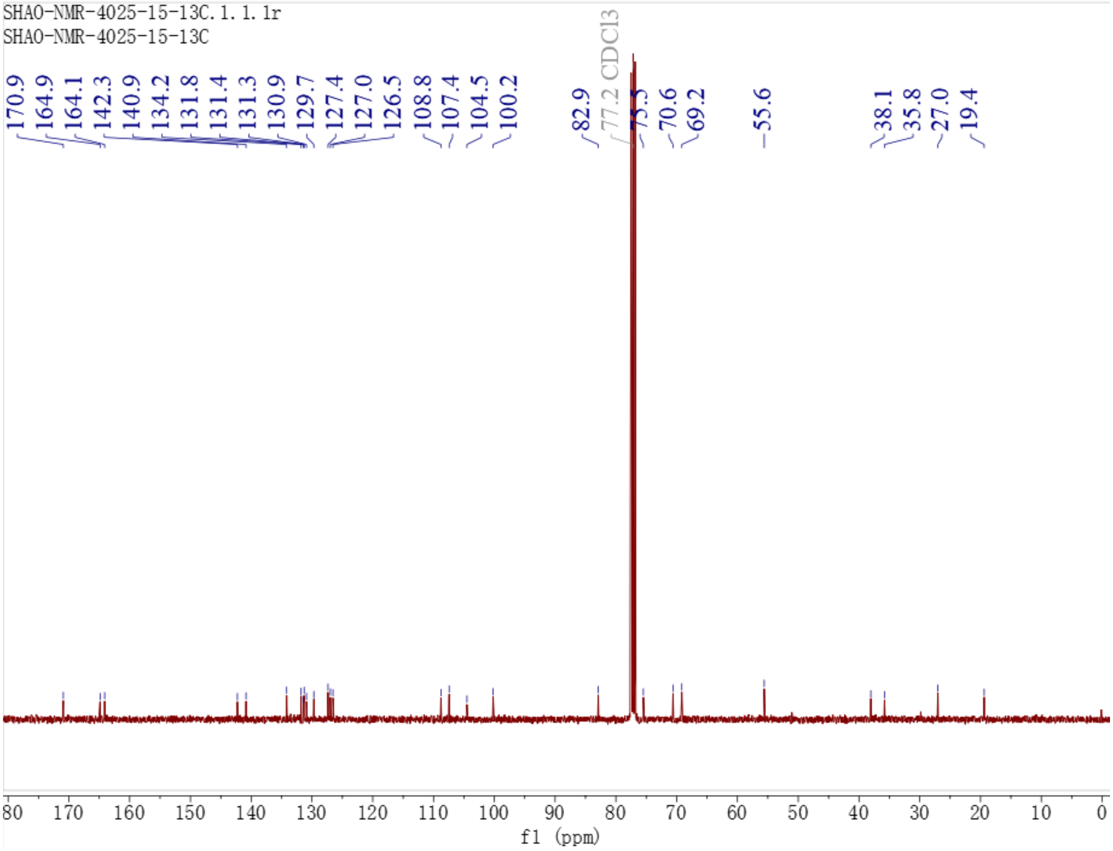

Figure S38. ^{13}C NMR (100 MHz, Chloroform-*d*) spectrum of compound **20**.

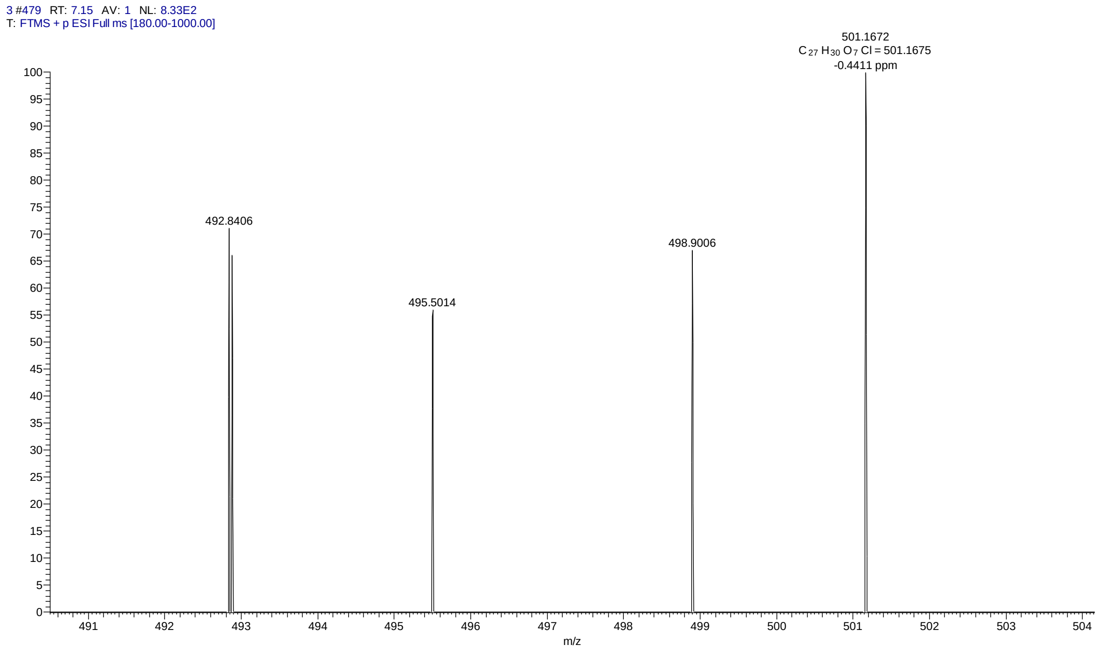

Figure S39. HR-ESI-MS spectrum of compound **20**.

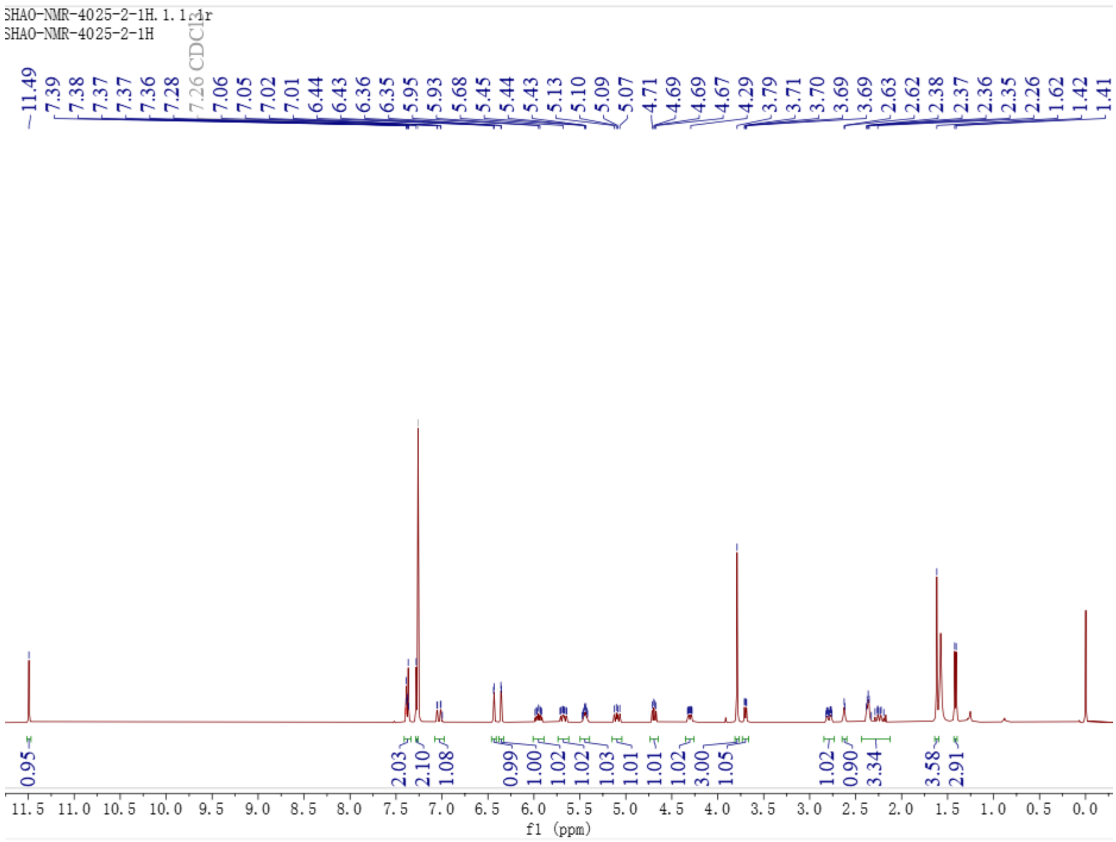

Figure S40. ^{1}H NMR (400 MHz, Chloroform-*d*) spectrum of compound **21**

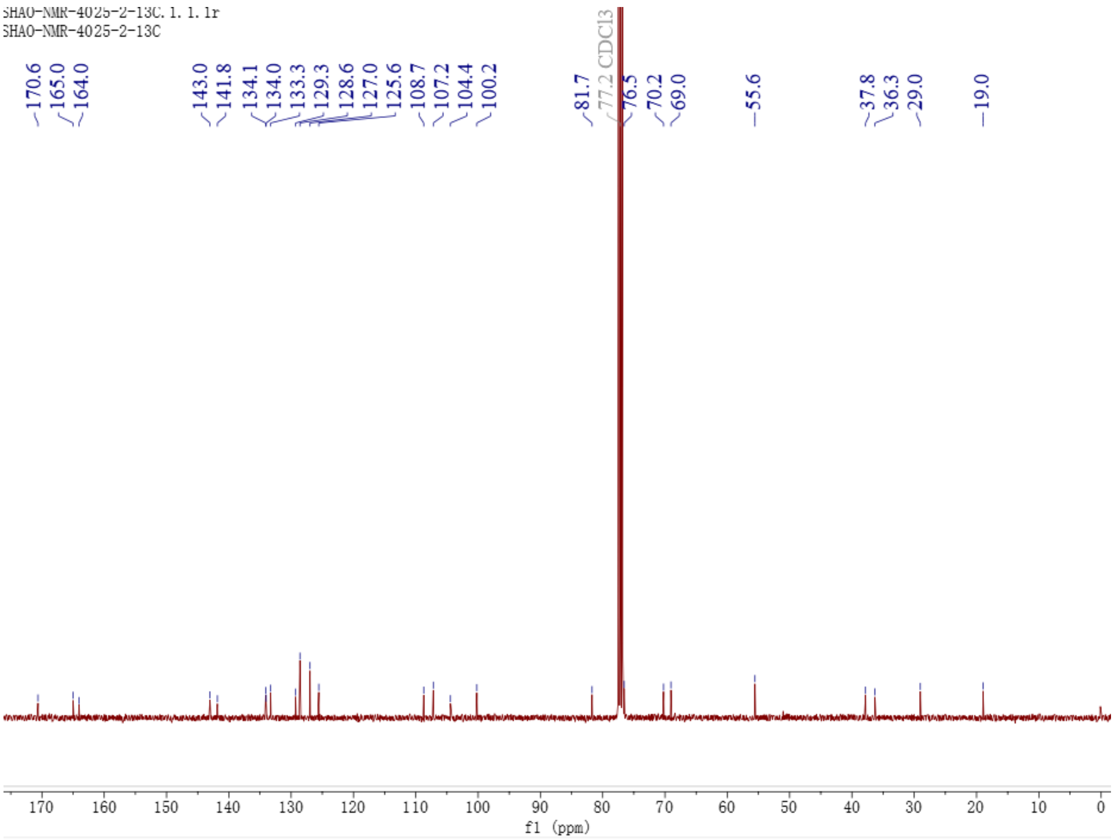

Figure S41. ^{13}C NMR (100 MHz, Chloroform-*d*) spectrum of compound **21**.

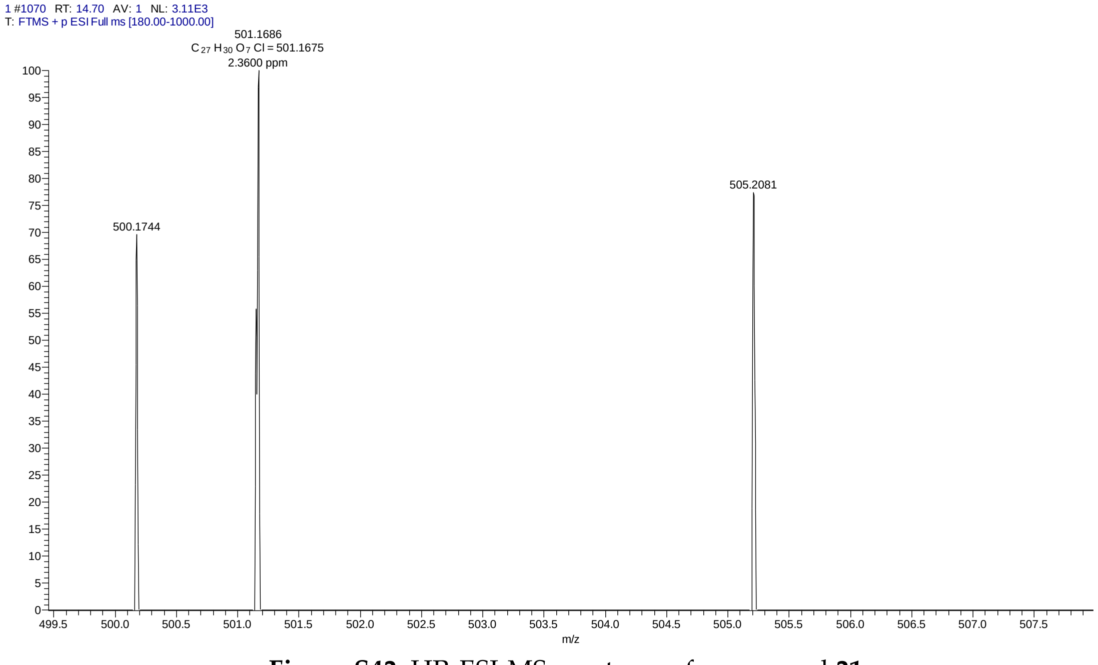

Figure S42. HR-ESI-MS spectrum of compound **21**.

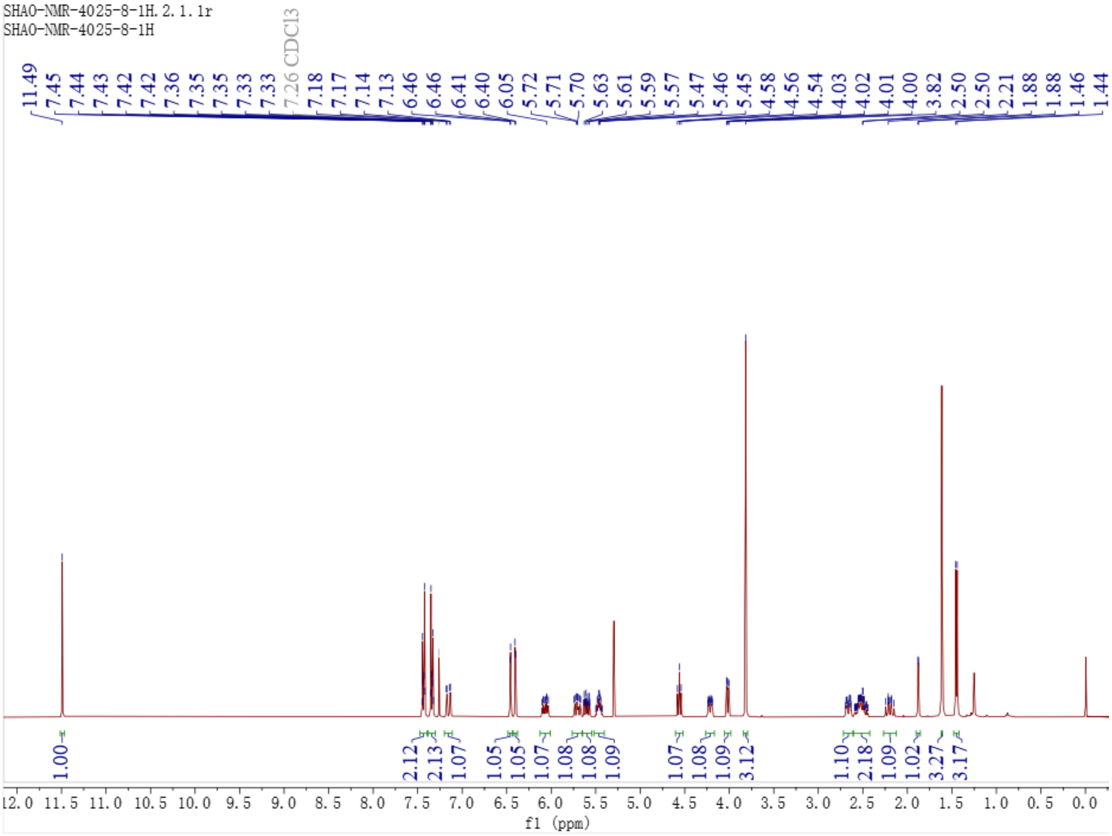

Figure S43. ^{1}H NMR (400 MHz, Chloroform-*d*) spectrum of compound **22.**

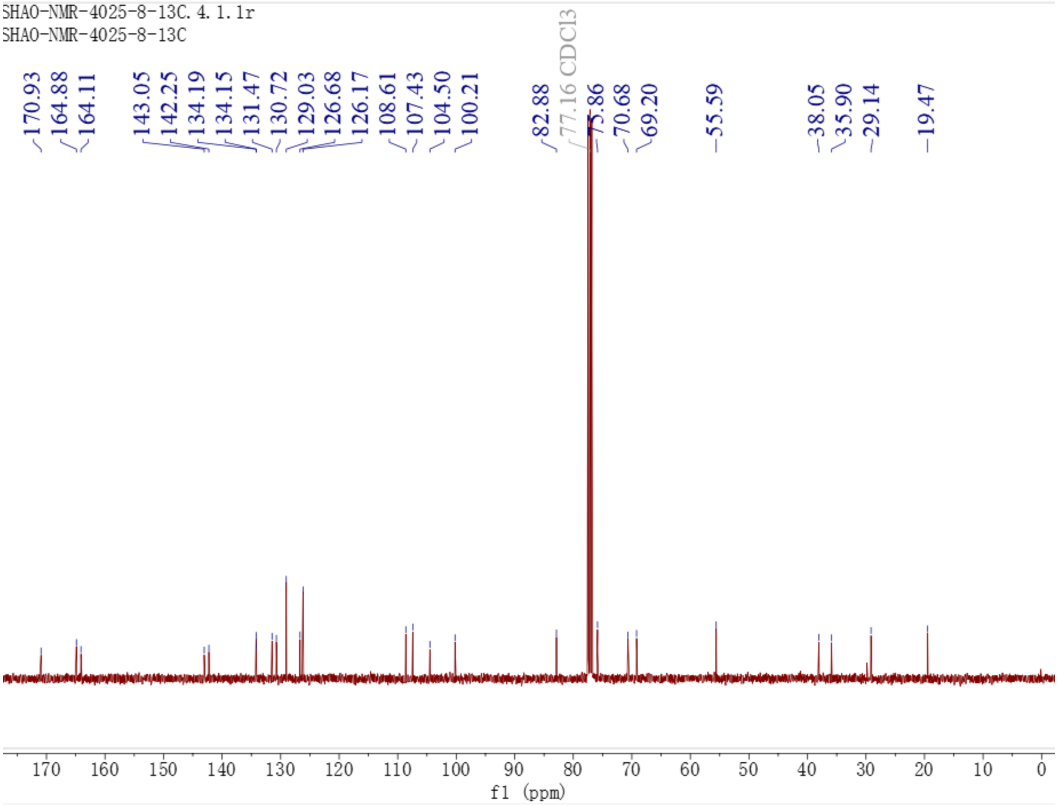

Figure S44. ^{13}C NMR (100 MHz, Chloroform-*d*) spectrum of compound **22**.

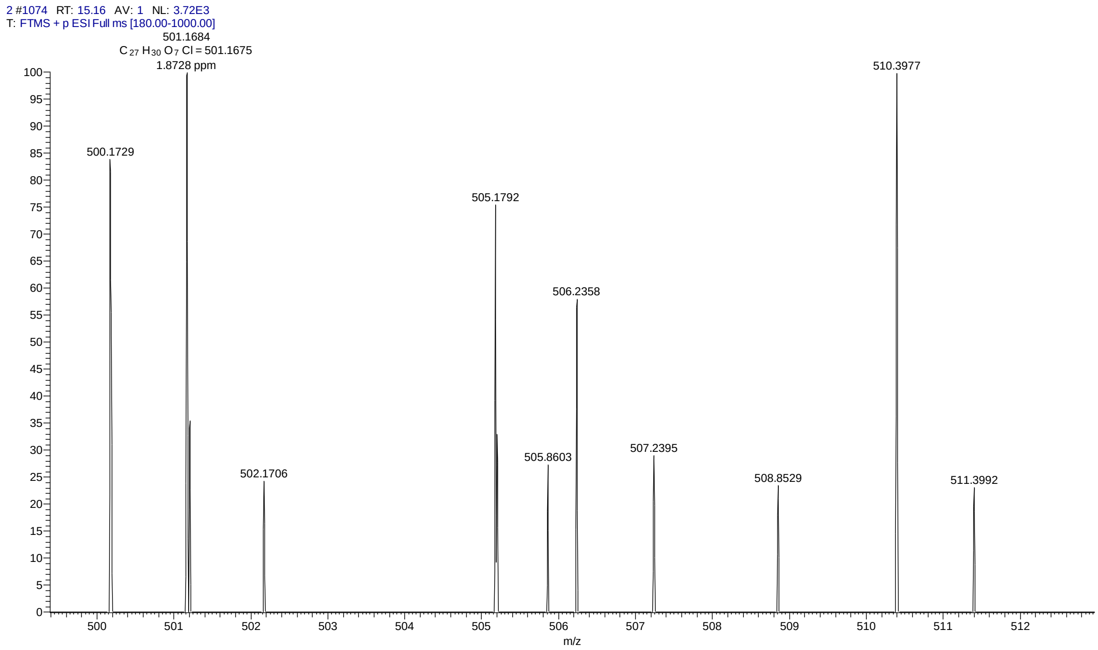

Figure S45. HR-ESI-MS spectrum of compound **22**.

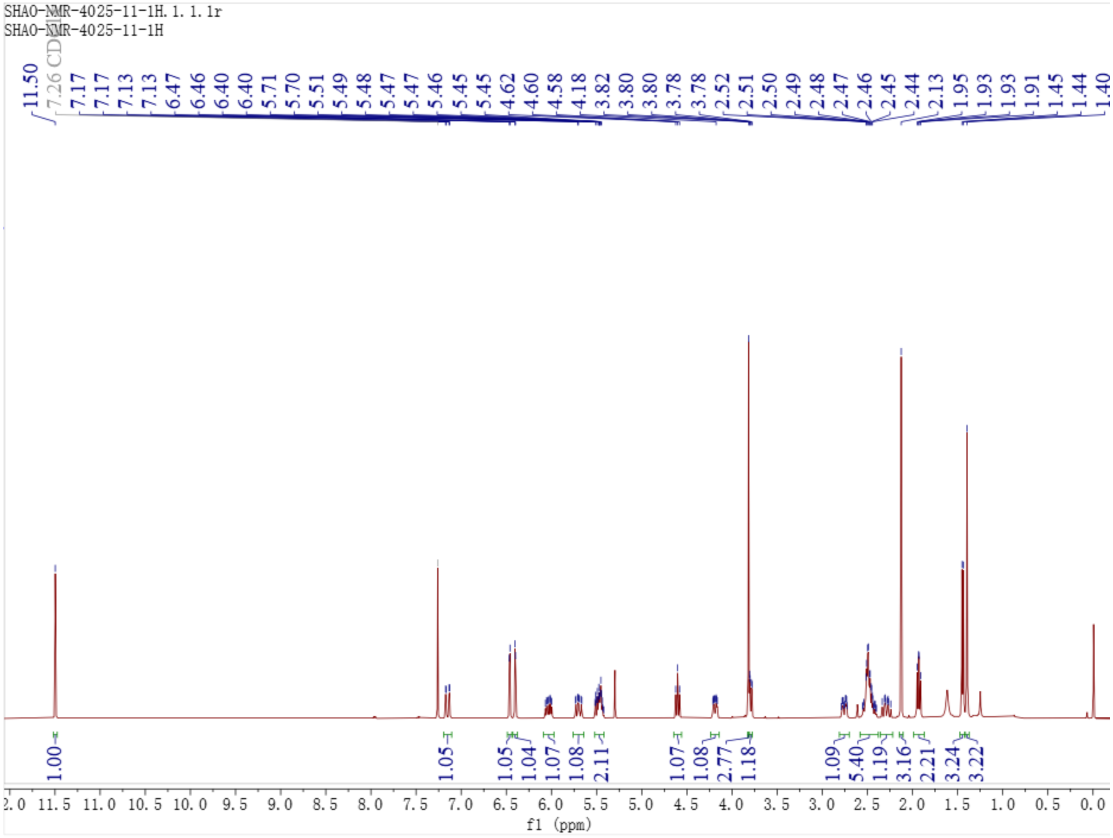

Fgure S46. ^{1}H NMR (400 MHz, Chloroform-*d*) spectrum of compound **23**.

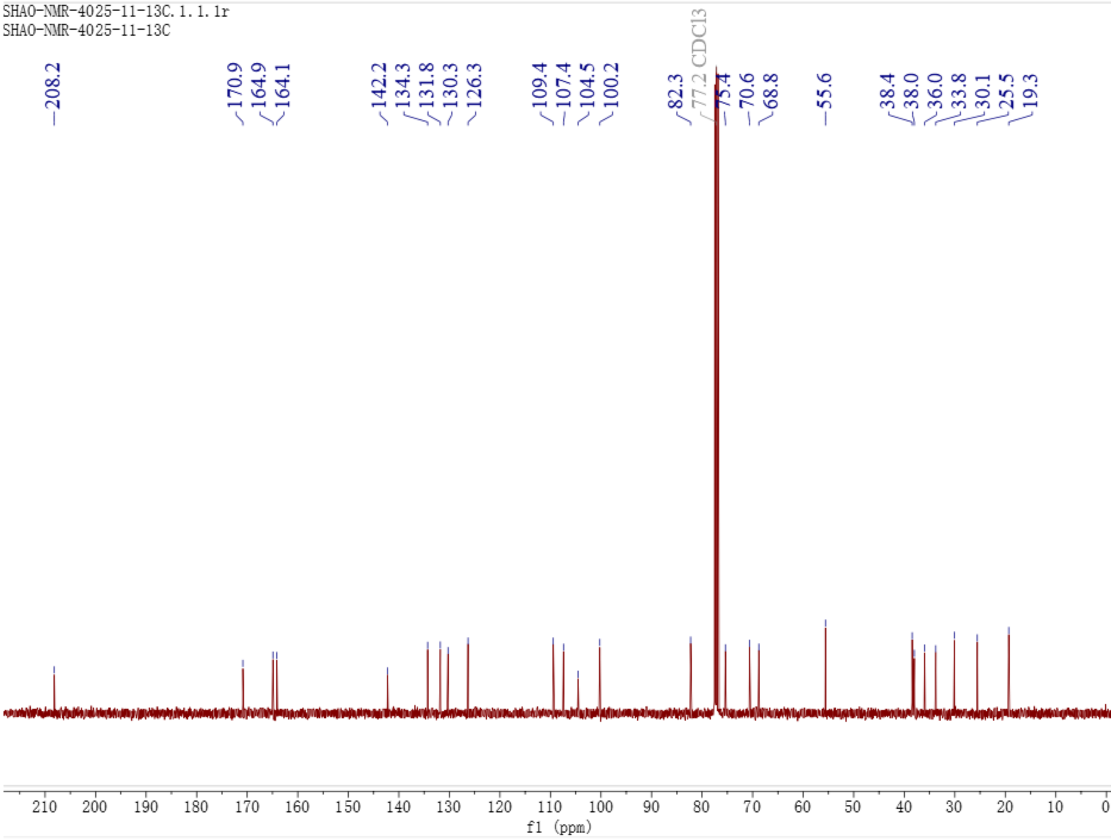

Figure S47. ^{13}C NMR (100 MHz, Chloroform-*d*) spectrum of compound **23**.

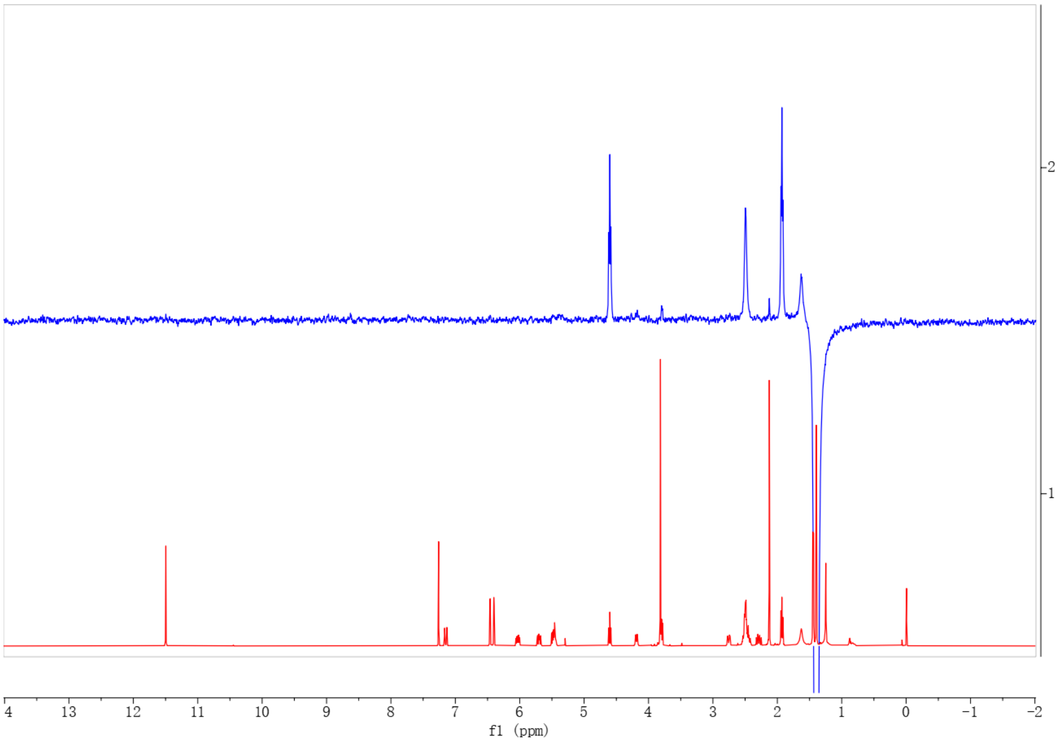

Figure S48. 1D NOE spectrum of compound **23**

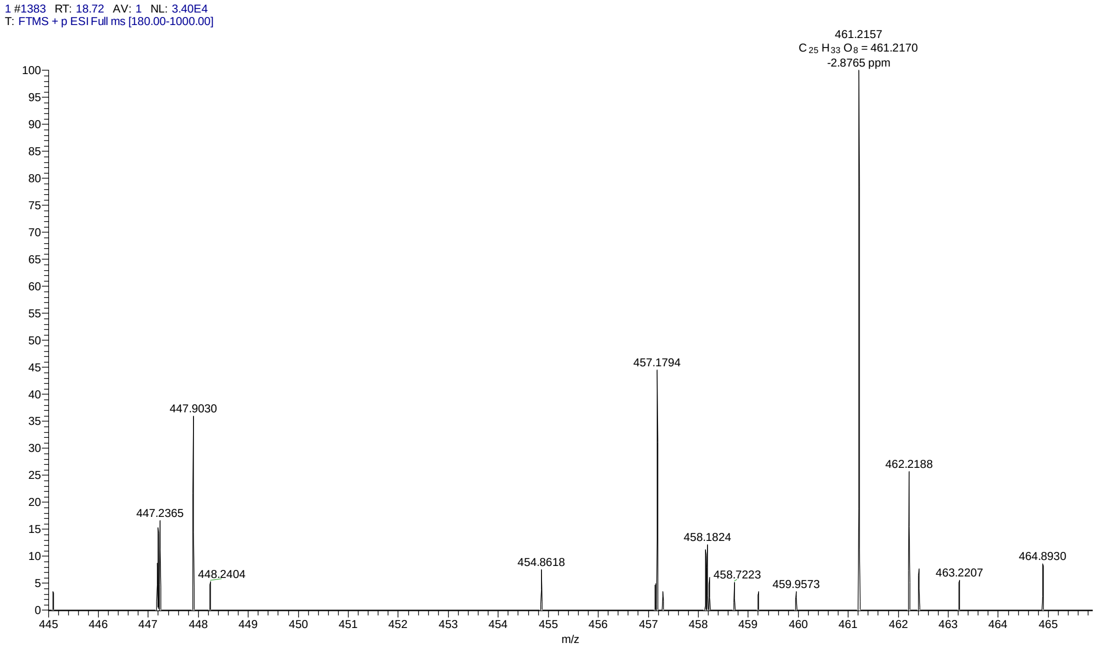

Figure S49. HR-ESI-MS spectrum of compound **23**.

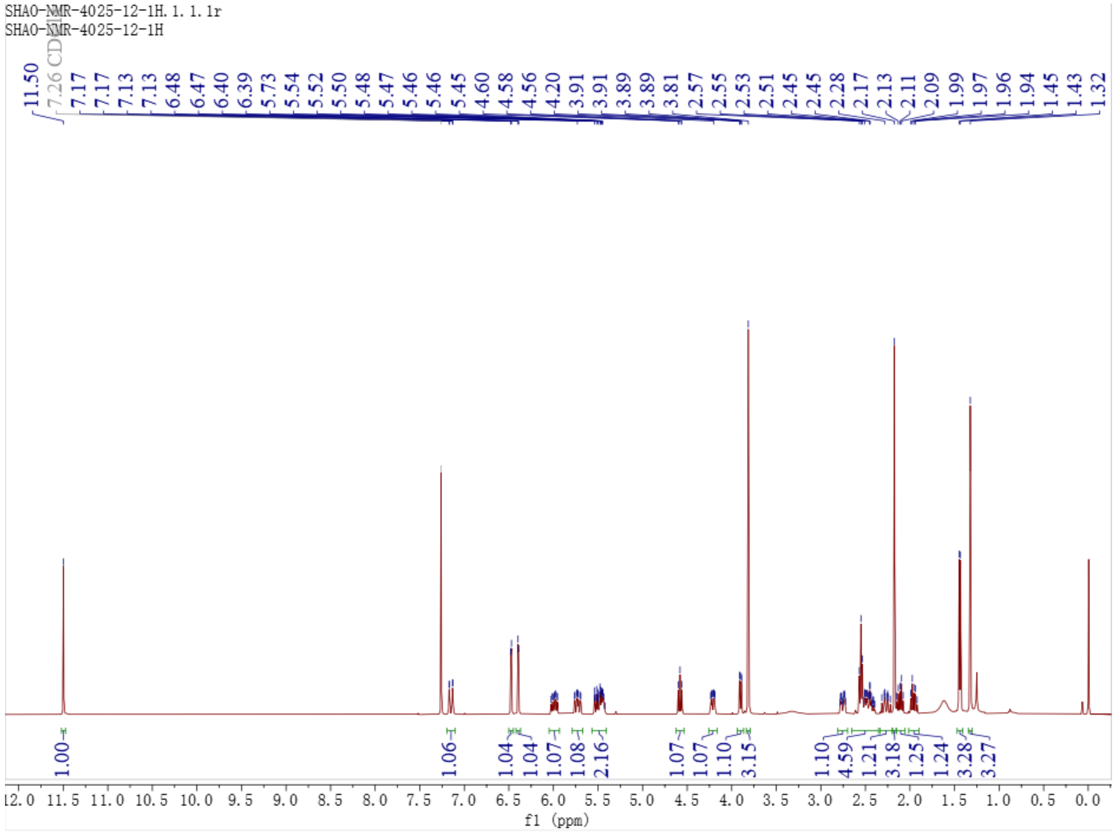

Figure S50. ^{1}H NMR (400 MHz, Chloroform-*d*) spectrum of compound **24**.

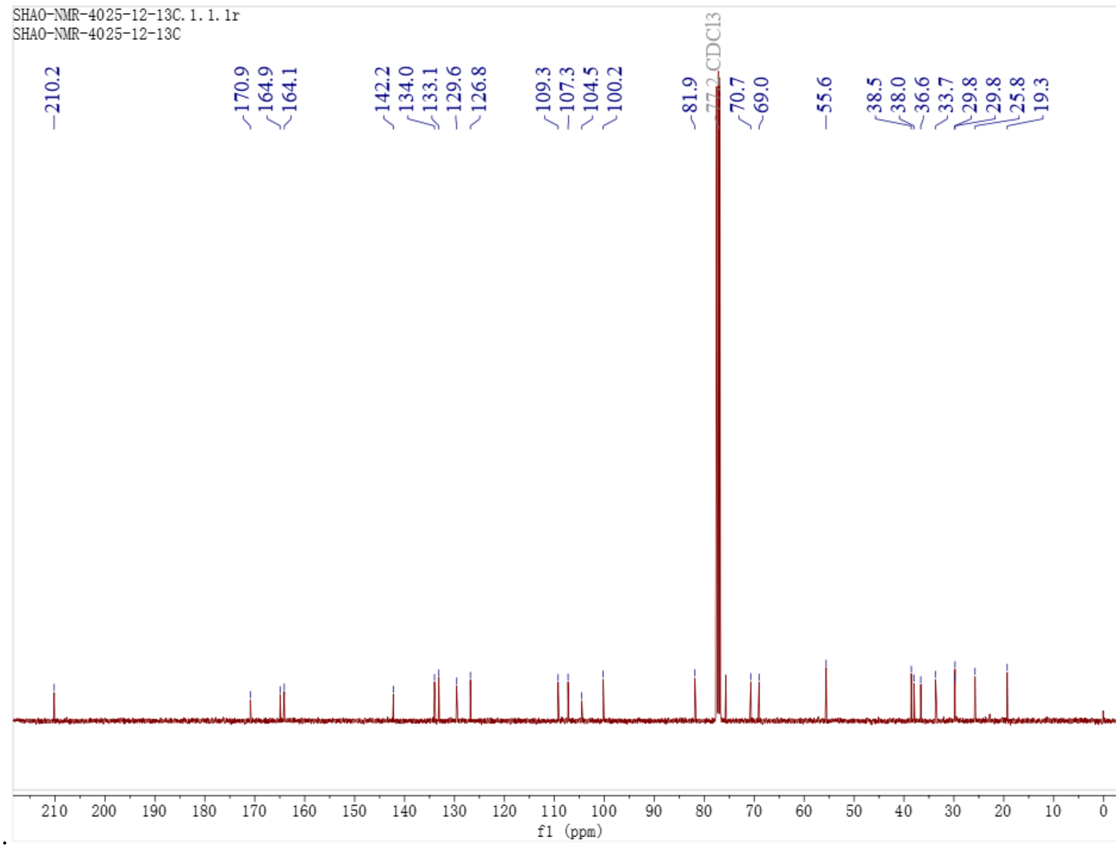

Figure S51. ^{13}C NMR (100 MHz, Chloroform-*d*) spectrum of compound **24.**

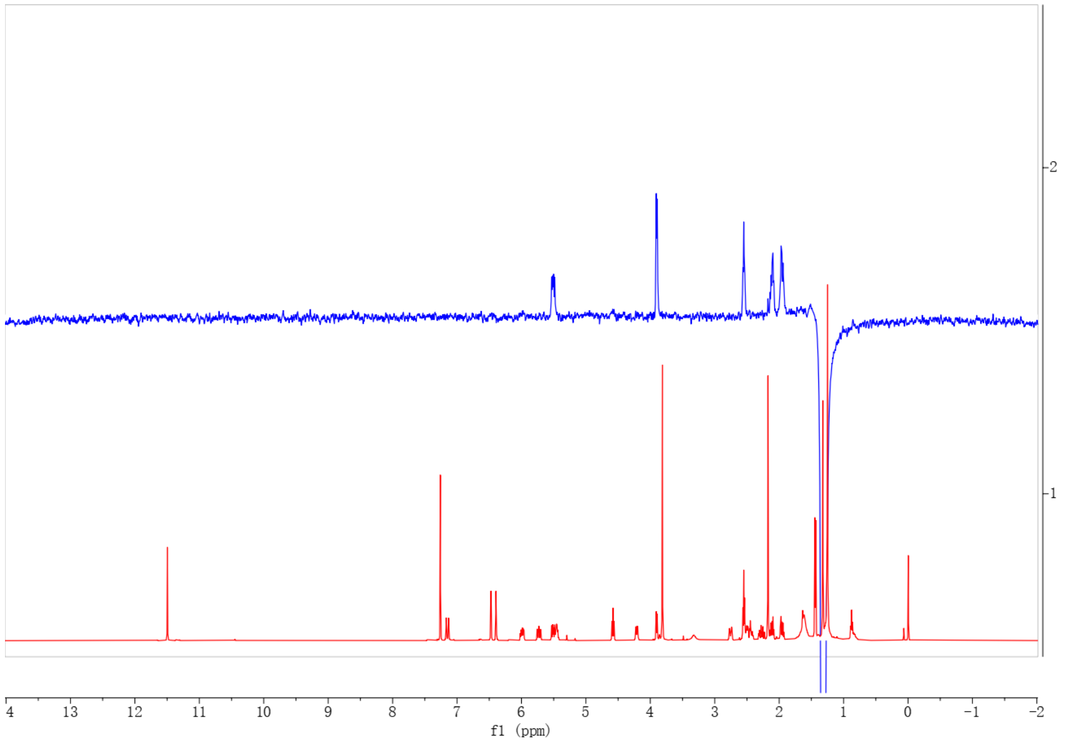

Figure S52. 1D NOE spectrum of compound **24**

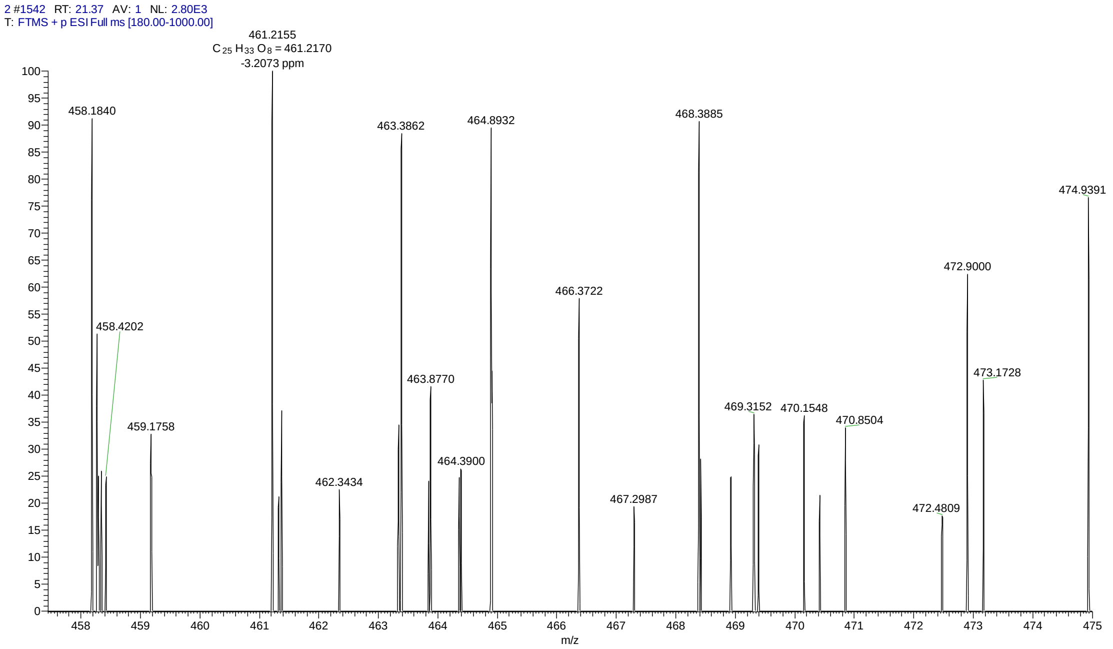

Figure S53. HR-ESI-MS spectrum of compound **24**.

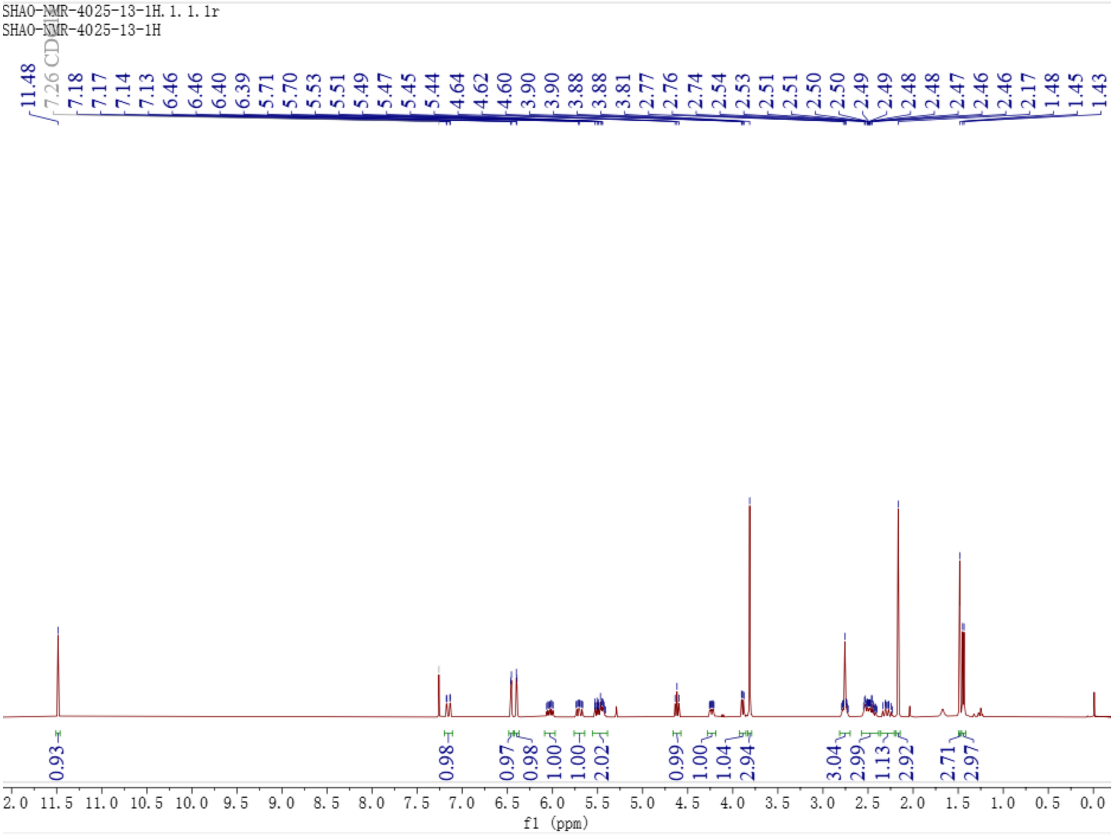

Figure S54. ^{1}H NMR (400 MHz, Chloroform-*d*) spectrum of compound **25.**

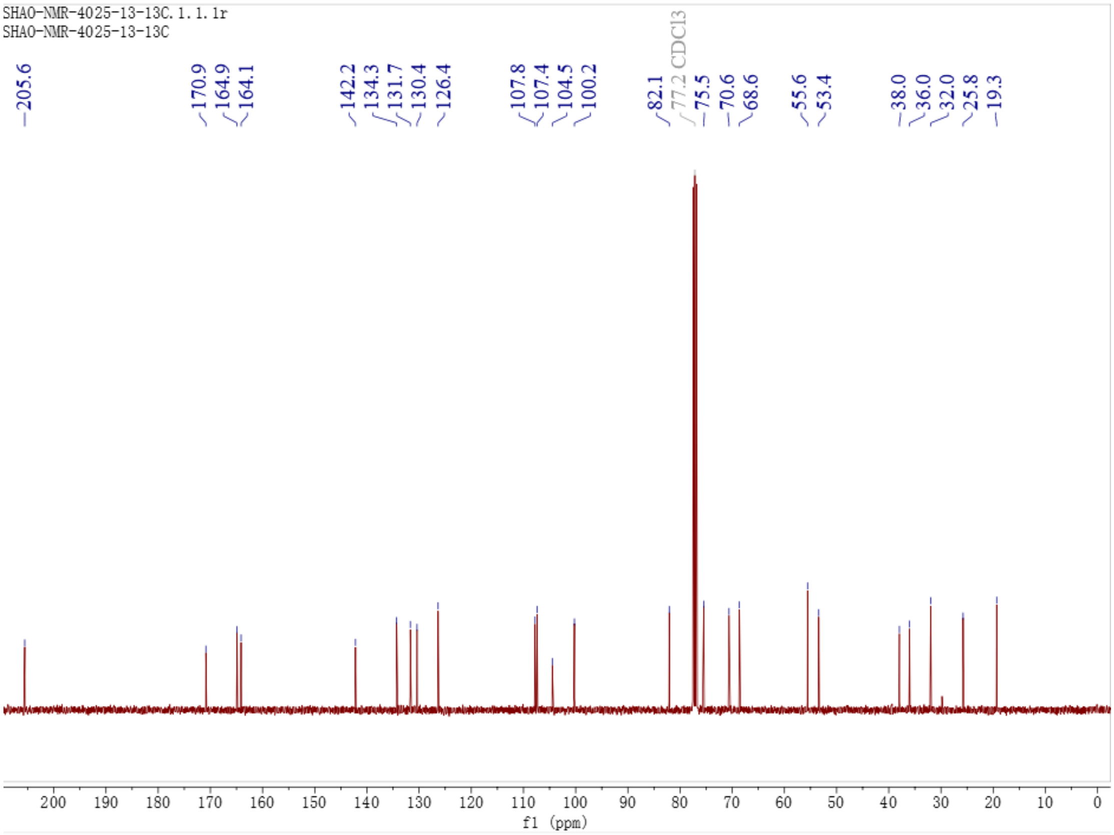

Figure S55. ^{13}C NMR (100 MHz, Chloroform-*d*) spectrum of compound **25**.

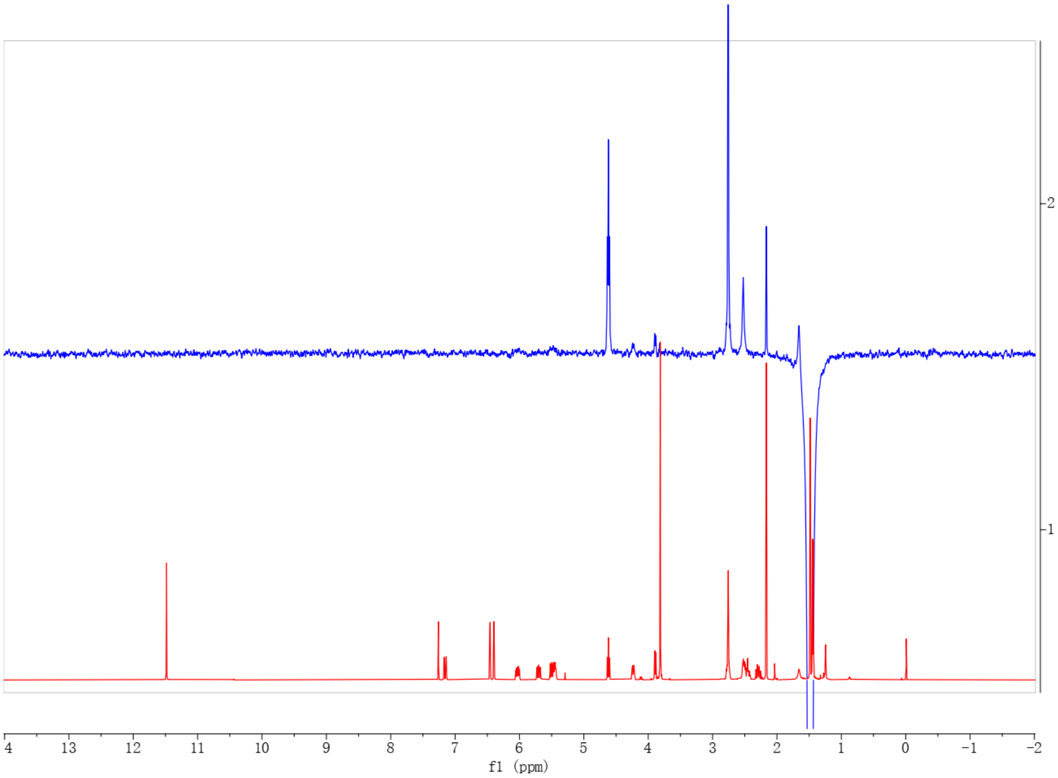

Figure S56. 1D NOE spectrum of compound **25**

\

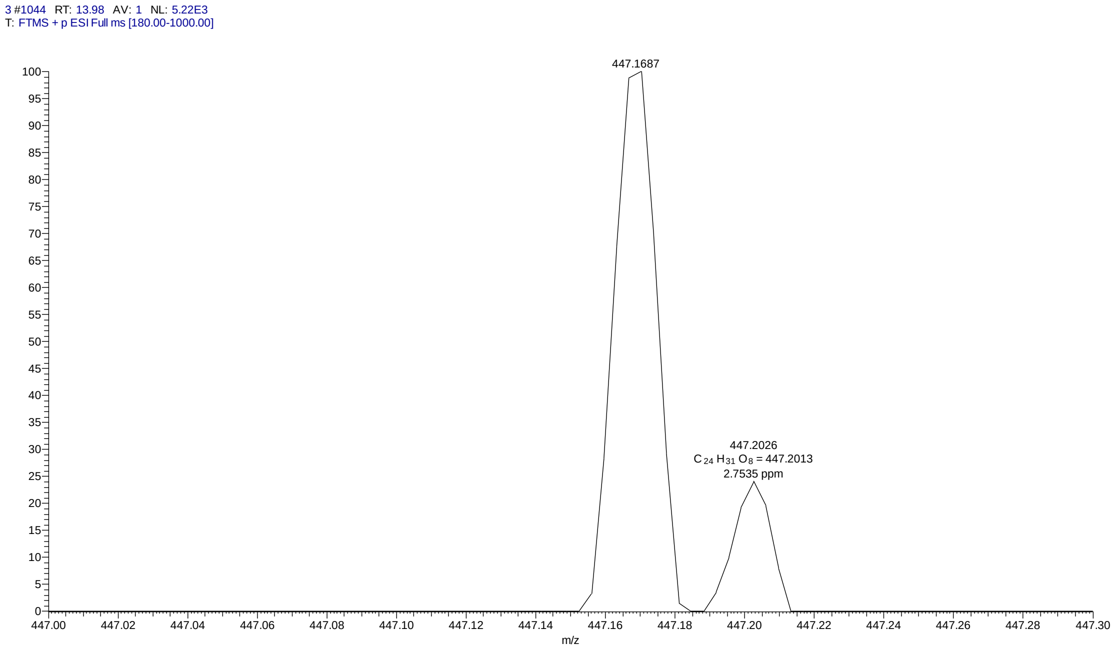

Figure S57. HR-ESI-MS spectrum of compound **25**.

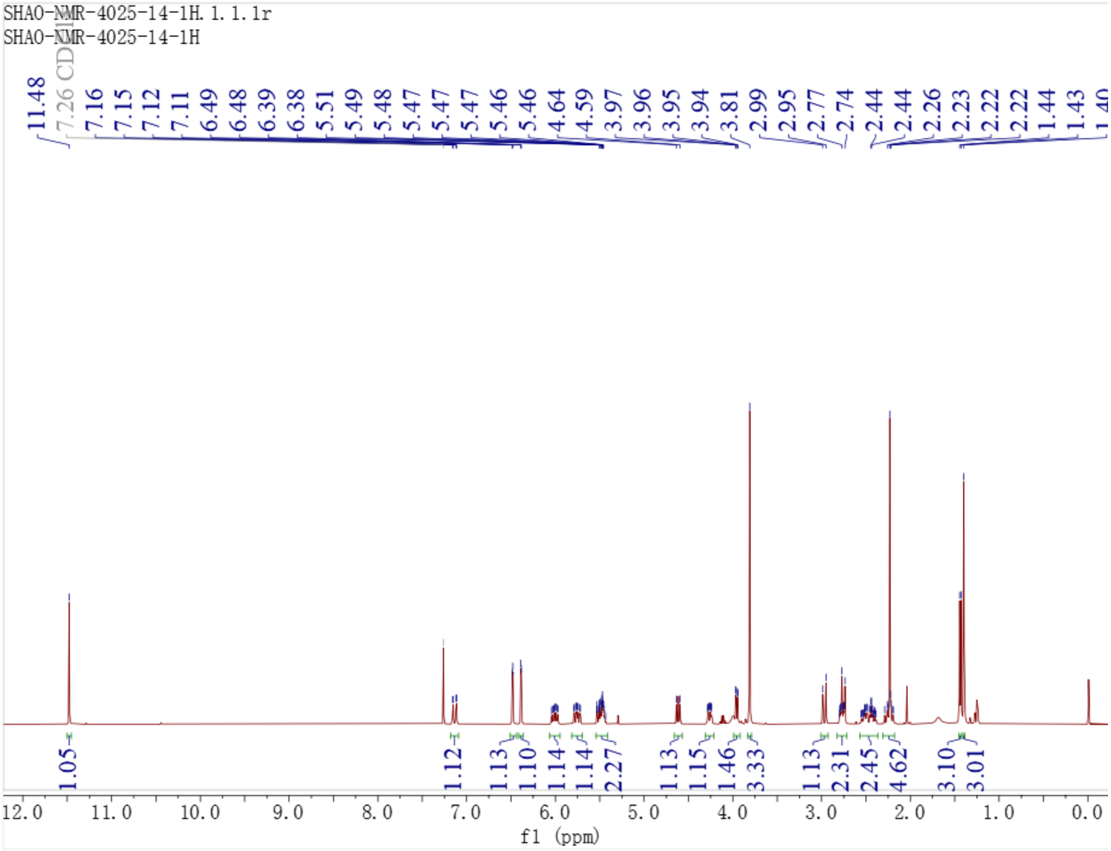

Figure S58. ^{1}H NMR (400 MHz, Chloroform-*d*) spectrum of compound **26.**

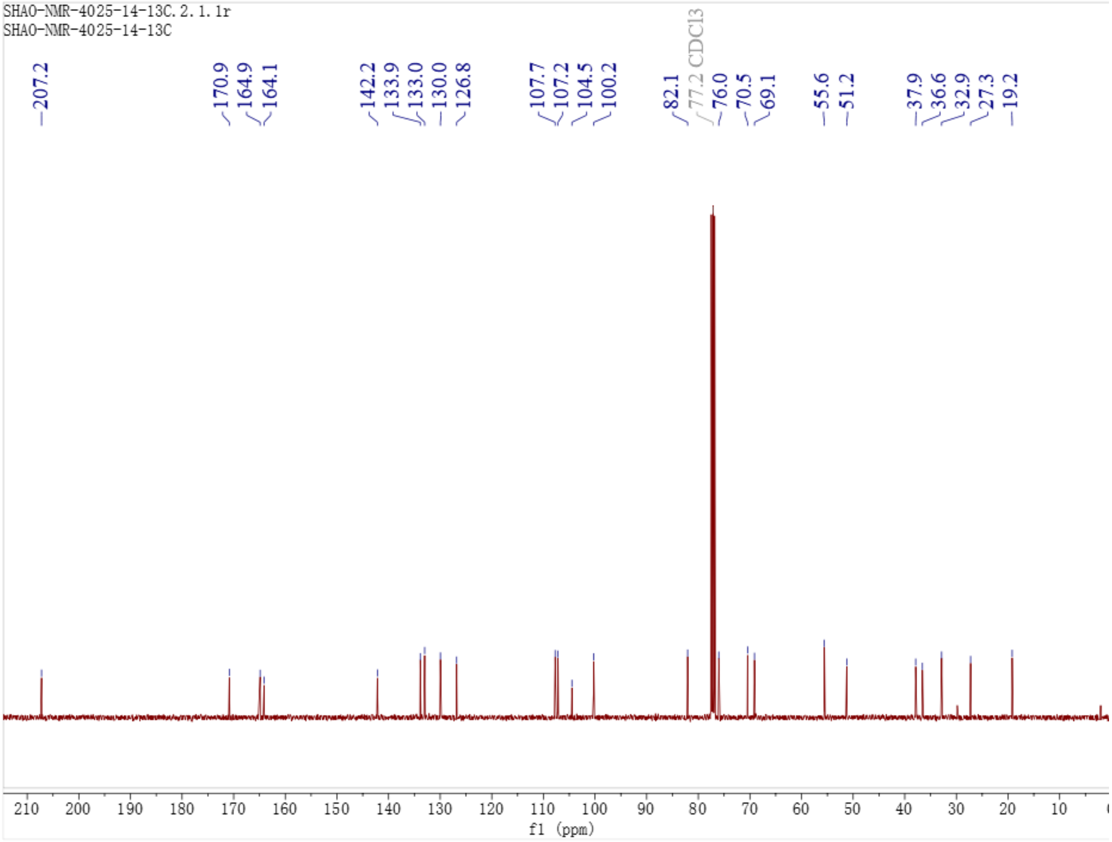

Figure S59. ^{13}C NMR (100 MHz, Chloroform-*d*) spectrum of compound **26.**

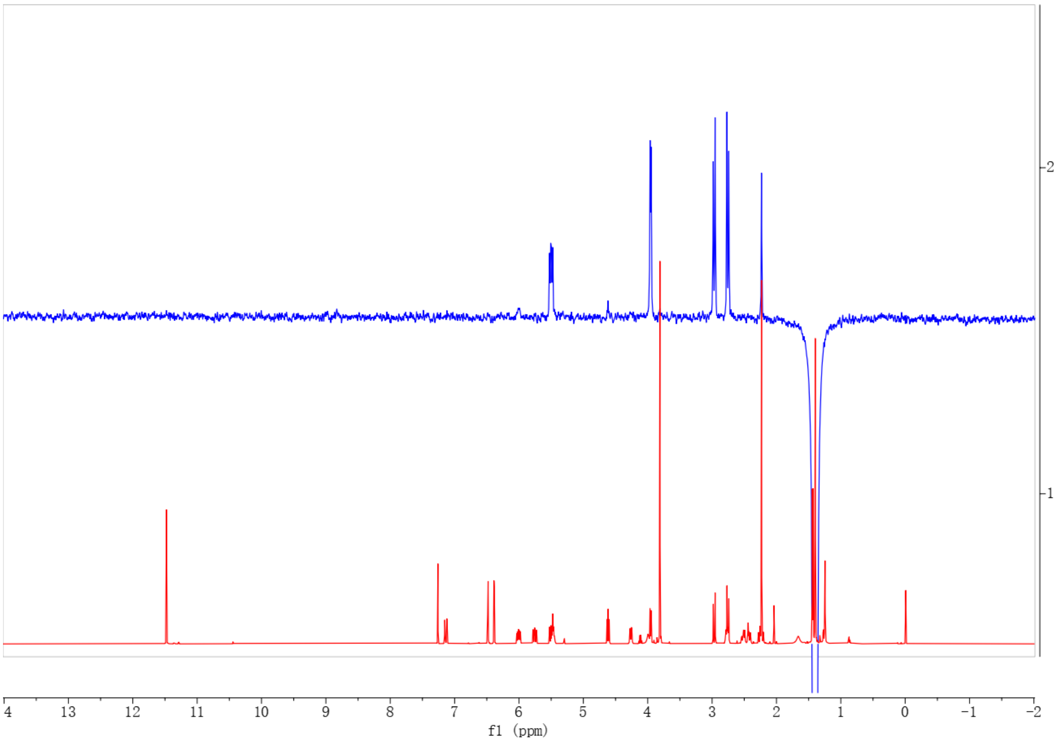

Figure S60. 1D NOE spectrum of compound **26**

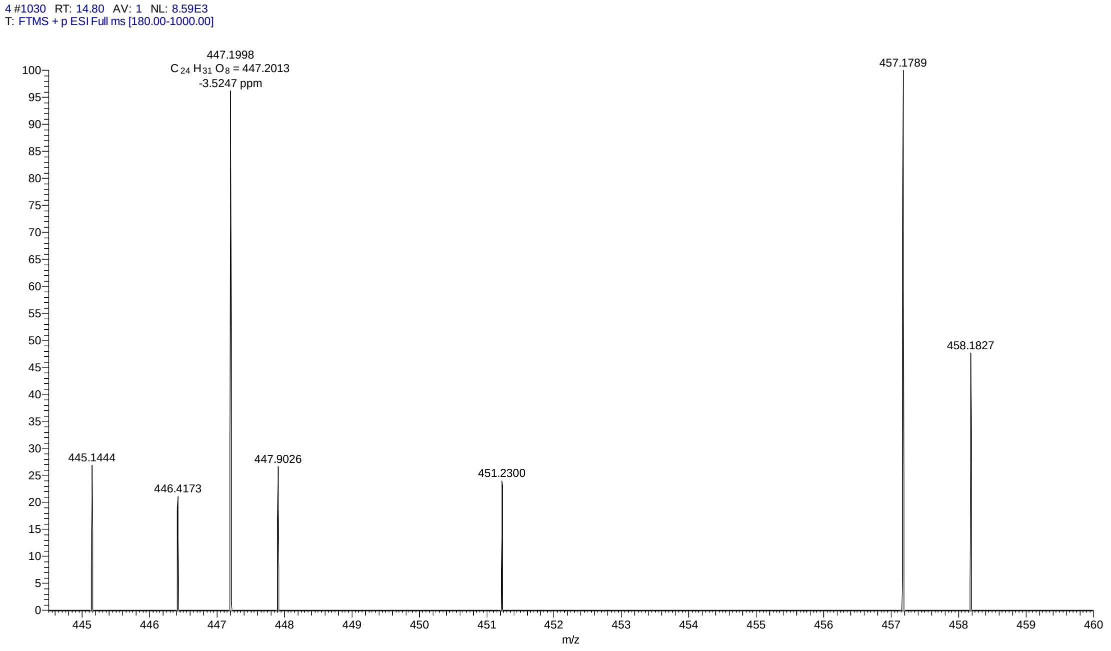

Figure S61. HR-ESI-MS spectrum of compound **26**.

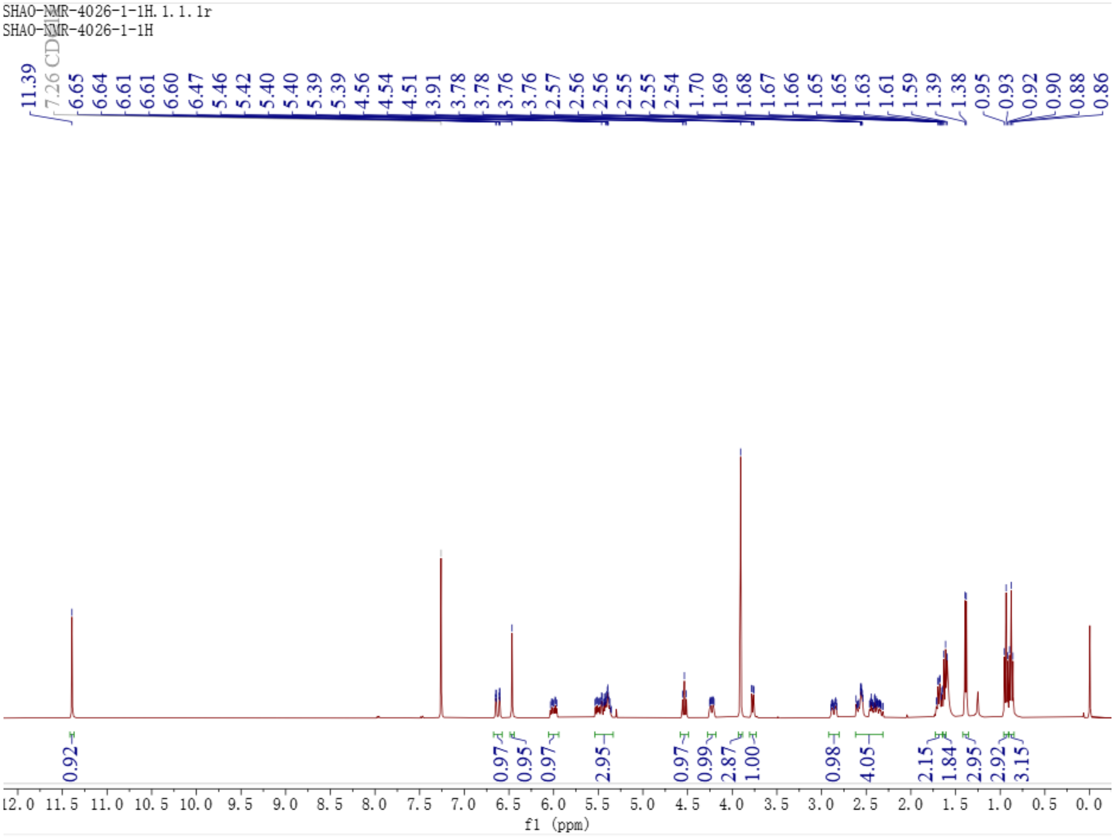

Figure S62. ^{1}H NMR (400 MHz, Chloroform-*d*) spectrum of compound **27.**

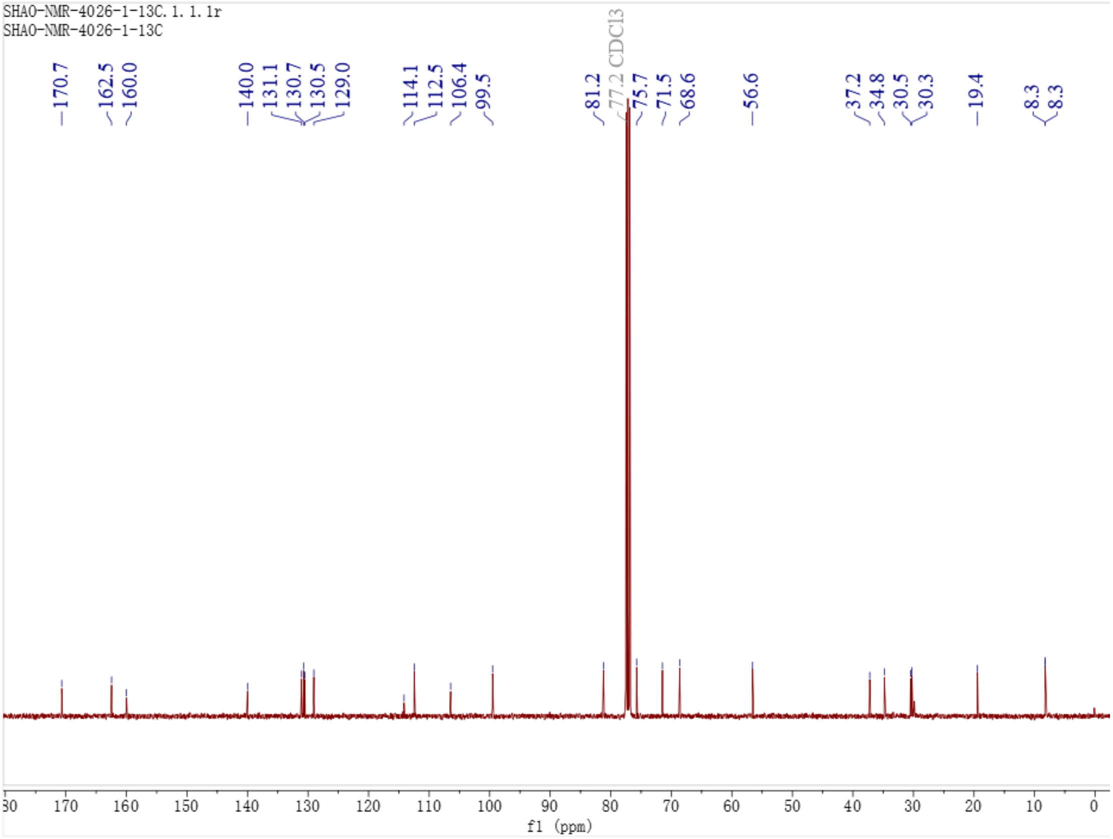

Figure S63. ^{13}C NMR (100 MHz, Chloroform-*d*) spectrum of compound **27.**

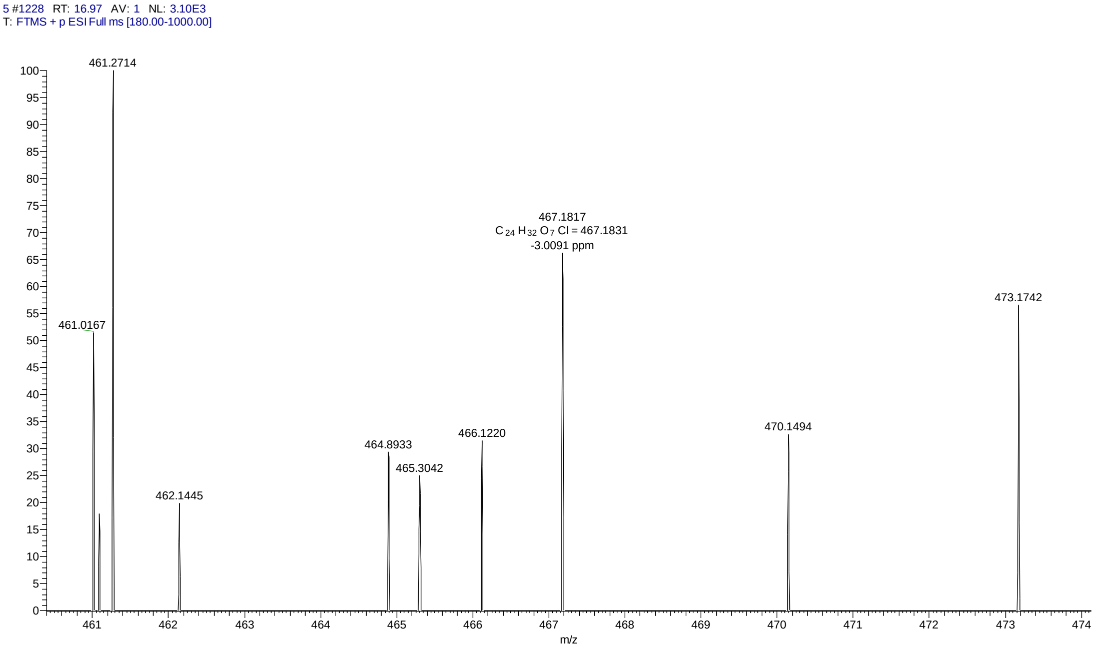

Figure S64. HR-ESI-MS spectrum of compound **27**.

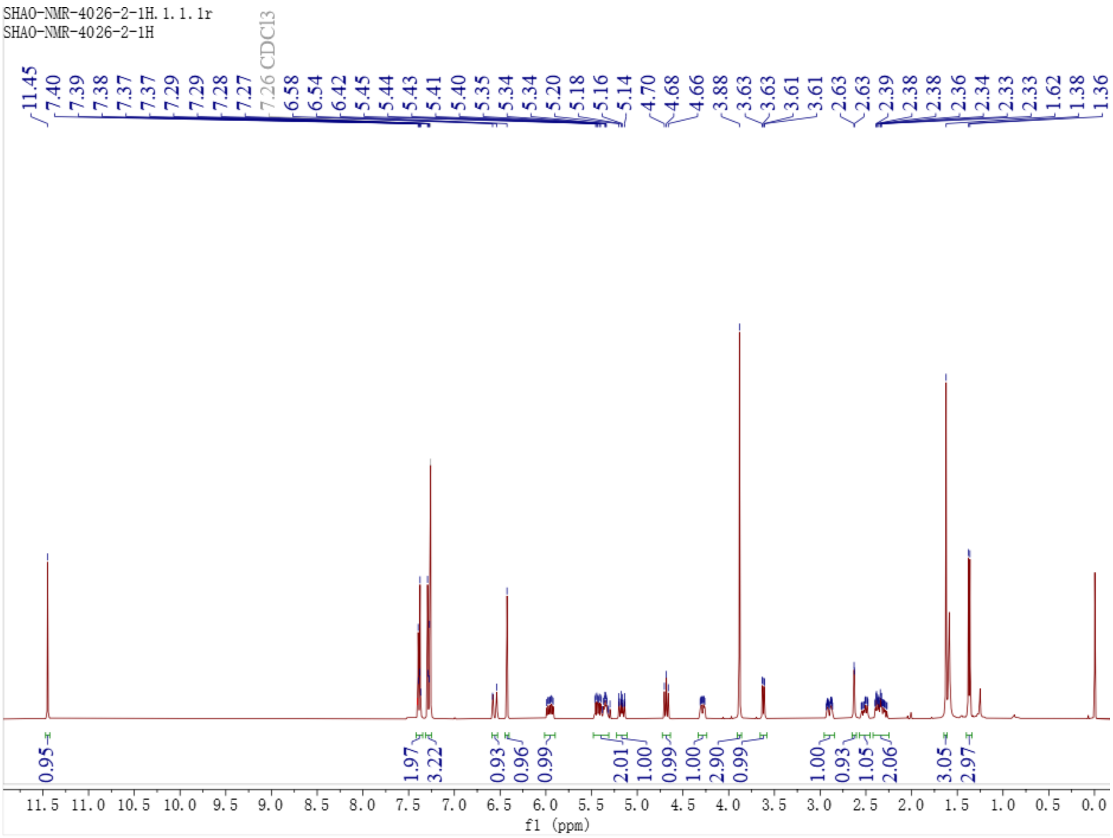

Figure S65. ^{1}H NMR (400 MHz, Chloroform-*d*) spectrum of compound **28.**

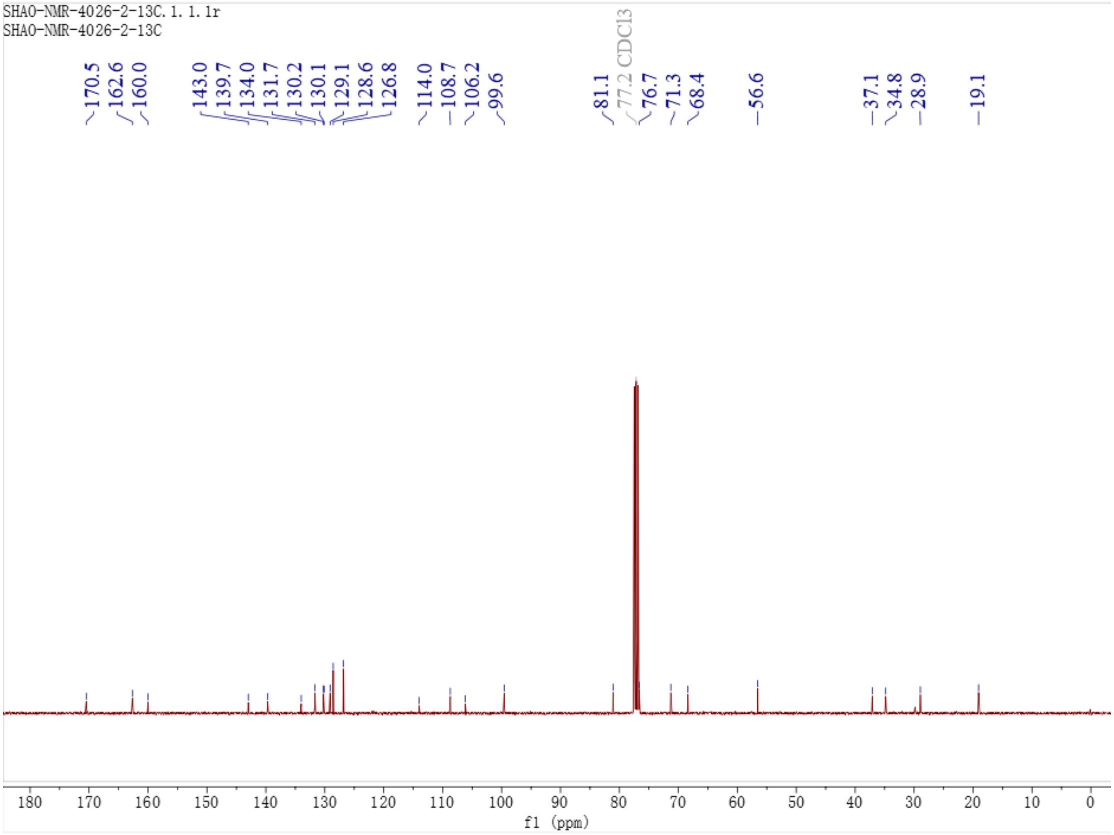

Figure S66. ^{13}C NMR (100 MHz, Chloroform-*d*) spectrum of compound 28.

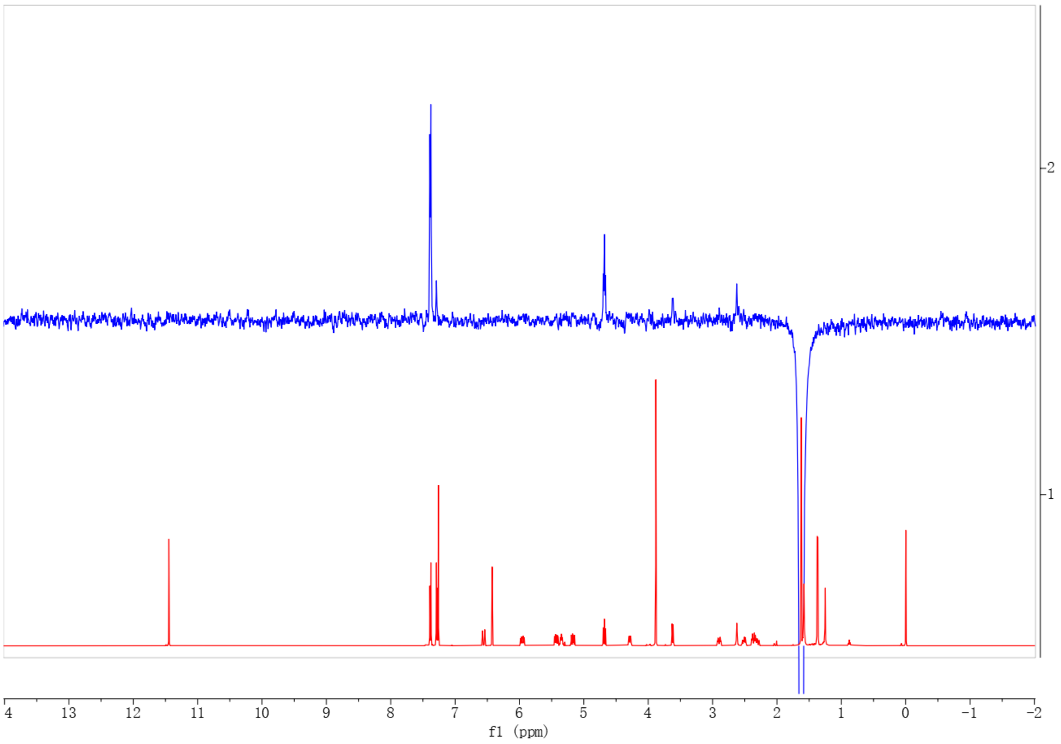

Figure S67. 1D NOE spectrum of compound **28**

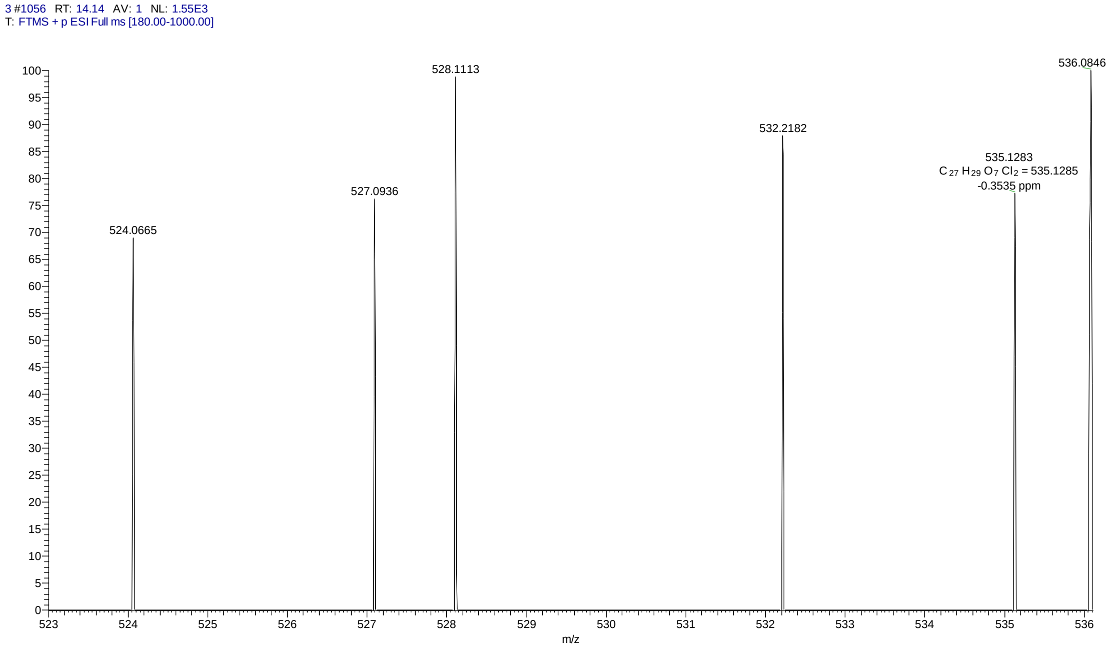

Figure S68. HR-ESI-MS spectrum of compound **28**.

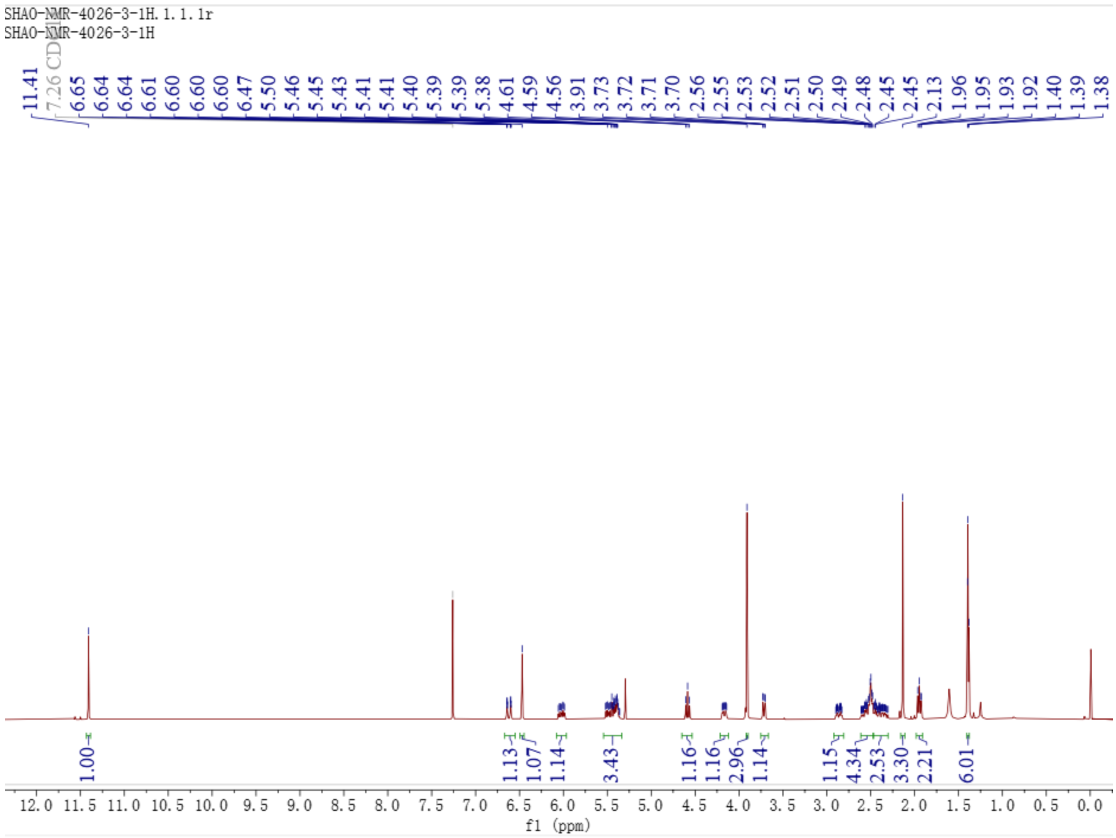

Figure S69. ^{1}H NMR (400 MHz, Chloroform-*d*) spectrum of compound **29**.

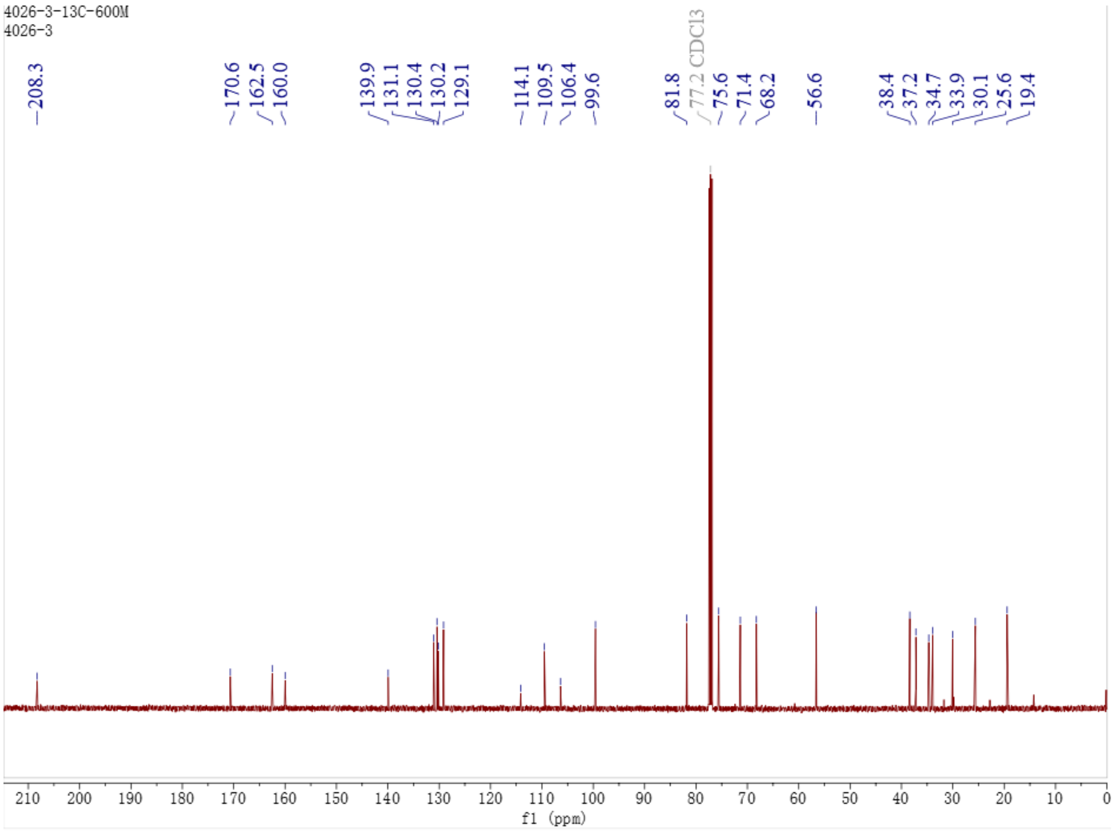

Figure S70. ^{13}C NMR (125 MHz, Chloroform-*d*) spectrum of compound **29**.

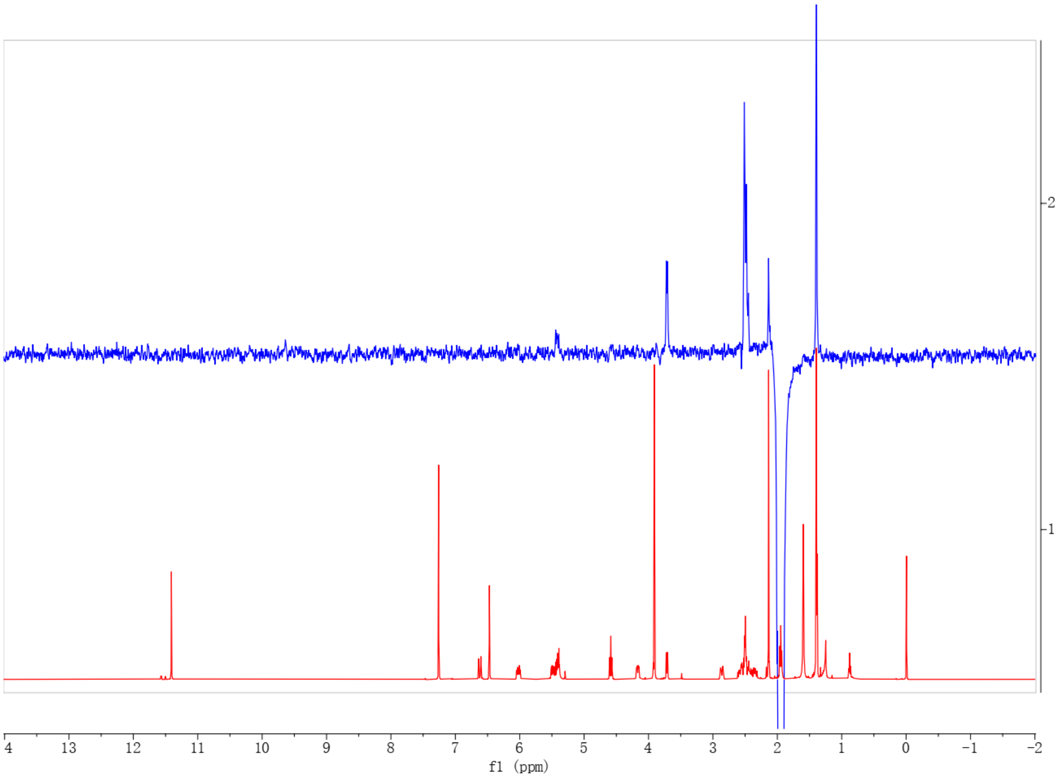

Figure S71. 1D NOE spectrum of compound **29.**

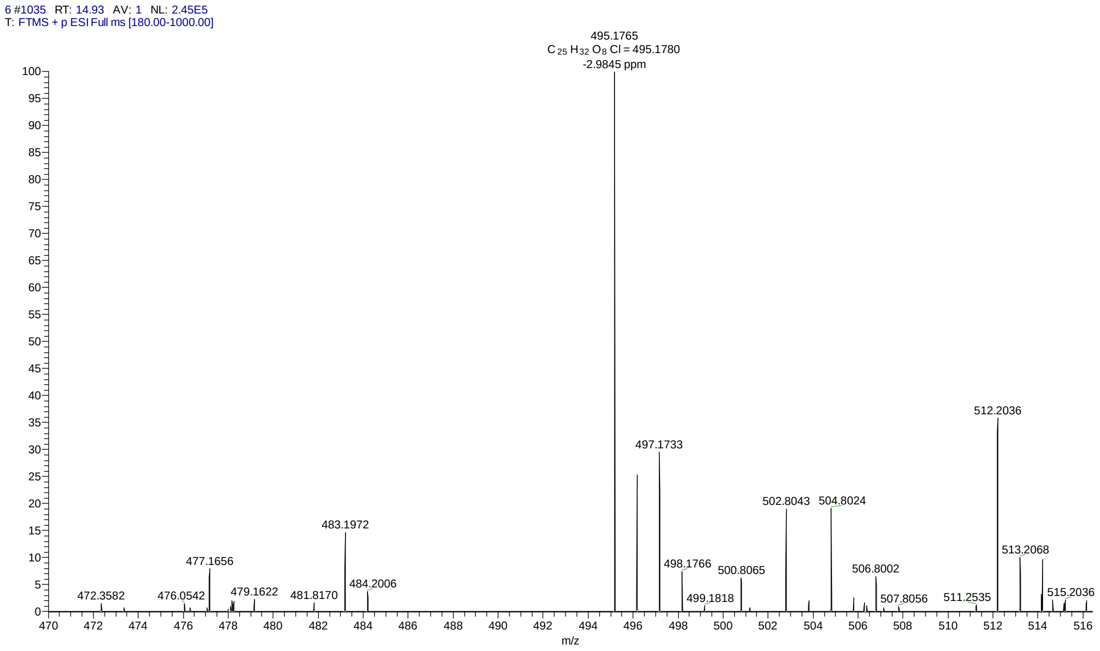

Figure S72. HR-ESI-MS spectrum of compound **29**.

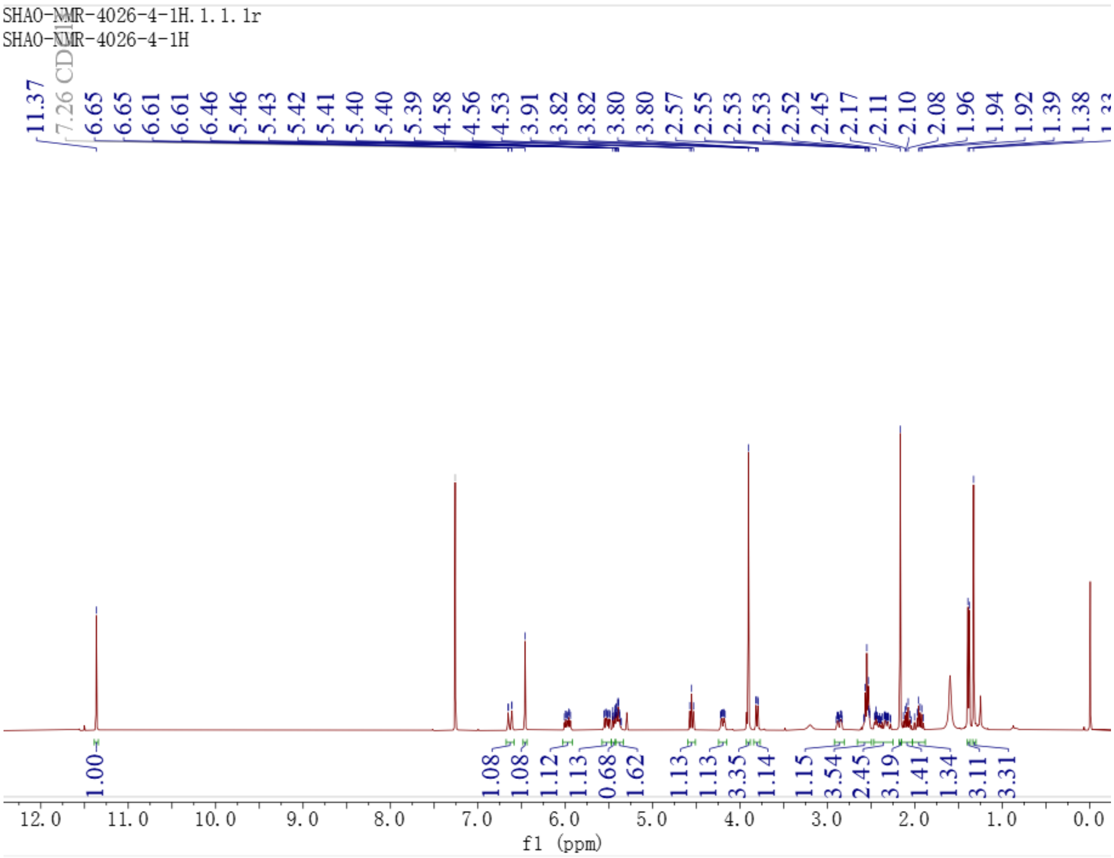

Figure S73. ^{1}H NMR (400 MHz, Chloroform-*d*) spectrum of compound **30.**

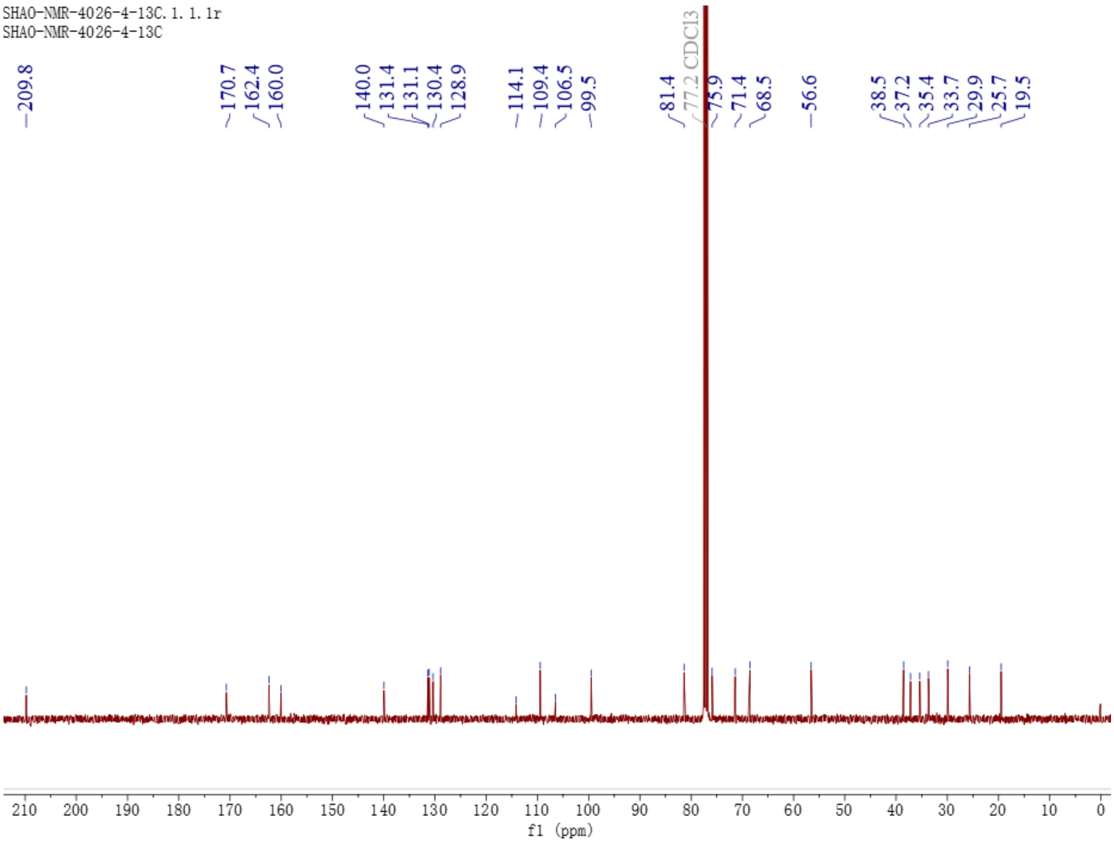

Figure S74. ^{13}C NMR (100 MHz, Chloroform-*d*) spectrum of compound **30.**

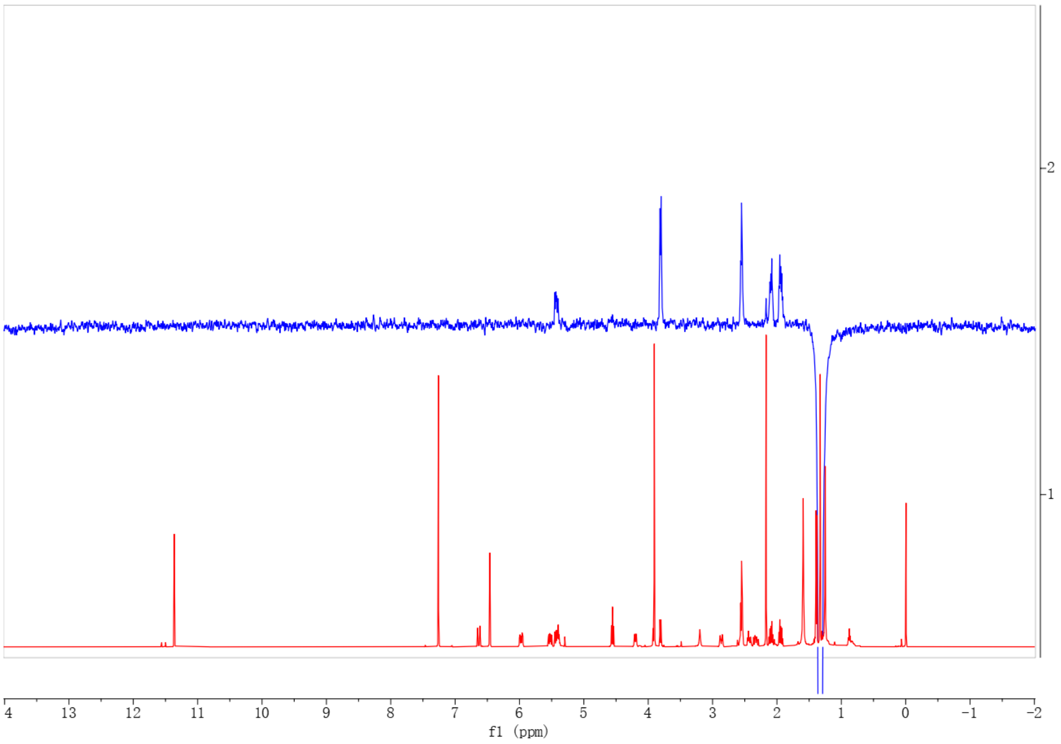

Figure S75. 1D NOE spectrum of compound **30**

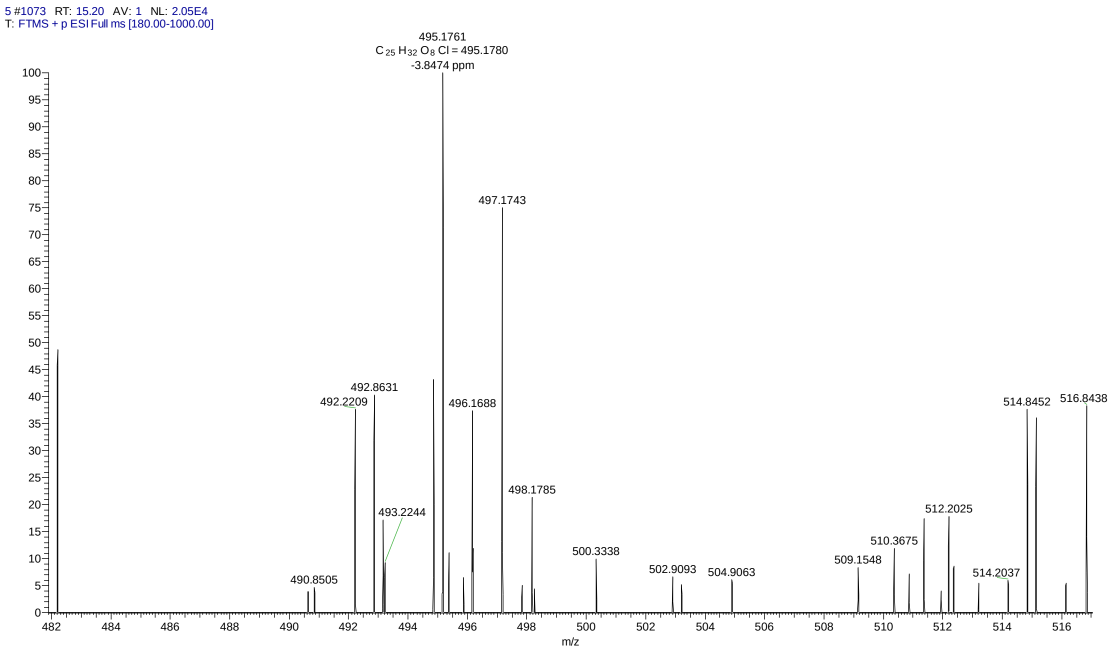

Figure S76. HR-ESI-MS spectrum of compound **30**.

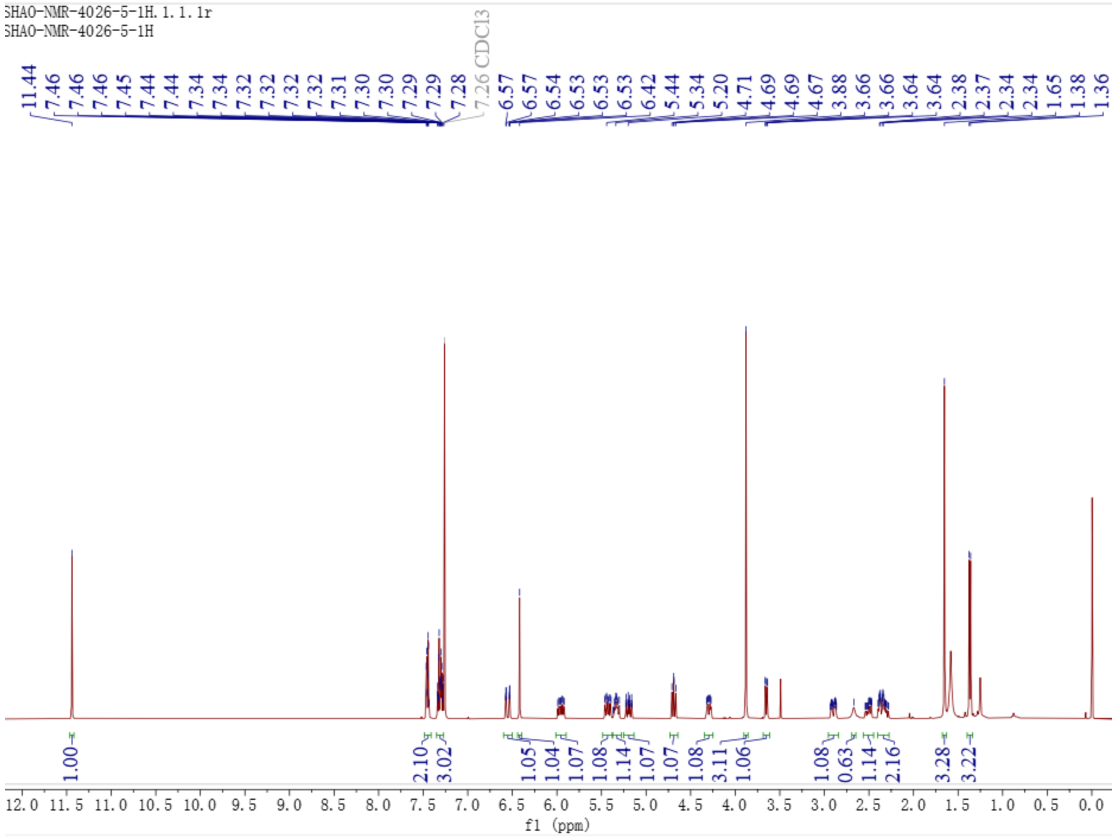

Figure S77. ^{1}H NMR (400 MHz, Chloroform-*d*) spectrum of compound **31.**

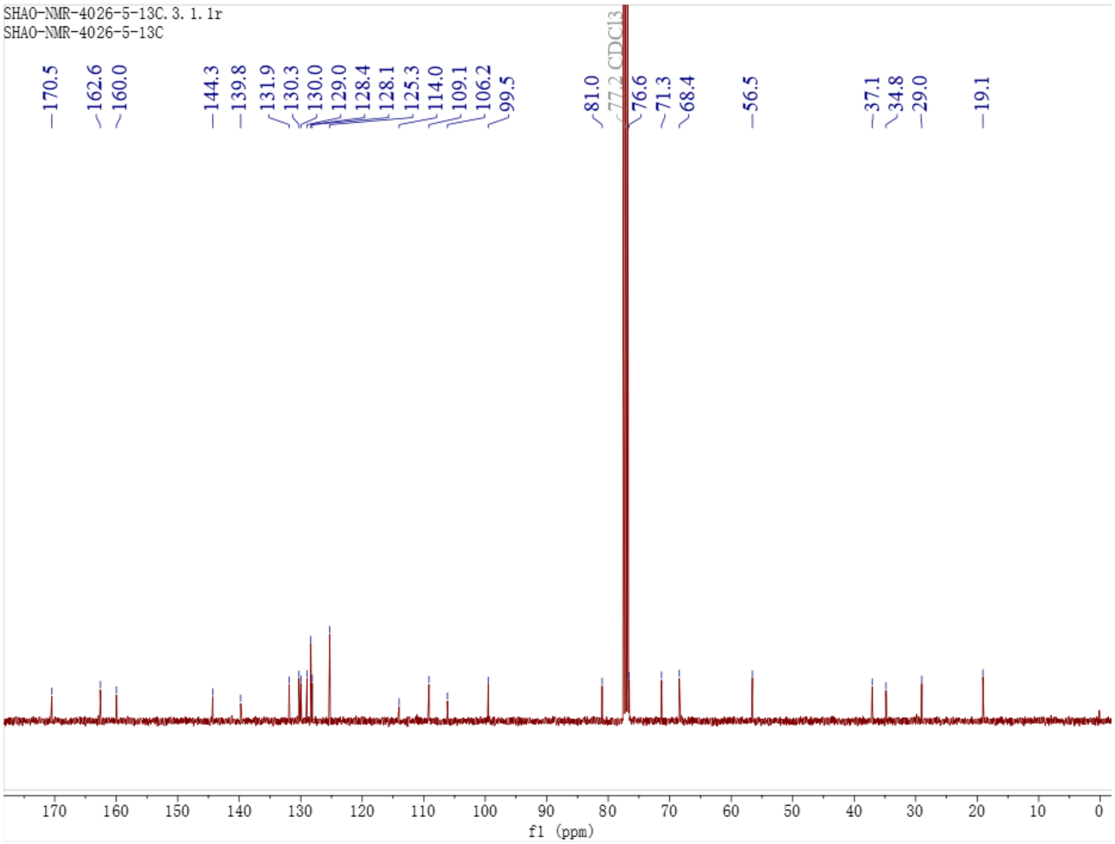

Figure S78. ^{13}C NMR (100 MHz, Chloroform-*d*) spectrum of compound **31**.

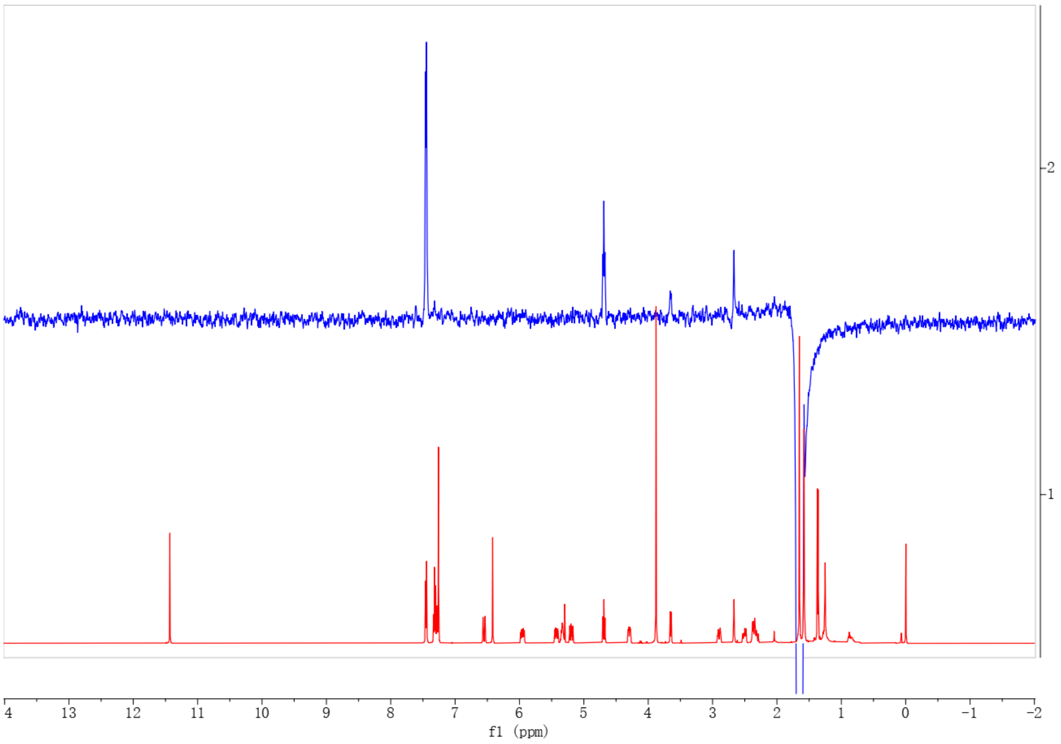

Figure S79. 1D NOE spectrum of compound **31**

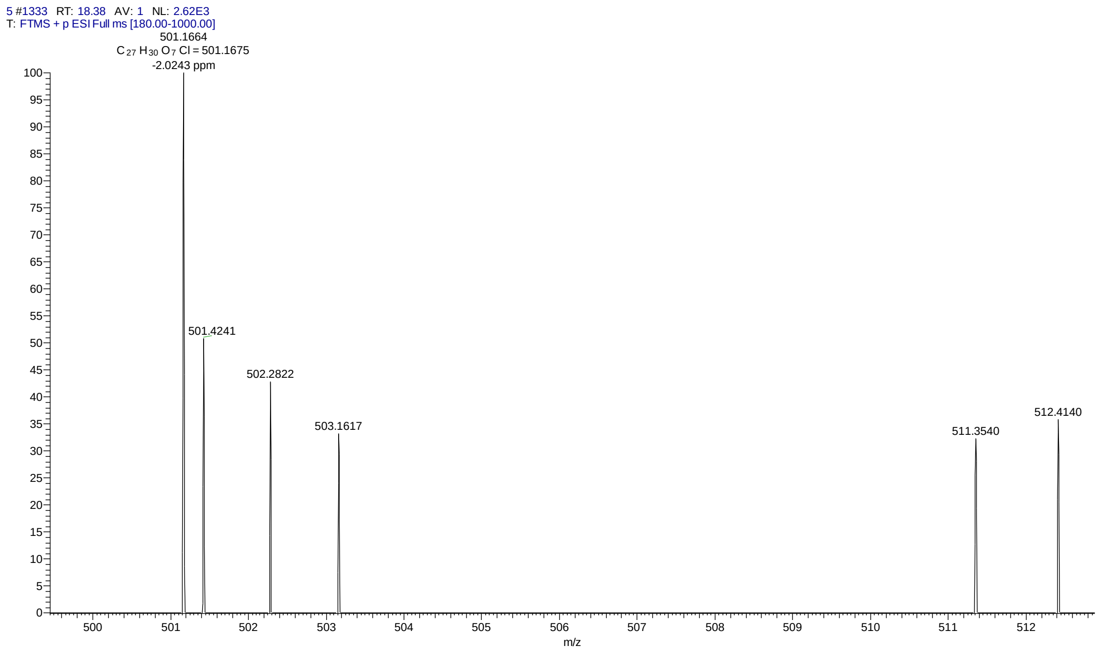

Figure S80. HR-ESI-MS spectrum of compound **31**.

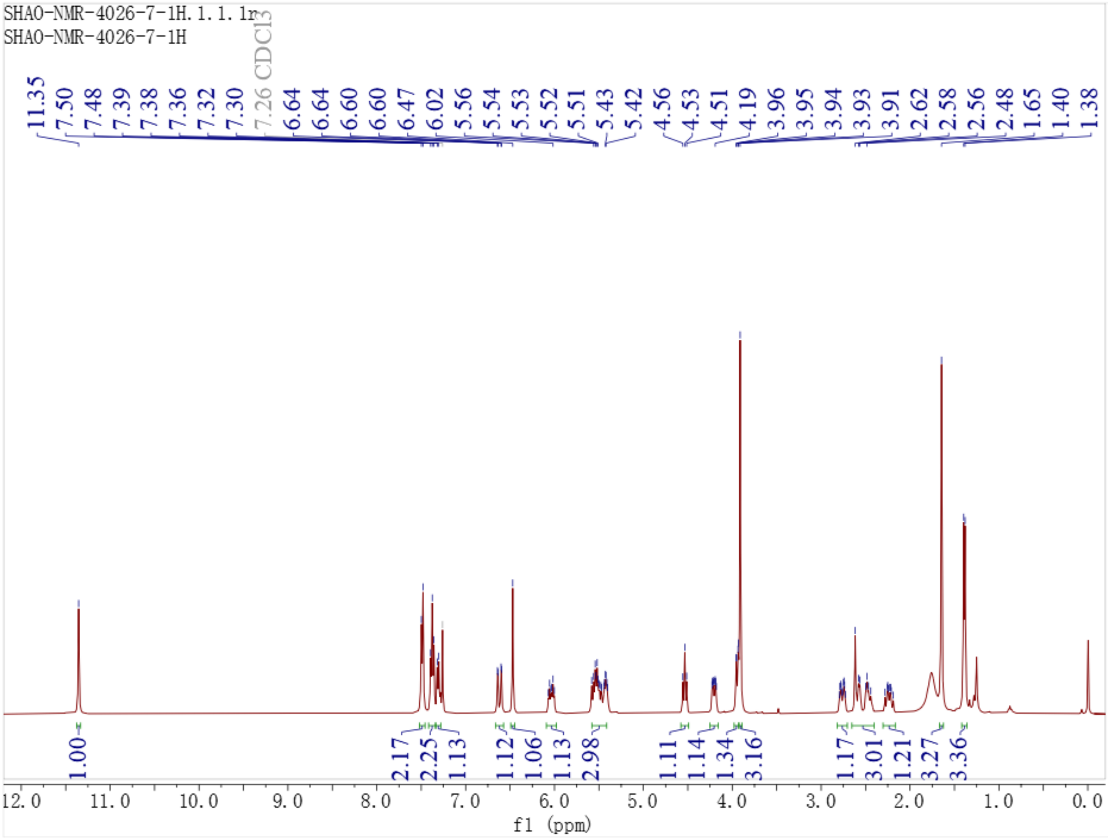

Figure S81. ^{1}H NMR (400 MHz, Chloroform-*d*) spectrum of compound **32.**

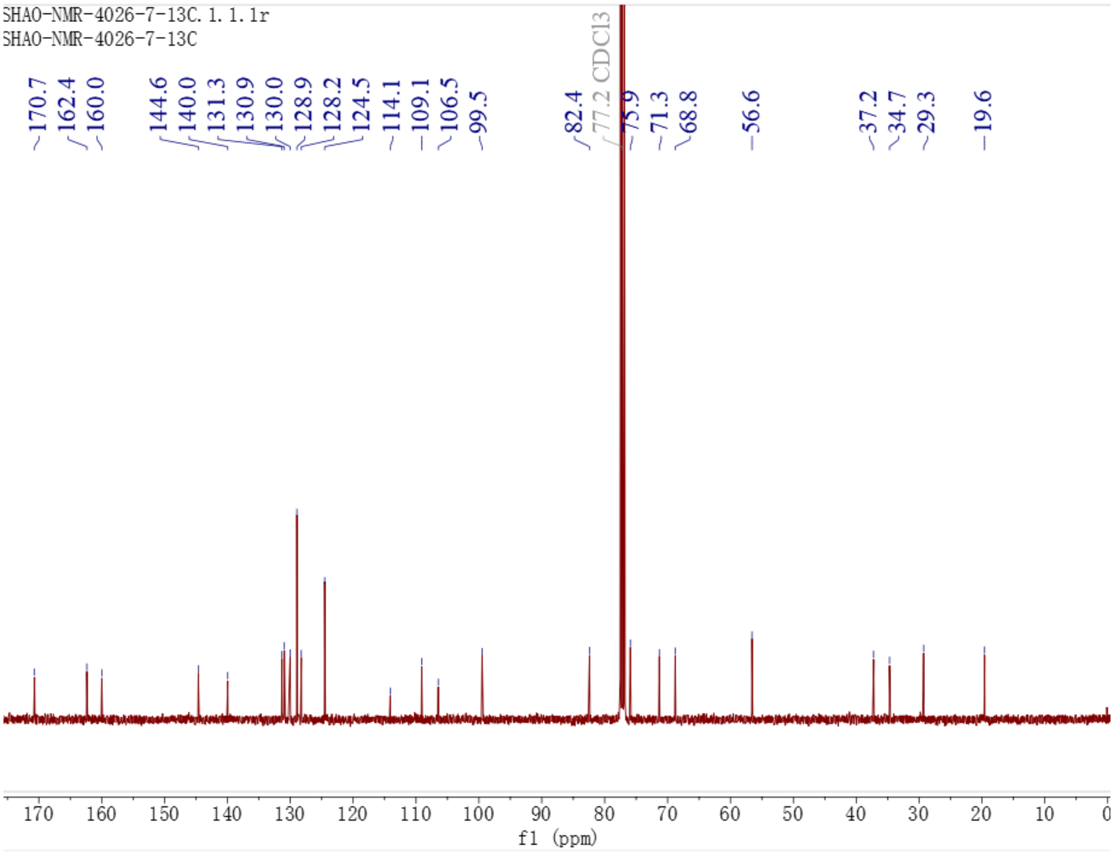

Figure S82. ^{13}C NMR (100 MHz, Chloroform-*d*) spectrum of compound **32.**

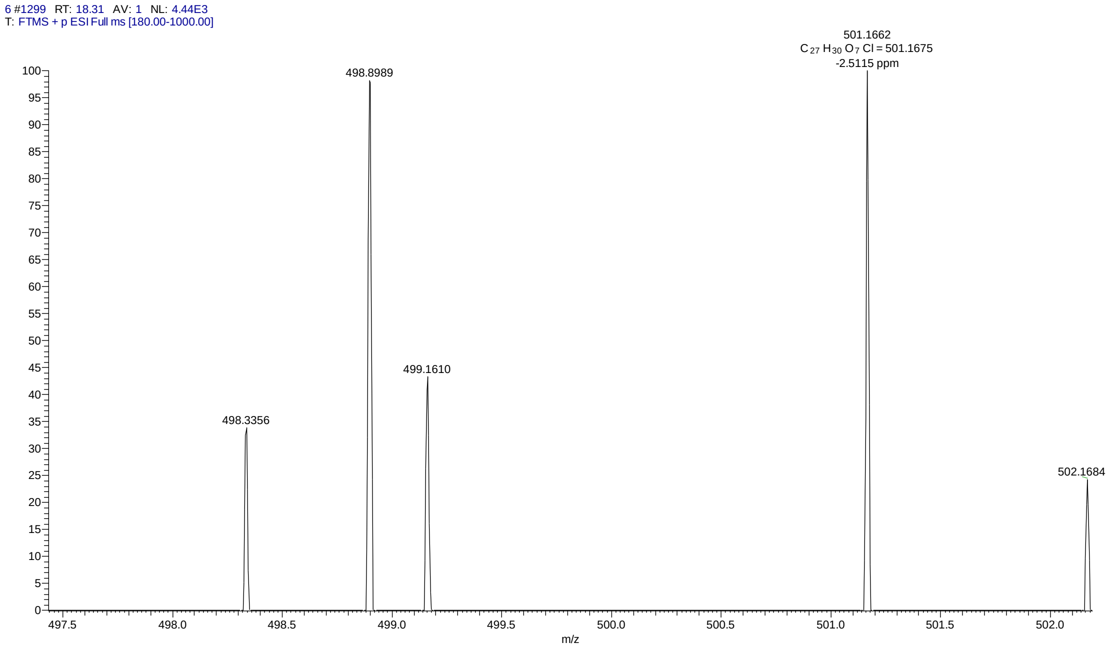

Figure S83. HR-ESI-MS spectrum of compound **32**.

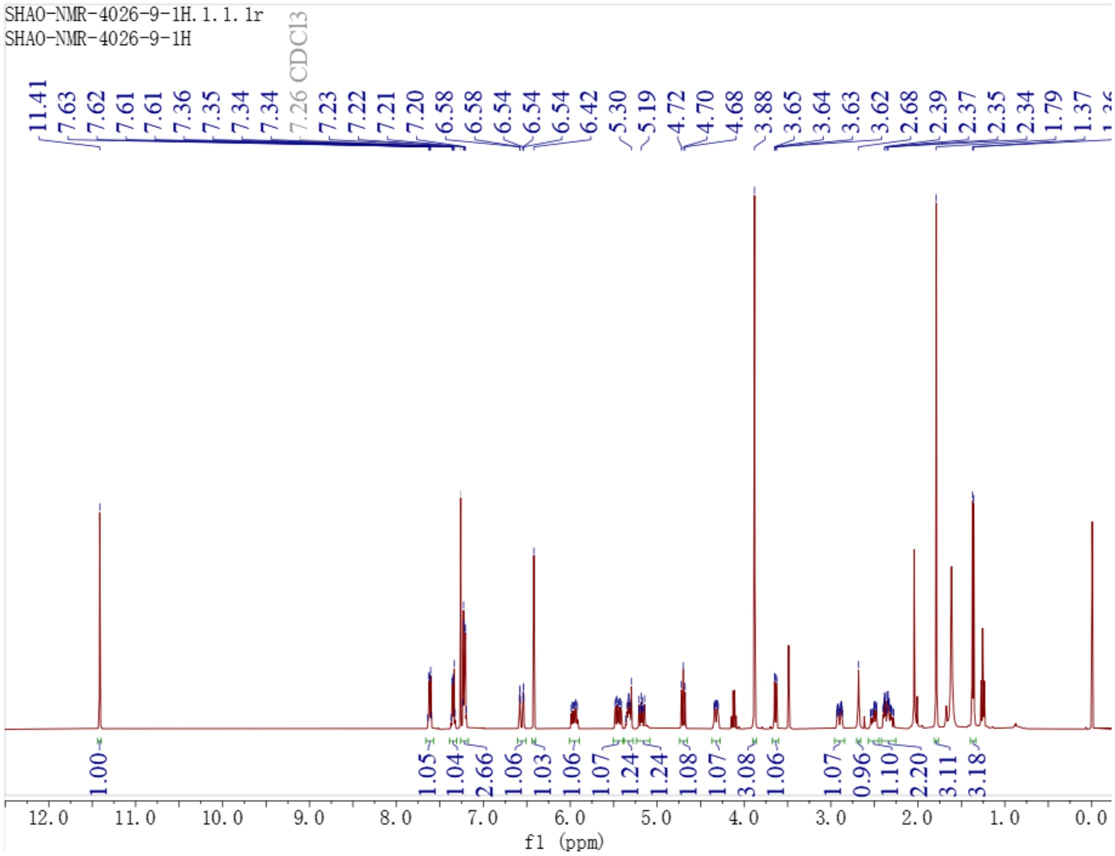

Figure S84. ^{1}H NMR (400 MHz, Chloroform-*d*) spectrum of compound **33.**

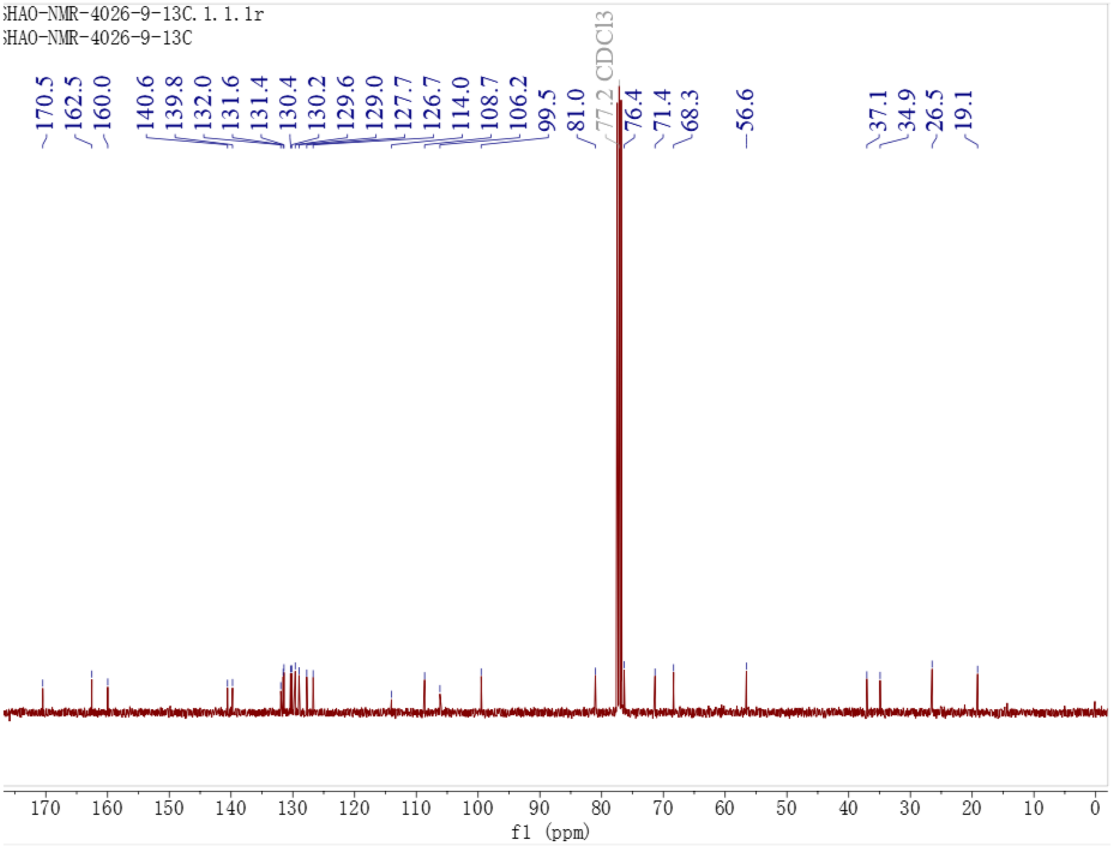

Figure S85. ^{13}C NMR (100 MHz, Chloroform-*d*) spectrum of compound **33**.

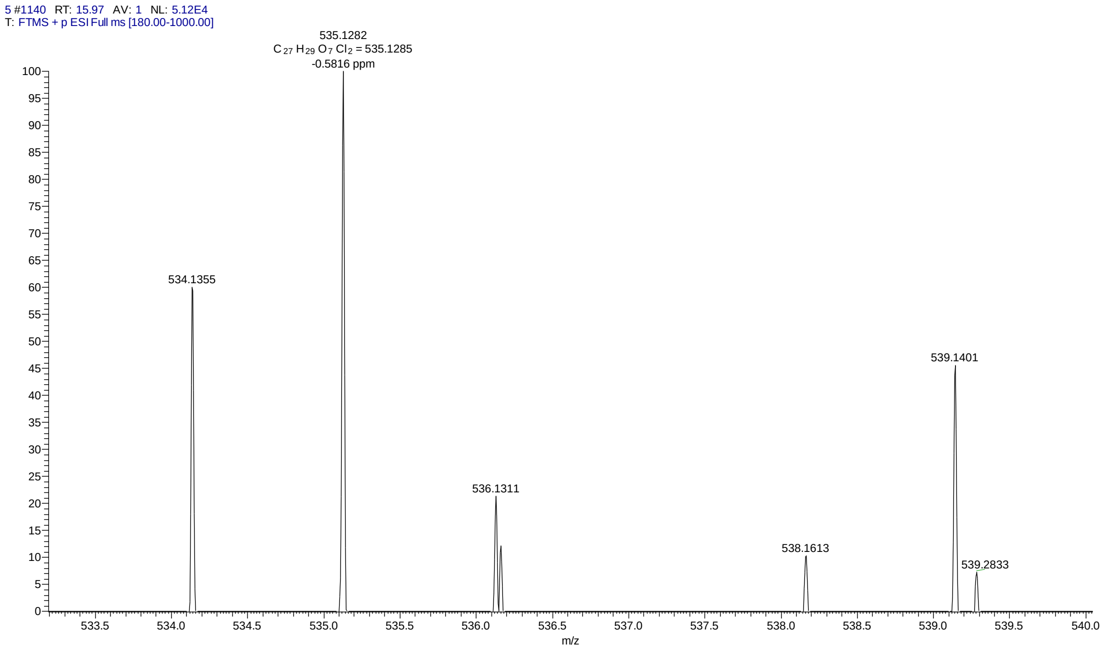

Figure S86. HR-ESI-MS spectrum of compound **33**.

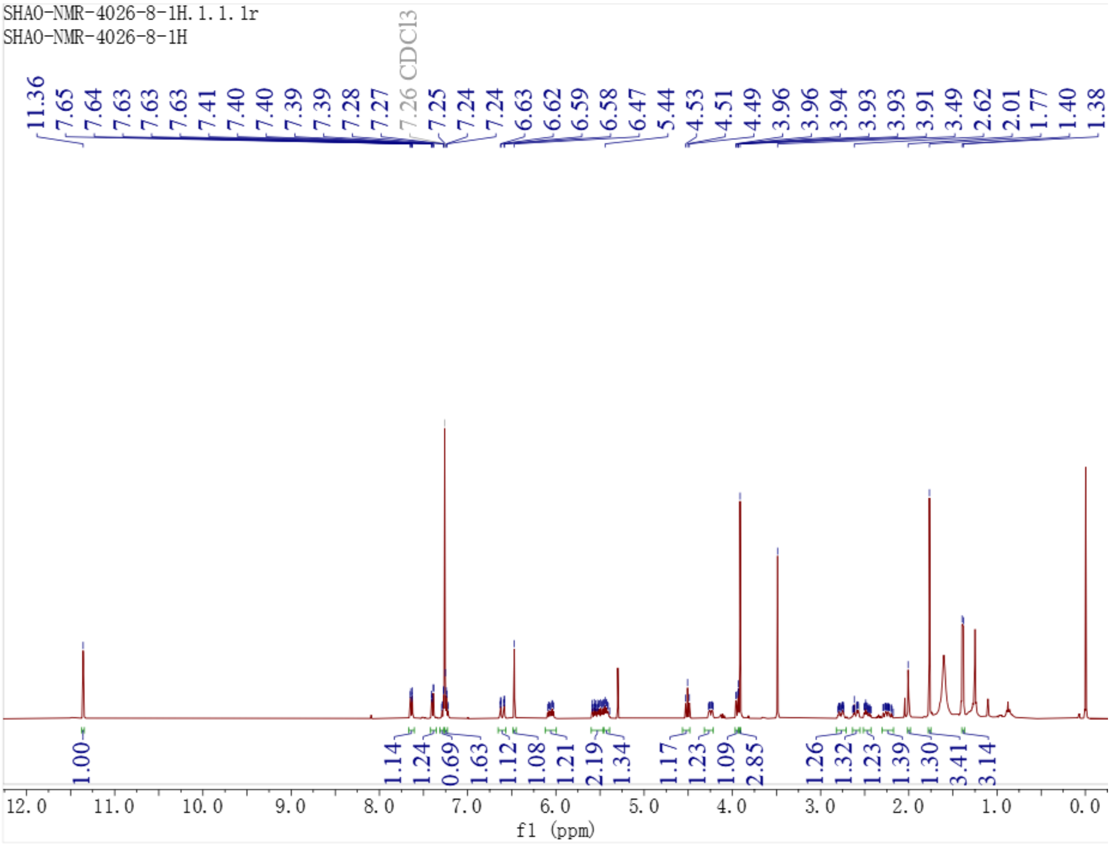

Figure S87. ^{1}H NMR (400 MHz, Chloroform-*d*) spectrum of compound **34.**

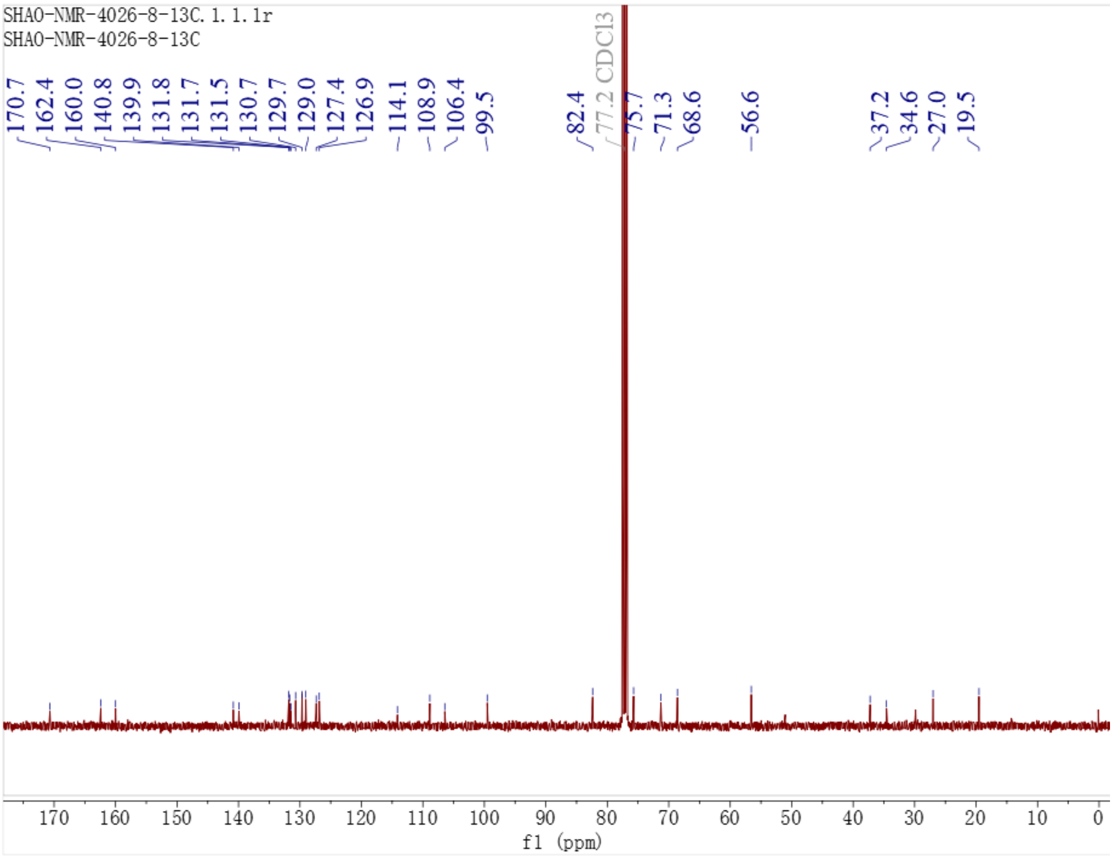

Figure S88. ^{13}C NMR (100 MHz, Chloroform-*d*) spectrum of compound **34**.

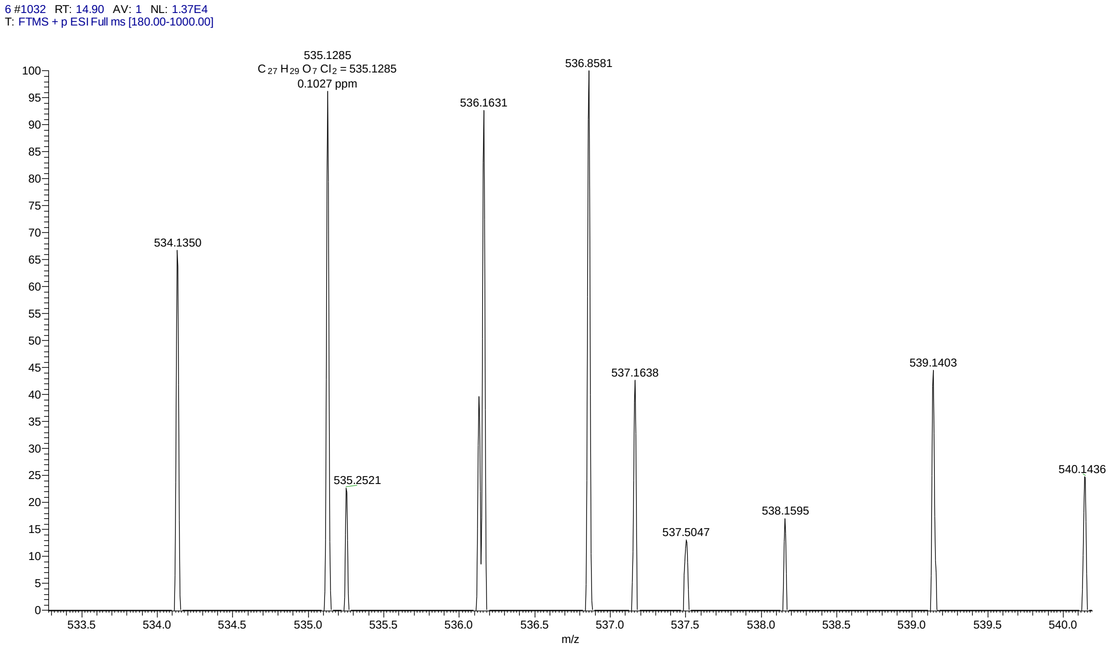

Figure S89. HR-ESI-MS spectrum of compound **34**.

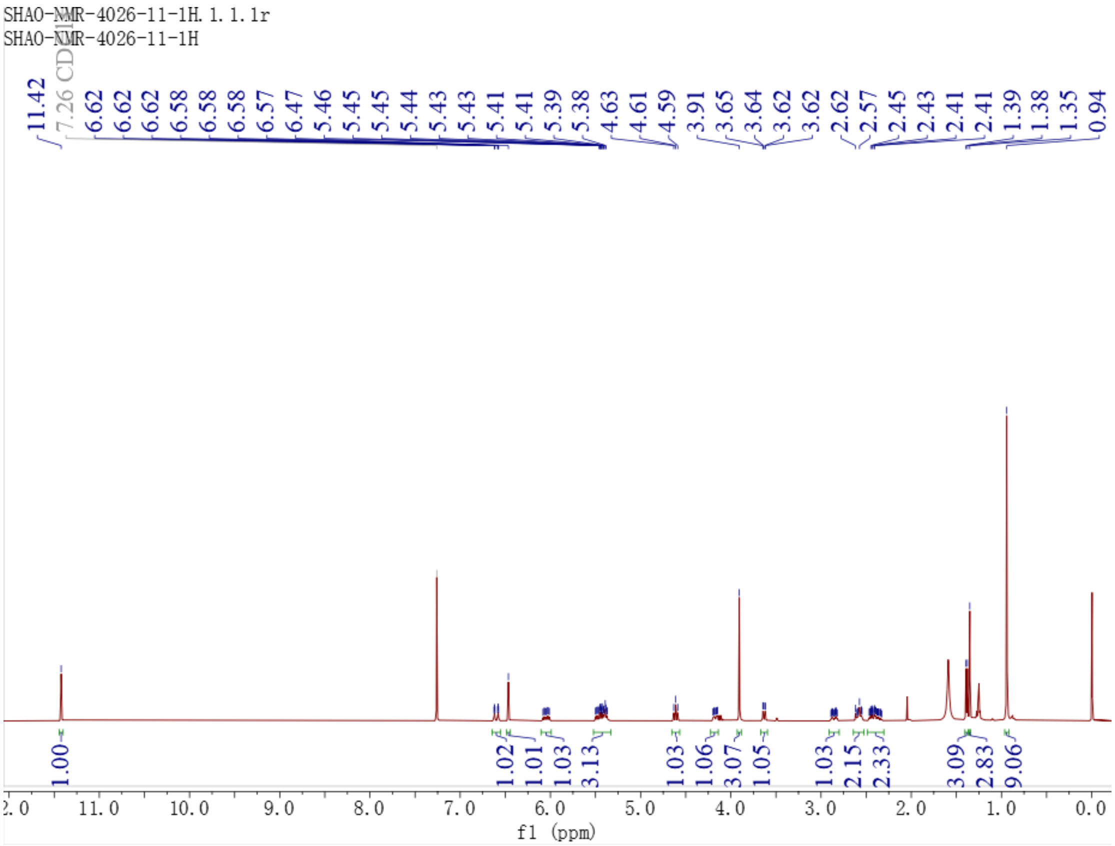

Figure S90. ^{1}H NMR (400 MHz, Chloroform-*d*) spectrum of compound **35.**

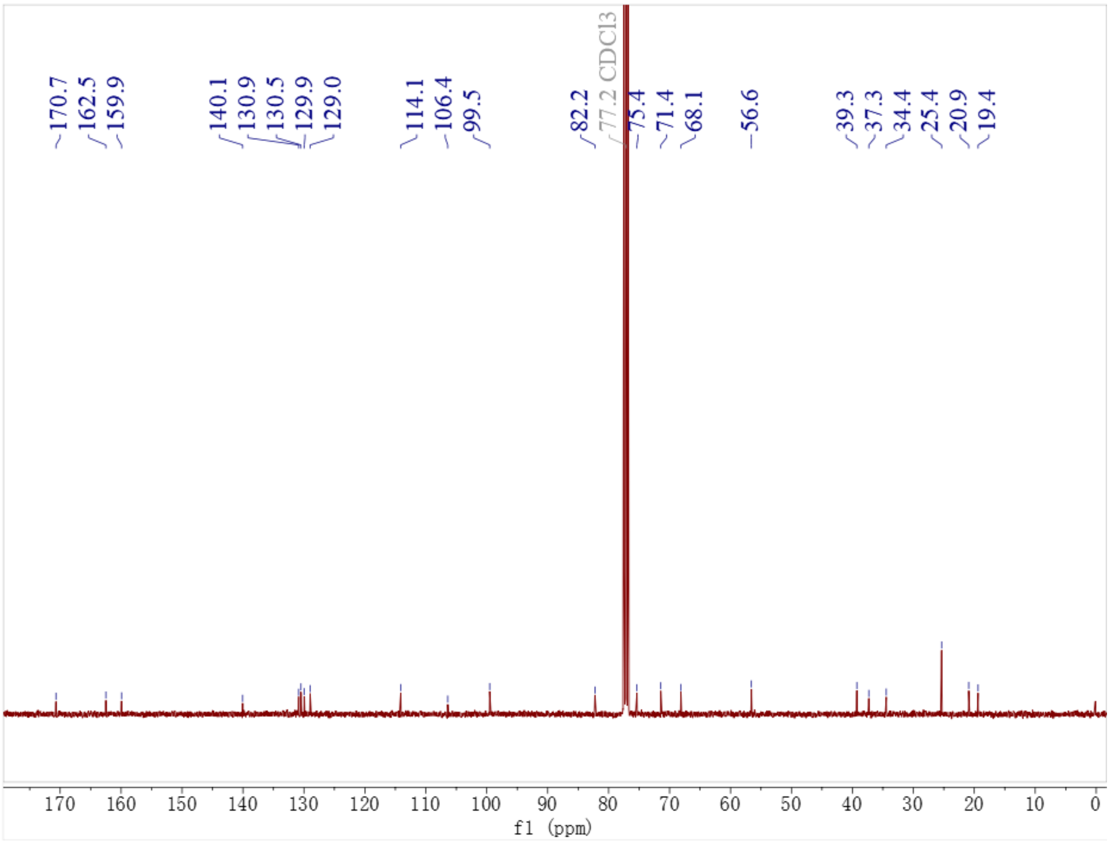

Figure S91. ^{13}C NMR (100 MHz, Chloroform-*d*) spectrum of compound **35.**

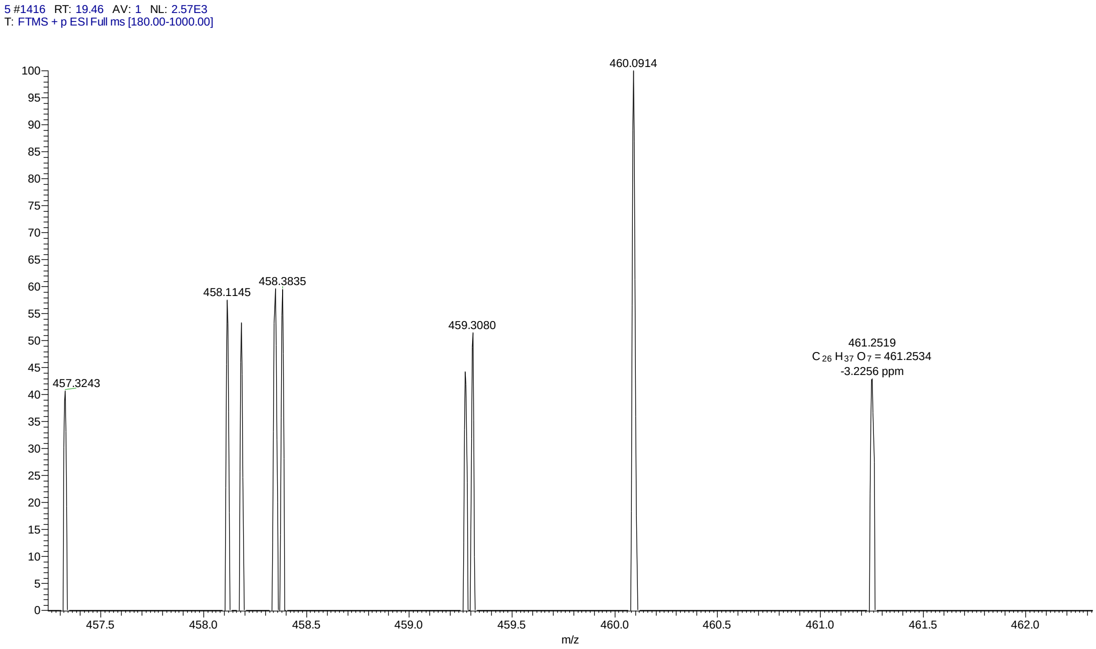

Figure S92. HR-ESI-MS spectrum of compound **35**.

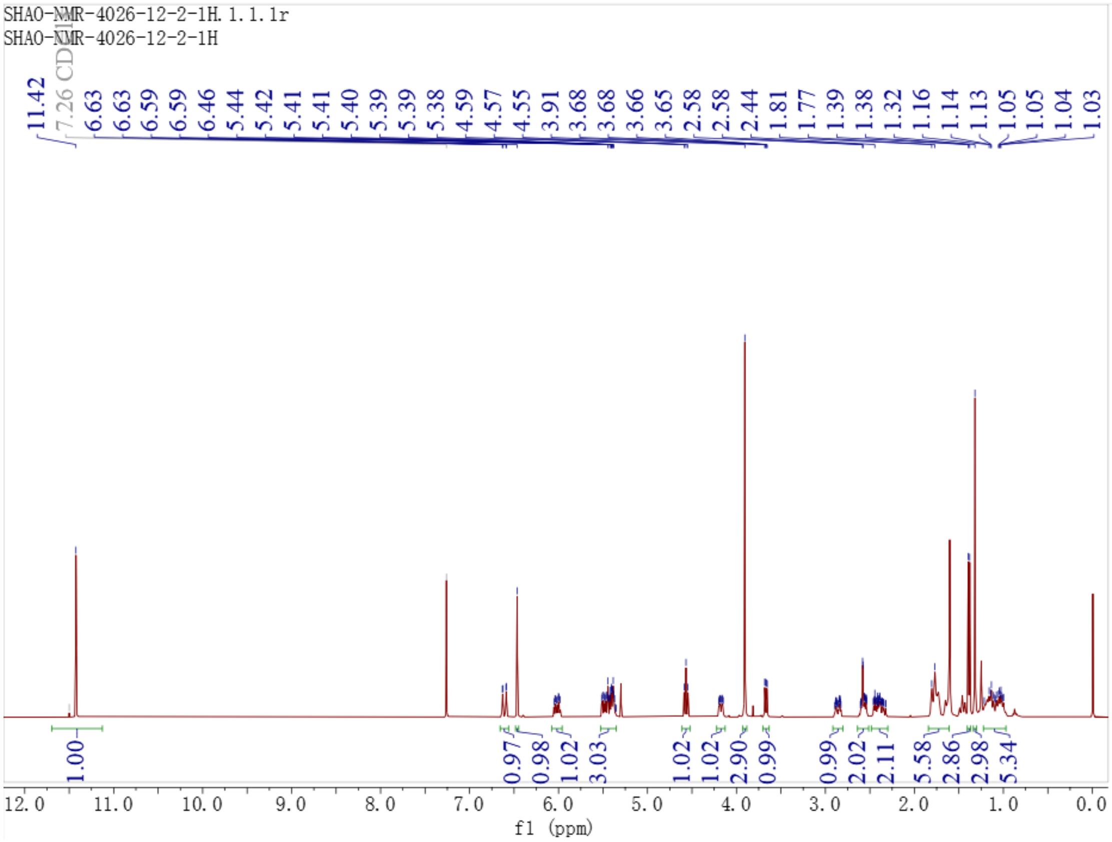

Figure S93. ^{1}H NMR (400 MHz, Chloroform-*d*) spectrum of compound **36**.

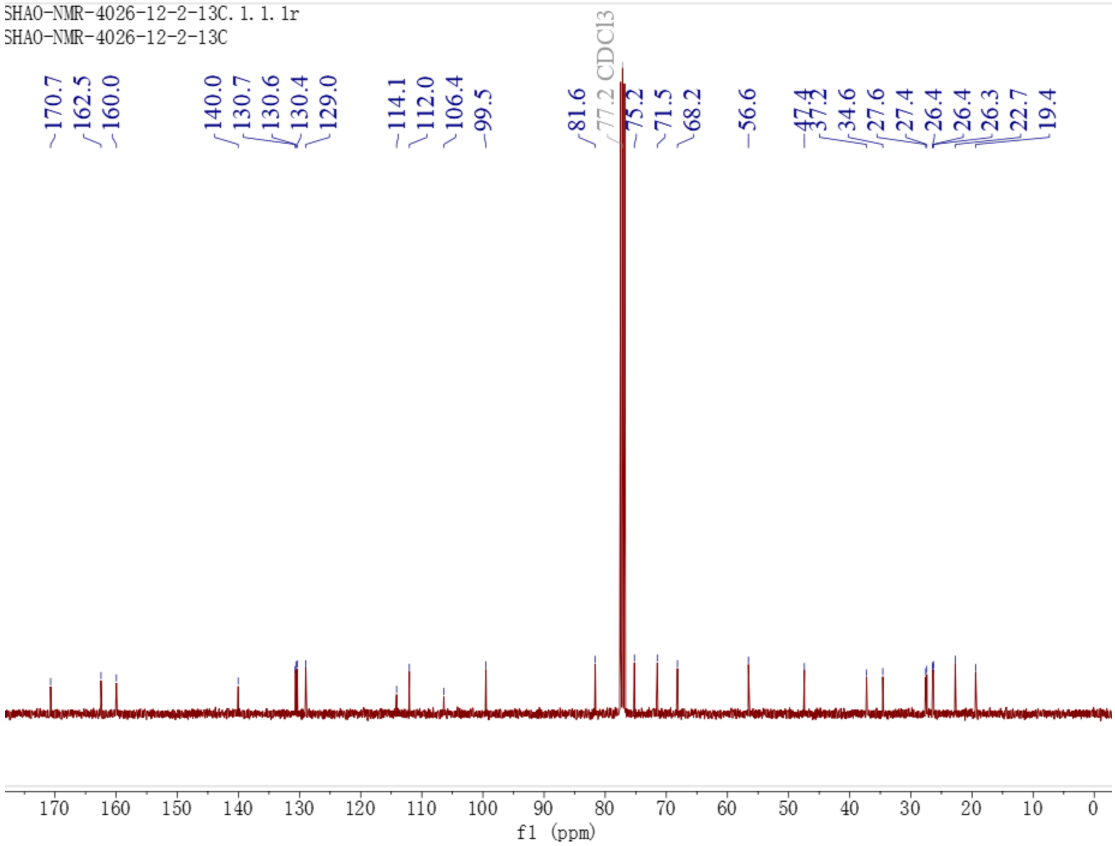

Figure S94. ^{13}C NMR (100 MHz, Chloroform-*d*) spectrum of compound **36**.

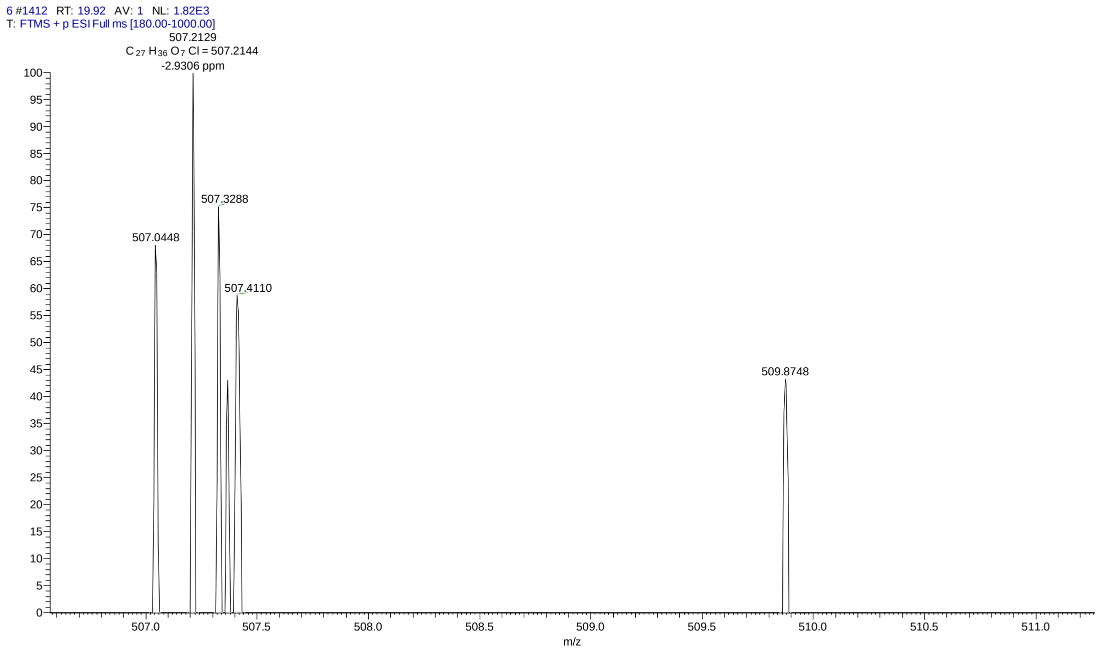

Figure S95. HR-ESI-MS spectrum of compound **36**.

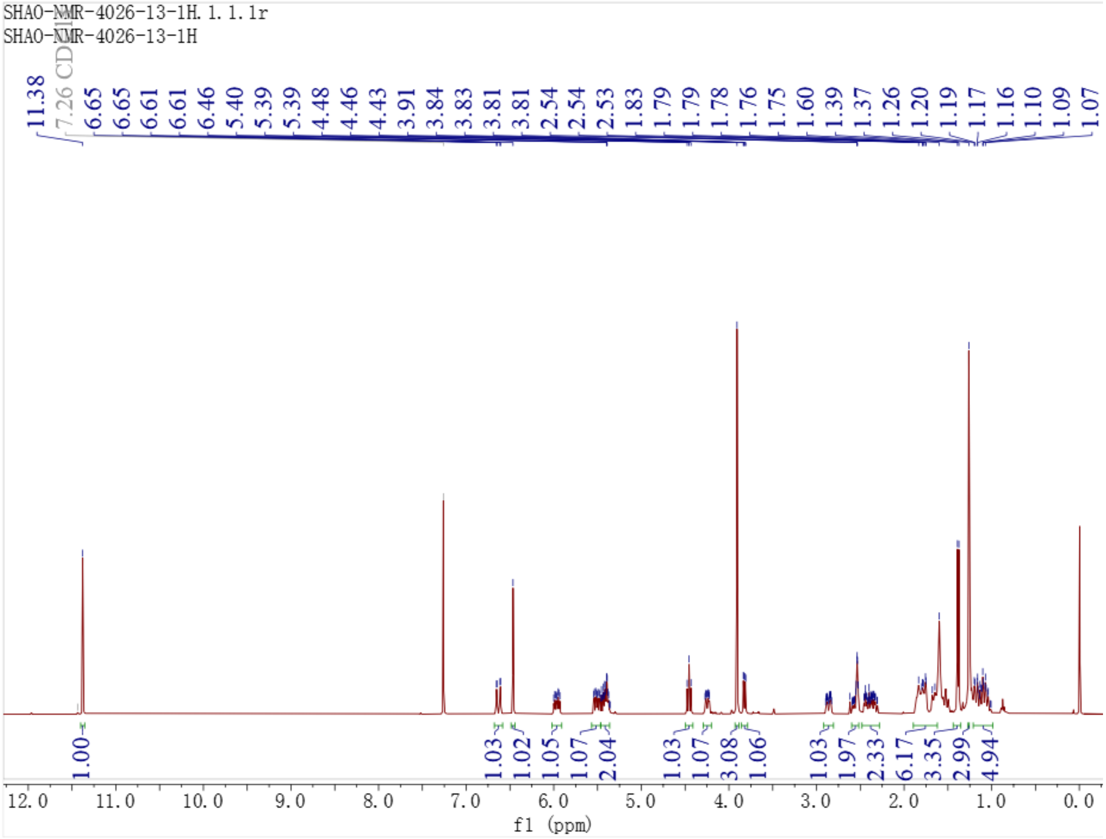

Figure S96. ^{1}H NMR (400 MHz, Chloroform-*d*) spectrum of compound **37.**

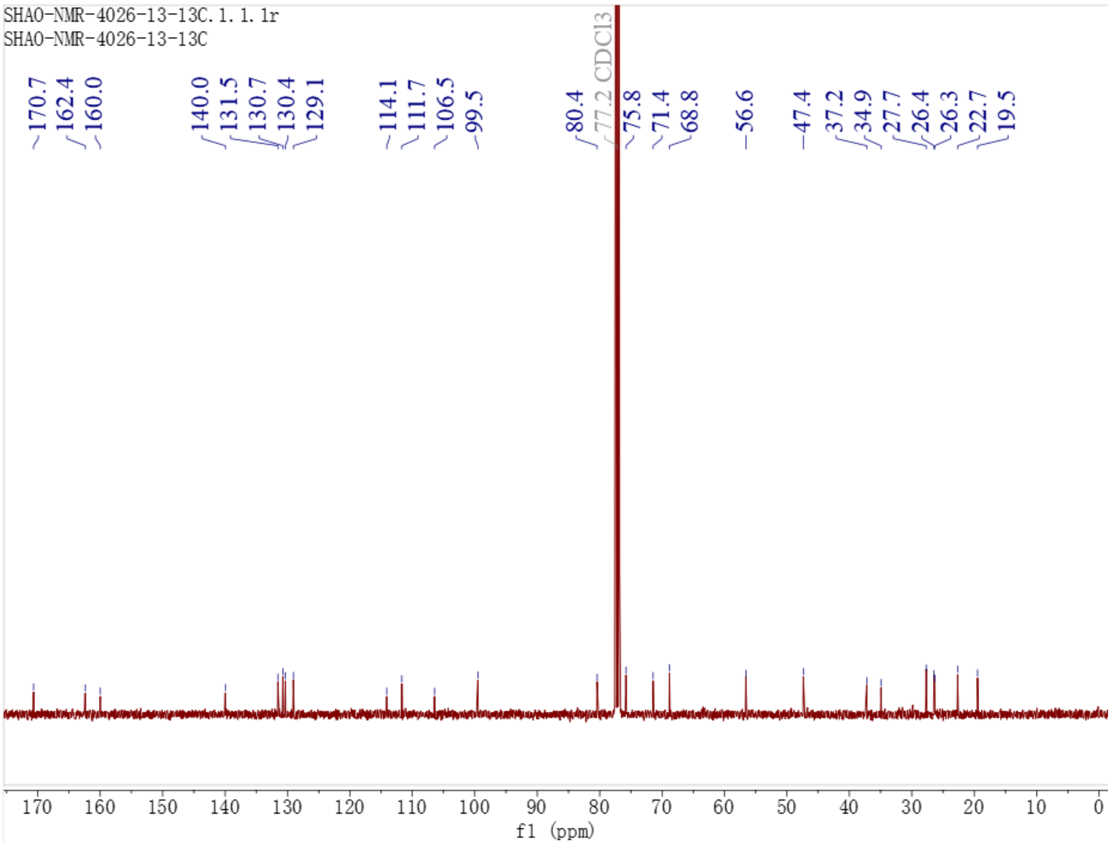

Figure S97. ^{13}C NMR (100 MHz, Chloroform-*d*) spectrum of compound **37**.

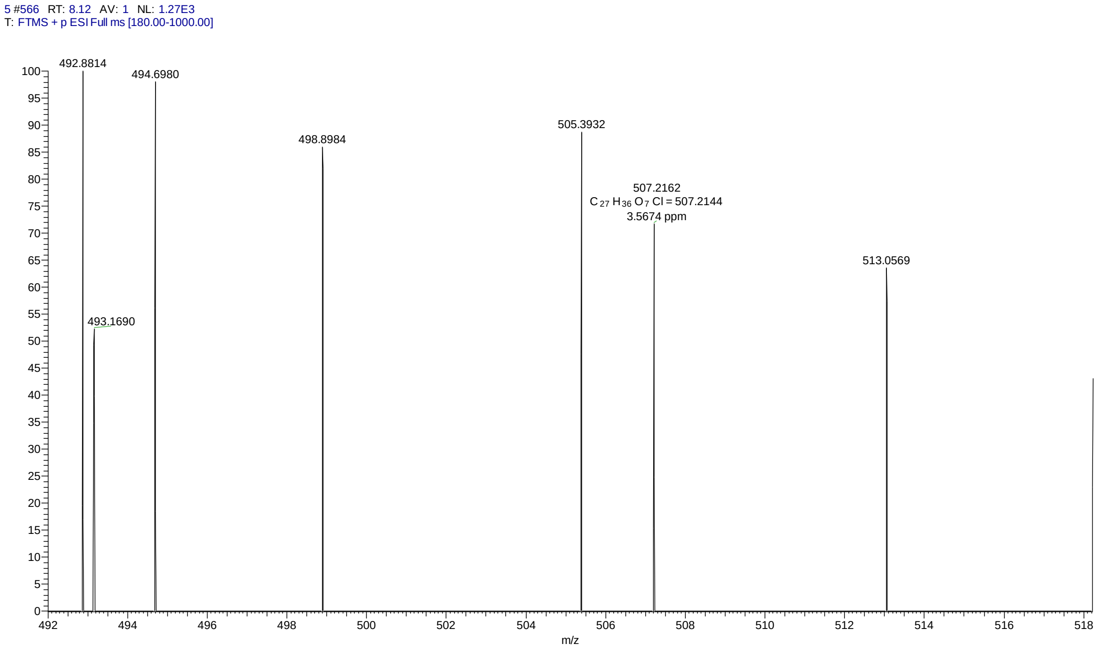

Figure S98. HR-ESI-MS spectrum of compound **37**.

.
